# Isolation and characterization of *Salmonella enterica* serovars from poultry in Egypt: a comprehensive genetic analysis of ESBLs, MCR, integron and other resistance genes

**DOI:** 10.1186/s12917-025-05121-z

**Published:** 2025-11-21

**Authors:** Mona Salem, Akram Ahmed Hassan Al-Khalidi, Enas Hammad, Gamal Younis, Amal Awad

**Affiliations:** 1 Department of Bacteriology, Mycology and Immunology, Faculty of Veterinary Medicine, Mansoura University, Mansoura, 35516 Egypt; 2 Department of Pathology, College of Veterinary Medicine, University of Diyala, Baqubah, Iraq; 3 Agricultural Research Center (ARC), Animal Health Research Institute–Mansoura Provincial Lab (AHRI-Mansoura), Giza, Egypt

**Keywords:** *Salmonella* jerusalem, *Salmonella* colorado, Broiler chicks, ESBLs, AmpC, MBLs, *mcr*-1, *intI*1, MDR

## Abstract

**Objectives:**

*Salmonella enterica* is a major foodborne pathogen increasingly associated with antimicrobial resistance, particularly in poultry production. The present study aimed to investigate the prevalence and serotypes of *Salmonella* isolated from broiler chicks. Additionally, the recovered strains were analyzed to determine their antimicrobial susceptibility profiles and to identify the presence of genotypic resistance determinants.

**Design:**

A total of 450 samples were collected from broiler chicks submitted by poultry farms and diagnostic laboratories in Mansoura, Egypt. Confirmed *Salmonella* isolates were serotyped and subjected to antimicrobial susceptibility testing. Colistin susceptibility was determined by the broth microdilution method (MIC). In addition, polymerase chain reaction (PCR) was employed to screen the genetic elements associated with antimicrobial resistance.

**Results:**

Twenty-nine isolates (*n* = 29) were confirmed as *Salmonella*. Serotyping revealed the presence of the following *Salmonella* serovars: *S*. Kentucky (*n* = 10), *S*. Derby (*n* = 6), *S*. Typhimurium (*n* = 4), *S.* Salamae (*n* = 3), *S.* Colorado (*n* = 2), *S*. Infantis (*n* = 2), *S*. Jerusalem (*n* = 1), and *S.* Virchow (*n* = 1). All isolates were resistant to cefoxitin, cefepime, ceftazidime, nalidixic acid, erythromycin, and fosfomycin, while the highest susceptibility was observed to meropenem and imipenem. Eight isolates were resistant to colistin (MIC > 2 µg/mL). Molecular detection of resistance genes demonstrated the presence of *bla*_TEM_ (82.8%), *bla*_OXA−10_ (27.6%), *bla*_SHV_ (24.1%), *bla*_CTX−M_ (24.1%), *bla*_CMY−2_ (10.3%), and *bla*_OXA−2_ (3.4%), whereas *bla*_VEB−1_ was not detected in any of the isolates. In addition, all carbapenemase-encoding genes were not detected. Among the colistin resistance genes, only *mcr*-1 was detected (10.3%), while the class I integron (*intI*1) was detected in 96.6% of the isolates.

**Conclusions:**

Based on serological identification, this study reports the detection of rare *Salmonella enterica* serovars, such as *S*. Colorado and *S*. Jerusalem, with extensive drug resistance (XDR) in broiler farms in Egypt. These isolates carried critical resistance genes, including *intI*1, *mcr*-1, and various β-lactamases. The findings underscore the urgent need for surveillance, biosecurity, and antimicrobial stewardship to protect public health and poultry production.

**Graphical abstract:**

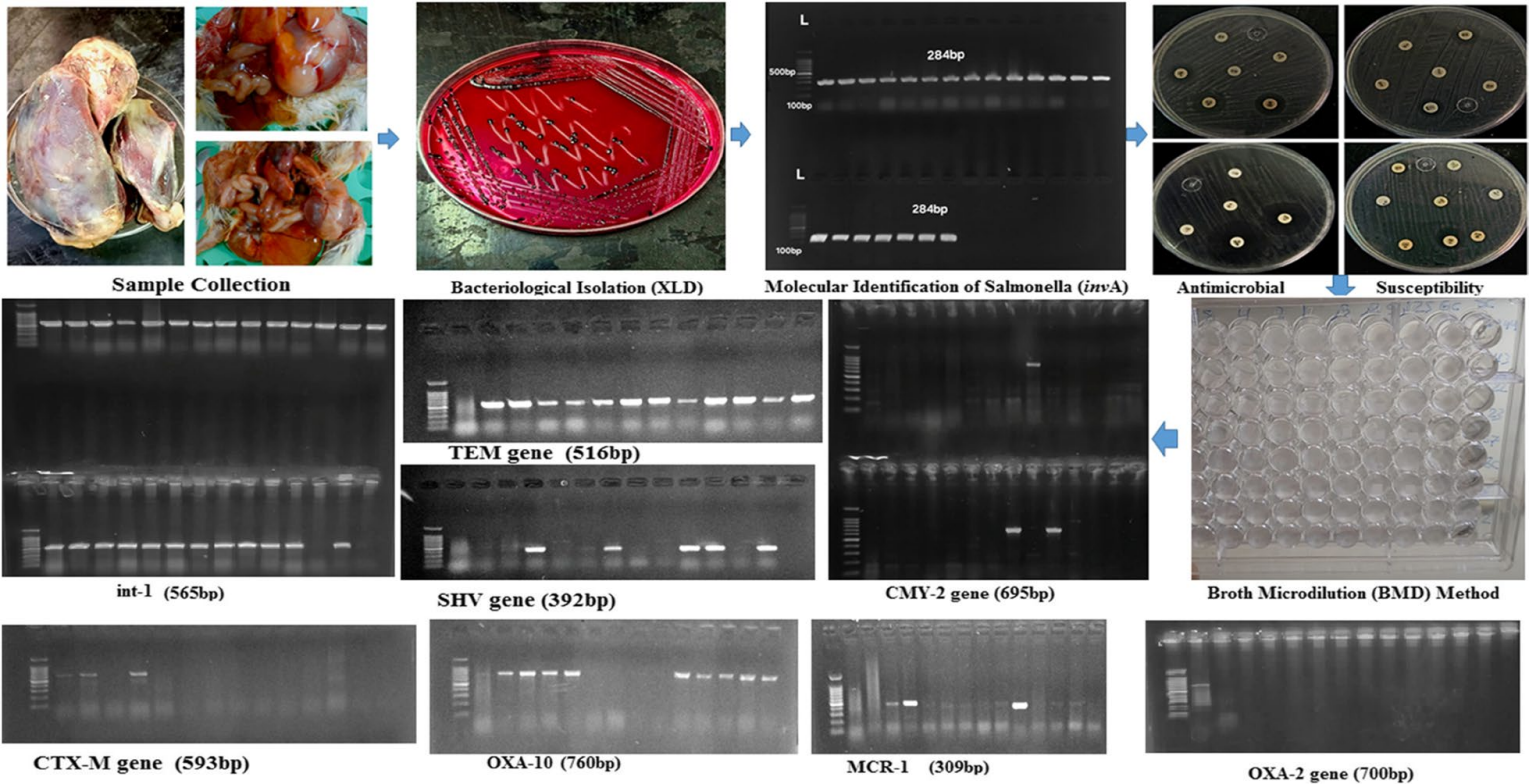

**Supplementary Information:**

The online version contains supplementary material available at 10.1186/s12917-025-05121-z.

## Introduction

Poultry production is a crucial sector in Egypt’s agriculture, providing an affordable source of high-quality protein and contributing significantly to food security and the economy. Egypt produces approximately 1.4 million tons of poultry meat annually and 13 billion table eggs [1]. Reports confirm that Egypt is one of the largest producers of poultry meat and eggs in Africa, playing a crucial role in the region’s food supply chain [2]. Moreover, the poultry industry employs millions of people, ranging from farmers to workers in processing and distribution [3].

However, one of the greatest threats to poultry production worldwide, including in Egypt, is *Salmonella* infection. *Salmonella* not only affects the health and productivity of poultry but also poses serious food safety concerns for humans through consumption of contaminated poultry products [4]. Worldwide, *Salmonella* is responsible for approximately 1.3 billion infections and 155,500 deaths each year [5]. Infections in humans typically cause salmonellosis, which manifests as diarrhea, fever, and abdominal cramps, with severe cases requiring hospitalization [6]. It has been reported that the poultry sector alone is responsible for up to 50% of salmonellosis outbreaks [7]. The bacterium can persist in various environments, including contaminated feed, water, and farm equipment, which hinders its eradication. Moreover, it can colonize the intestinal tract of birds without causing clinical symptoms, making its detection and control within farms particularly challenging [4].

Antibiotics are necessary for the therapeutic management of diseases in both humans and animals, as well as for enhancing food safety, animal welfare, and production. However, the extensive use and misuse of antimicrobial agents in poultry farms have significantly contributed to the emergence and dissemination of antimicrobial resistance (AMR) [8]. The emergence of antibiotic-resistant *Salmonella* strains represents a significant public health challenge, especially when associated with the poultry sector, which serves as a major reservoir of zoonotic pathogens. The extensive application of antibiotics in poultry production, for both therapeutic and growth-promoting purposes, has facilitated the development and transmission of resistant *Salmonella* strains. These strains can be transmitted to humans via the food chain, resulting in infections that are increasingly difficult to treat [9].

*Salmonella* spp. isolated from poultry have increasingly exhibited resistance to β-lactam antibiotics, primarily due to the production of ESBLs, AmpC β-lactamases, and, more recently, metallo-β-lactamases (MBLs). ESBLs, such as TEM, SHV, and CTX-M enzymes, hydrolyze a wide range of β-lactam antibiotics, rendering them ineffective [10]. VEB-1, a less common ESBL gene, has also been identified in *Salmonella* isolates and is capable of hydrolyzing a wide range of β-lactam antibiotics. Additionally, enzymes like OXA-10 and OXA-2 can confer resistance to β-lactams and have been increasingly reported in poultry-associated strains [11]. CMY-2, an AmpC-type β-lactamase, confers resistance to cephamycins and other extended-spectrum cephalosporins [10]. MBLs such as GES, OXA-48, IMP, VIM, KPC, and NDM-1 represent an even more alarming resistance mechanism, as they can hydrolyze carbapenems, considered last-resort antibiotics, and are resistant to nearly all β-lactams. Although the occurrence of MBLs in *Salmonella* from poultry remains relatively low compared to other resistance mechanisms, their detection represents an emerging public health concern [12].

Colistin is a last-resort antibiotic used to treat infections caused by multidrug-resistant (MDR) Gram-negative bacteria. It works by disrupting the bacterial outer membrane, leading to cell death. Due to its critical role in human medicine, the emergence of colistin resistance is considered a major public health concern. One of the primary mechanisms for colistin resistance is the presence of mobilized colistin resistance (*mcr)* genes, especially *mcr*−1 [13]. Since the discovery of *mcr*−1, multiple variants (*mcr*−1 to *mcr*−10) have been identified in various bacterial species, including *Salmonella*, *Klebsiella*, and *Pseudomonas* [14]. These genes are often located on plasmids, facilitating horizontal gene transfer between bacterial strains and accelerating their global dissemination [15]. Furthermore, *mcr* genes frequently co-exist with other antibiotic resistance genes (ARGs), contributing to the development of extensively drug-resistant (XDR) or even pan-drug-resistant (PDR) bacterial strains [16, 17].

Integrons are genetic elements that play a significant role in the spread of antibiotic resistance among bacteria, including *Salmonella* species. They act as gene capture systems, allowing bacteria to acquire and express resistance genes through gene cassettes. In *Salmonella* isolated from poultry, class 1 integrons are the most commonly detected type. The interplay between ESBL genes, *mcr*, and integrons plays a crucial role in the dissemination of antibiotic resistance. Understanding these genetic interactions is critical for developing effective control strategies to combat antibiotic-resistant *Salmonella* in poultry production [18, 19].

Therefore, this study aimed to isolate and characterize *Salmonella enterica* from poultry in Egypt, with a particular focus on assessing the prevalence of different serovars, including potentially novel types. In addition, the study sought to evaluate the resistance of the isolates to a broad range of antimicrobial agents in order to generate comprehensive data on the antimicrobial resistance landscape in Egyptian poultry farms and to investigate key antimicrobial resistance determinants such as ESBLs, *mcr* genes, and class 1 integrons. These findings offer valuable insights into the genetic resistance profiles of *Salmonella* strains circulating in Egyptian poultry and underscore their potential public health impact.

## Materials and methods

### Ethical approval

Prior to conducting the study, ethical approval was obtained from the Research Ethics Committee of the Faculty of Veterinary Medicine, Mansoura University, Egypt. Protocol code: MU-ACUC (VM.PhD.23.10.24). The study adhered to international ethical guidelines to ensure the welfare of poultry and the safety of farm workers, and informed consent was obtained from all farm owners.

### Sample collection

A total of 450 samples from apparently healthy (*n* = 100), diseased (*n* = 200), and freshly dead birds (*n* = 150) were randomly collected from poultry farms and diagnostic labs in Mansoura, Kafr El-Shaikh, Damietta, surrounding rural areas, and the Cairo-Alexandria Desert Road, Egypt. Samples were collected from broiler flocks (White and Sasso breeds) aged 1–35 days for white and 50 days for sasso, during February to April 2024.Samples included cloacal swabs (*n* = 150), unabsorbed yolk sacs (*n* = 50), and internal organs such as liver, intestines, spleen, and heart (*n* = 250). Sampling was performed based on the availability of birds submitted to the diagnostic laboratory, with the number of collected samples varying according to flock size and submission frequency, rather than following a fixed weekly or age-based sampling scheme.

Data regarding antibiotic treatment history and associated husbandry practices were collected through a structured questionnaire (Supplementary file 1). The questionnaire was designed to obtain information on poultry farmers’ demographics, husbandry practices, patterns of antibiotic use, and their knowledge of *Salmonella* spp.

Most of the sampled birds exhibited clinical signs suggesting *Salmonella* infection, including watery, yellowish-green diarrhea, weakness, loss of appetite, dehydration, ruffled feathers, and respiratory distress in some cases. Post-mortem examination revealed common pathological lesions such as enteritis, hepatomegaly with focal necrosis, pericarditis, splenomegaly, and omphalitis. The presence of unabsorbed yolk sacs with signs of inflammation was frequently observed, particularly in younger chicks. To ensure humane treatment, diseased chicks were euthanized by cervical dislocation in accordance with approved animal welfare guidelines. All samples were collected under strict aseptic conditions in sterile, labeled containers to prevent cross-contamination, in accordance with the OIE guidelines for sample collection. Immediately after collection, samples were maintained under refrigeration and transferred to the Department of Bacteriology, Mycology, and Immunology at the Faculty of Veterinary Medicine, Mansoura University, Egypt, for further microbiological investigation and bacterial isolation.

### Bacteriological isolation and identification of *Salmonella *spp

Under strictly aseptic conditions, cloacal swabs along with pooled visceral organs (unabsorbed yolk sac, liver, heart, and spleen) were inoculated into 9 mL of Buffered Peptone Water (BPW) (Oxoid Ltd, Hampshire, England) and incubated aerobically at 37 °C for 18 h. Intestinal samples were processed separately to avoid cross-contamination, following the same pre-enrichment and incubation protocol. Following pre-enrichment, a 0.1 mL aliquot from the pre-enriched Buffered Peptone Water was aseptically inoculated into 9 mL of Rappaport-Vassiliadis (RV) enrichment broth (Oxoid Ltd, Hampshire, England), followed by incubation at 42 °C for 18 h. Post-enrichment, the cultures were streaked onto Xylose-Lysine-Desoxycholate agar (XLD) and MacConkey’s agar (Oxoid Ltd, Hampshire, England), followed by aerobic incubation at 37 °C for 18–24 h (ISO 6579:2002 standards). Presumptive *Salmonella* colonies were preliminarily identified based on their typical morphology, appearing as red colonies with black centers on XLD agar and as pale, non-lactose-fermenting colonies on MacConkey’s agar [20]. Suspected *Salmonella* isolates were further confirmed by Gram staining and a series of biochemical tests, including the Triple Sugar Iron (TSI) test, Oxidase test, Indole test, Methyl Red-Voges Proskauer (MR-VP) test, Simmons citrate test, and Urease test [21]. Additionally, isolates were preserved in Tryptic Soy Broth (TSB) with 20% glycerol at −80 °C for further molecular characterization and antimicrobial susceptibility testing.

### Molecular identification of *Salmonella* isolates

Genomic DNA was extracted from biochemically confirmed *Salmonella* isolates using the boiling method as described by Queipo et al. [22]. In summary, three to five suspected *Salmonella* colonies grown on XLD agar were subcultured into 3 mL of Tryptic Soy Broth (TSB) and incubated aerobically at 37 °C for 24 h. After incubation, 1 mL of the culture was transferred into a sterile microcentrifuge tube and centrifuged at 12,000 × g for 6 min to harvest the bacterial cells. The resulting supernatant was discarded, and the cell pellet was resuspended in 100 µL of nuclease-free water. For cell lysis, the suspension was subjected to heat treatment at 94 °C for 15 min by using a thermal block (Biometra). After heating, the lysate was centrifuged again at 12,000 × g for 5 min, and the resulting supernatant containing the extracted DNA was carefully transferred to a new sterile tube and stored at −20 °C for subsequent molecular analyses. DNA concentration and purity were assessed by measuring 1 µL of each sample using a NanoDrop 1000 spectrophotometer (Thermo Fisher, USA) at an absorbance ratio of 260/280 nm.

For molecular confirmation of *Salmonella* isolates (*n* = 29), PCR was performed targeting the *inv*A gene, a conserved virulence marker specific for *Salmonella*, based on the protocol of Nadi et al. [23]. The primer sequence and expected product size are summarized in Table 1. The PCR reaction was carried out in a final volume of 25 µL, consisting of 5 µL of extracted DNA, 1 µL of each primer (10 pmol) (Metabion), 5.5 µL of nuclease-free water, and 12.5 µL of DreamTaq Green Master Mix (Thermo Scientific, USA). Amplification was carried out using a 2720 Thermal Cycler (Applied Biosystems, Thermo Scientific, USA) using optimized thermal cycling parameters. PCR amplicons were analyzed via electrophoresis on a 1% agarose gel prepared in 1X TBE buffer, stained with ethidium bromide, and run at 100 V for 45 min. A 1000 bp DNA ladder (Qiagen, USA) was used as a molecular size marker. DNA bands were visualized under UV light using a transilluminator system (Cleaver Scientific Ltd, UK). A negative control (nuclease-free water) was included in each run to confirm the specificity of the amplification.

**Table 1:** Oligonucleotide primers used in this study

Target gene	Primer direction and sequence	Amplicon size (bp)	Encoded	Reference
***inv*** A	F: GTGAAATTATCGCCACGTTCGGGCAA R: TCATCGCACCGTCAA AGGAACC	284	Type III Secretion System (T3SS-1)	[23]
***bla*** TEM ***bla*** SHV ***bla*** CTX−M ***bla***_VEB−1_	F: ATCAGCAATAAACCAGC R: CCCCGAAGAACGTTTTC F: AGGATTGACTGCCTTTTTG R: ATTTGCTGATTTCGCTCG F: ATG TGCAGYACCAGTAARGTKATG GC R: TGGGTRAARTARGTSACCAGAAYCAGC GG F: CGACTTCCATTTCCCGATGC R: GGACTCTGCAACAAATACGC	516 392 593 642	(ESBLS) Extended-Spectrum Beta-Lactamases	[30] [30] [31] [32]
***bla***_OXA−10_ ***bla***_OXA−2_	F: TATCGCGTGTCTTTCGAGTA R: TTAGCCACCAATGATGCCC F: GCCAAAGGCACGATAGTTGT R: GCGTCCGAGTTGACTGCCGG	760 700	(OXA) Oxacillinase-Like Beta-Lactamases	[32] [33]
***bla***_CMY−2_	F: AGCGATCCGGTCACGAAATA R: CCCGTTTTATG CACCCATGA	695	AmpC Beta-Lactamase	[34]
***bla***_***GES***_ ***bla***_***OXA−48***_ ***bla***_***IPM***_ ***bla***_***VIM***_ ***bla***_***KPC***_ ***bla***_***NDM−1***_	F: AGTCGGCTAGACCGGAAAG R: TTTGTCCGTGCTCAGGAT F: GCTTGATCGCCCTCGATT R: GATTTGCTCCGTGGCCGAAA F: TTGACACTCCATTTACDG R: GATYGAGAATTAAGCCACYCT F: GATGGTGTTTGGTCGCATA R: CGAATGCGCAGCACCAG F: CATTCAAGGGCTTTCTTGCTGC R: ACGACGGCATAGTCATTTGC F: CAATATTATGCACCCGGTCG R: ATCATGCTGGCCTTGGGGAA	399 438 232 390 798 287	Carbapenem resistance genes	[35] [36] [36] [36] [37] [36]
***mcr*** −1 ***mcr*** −2 ***mcr*** −3 ***mcr*** −4 ***mcr*** −5 ***mcr*** −7.1 ***mcr*** −9	F: CGGTCAGTCCGTTTGTTC R: CTTGGTCGGTCTGTA GGG F: TGTTGCTTGTGCCGATTGGA R: AGATGGTATTGTTGGTTGCTG F: TTGGCACTGTATTTTGCATTT R: TTAACGAAATTGGCTGGAACA F: ATTGGGATAGTCGCCTTTTT R: TTACAGCCAGAATCATTATCA F: TATCTCGACAAGGCCATGCTG R: G AGGGGATAAACCGACCCTGA F: TGATCTCGATGTTGGGCACC R: AATCTGGCGTTCGTCGTAGT F: TTCCCTTTGTTCTGGTTG R: GCAGGTAATAAGTCGGTC	309 567 542 488 613 335 1011	Mobile Colistin resistance genes	[38] [38] [38] [38] [38] [38] [39]
***intI*** 1	F: GCCTTGCTGTTCTTCTACGG R: GATGCCTGCTTGTTCTACGG	565	Integrase Class 1	[40]

To further validate the PCR products, the *inv*A amplicons were purified using the QIAquick Gel Extraction Kit (Qiagen, Germany) in accordance with the manufacturer’s instructions. The purified DNA was then submitted for Sanger sequencing at Microsynth Seqlab GmbH, Göttingen, Germany. The obtained *inv*A gene sequences were aligned using BioEdit software and subsequently analyzed using the BLAST tool https://blast.ncbi.nlm.nih.gov/Blast.cgi to identify homology with reference sequences available in the NCBI database. A phylogenetic tree was then constructed using the neighbor-joining algorithm in MEGA version 11 to determine the genetic relatedness among the *Salmonella* isolates. The sequences were deposited in GenBank under the accession numbers PQ720689, PQ720690, and PQ720691 (Table 2).

**Table 2:** Sequence identification and corresponding GenBank accession numbers

Accession Number	Identification
PQ720689	*Salmonella enterica subsp*. *enterica* serovar Colorado strain M6 invasion protein (*inv*A) gene, partial cds
PQ720690	*Salmonella enterica subsp. enterica* serovar Jerusalem strain M8 invasion protein (*inv*A) gene, partial cds
PQ720691	*Salmonella enterica subsp. enterica* serovar Colorado strain M9 invasion protein (*inv*A) gene, partial cds
PQ678532	*Salmonella enterica subsp. enterica* serovar Jerusalem TEM family class A beta-lactamase (*bla*_*TEM*_) gene, partial cds
PQ678533	*Salmonella enterica subsp. enterica* serovar Colorado TEM family class A beta-lactamase (*bla*_*TEM*_) gene, partial cd
PQ720692	*Salmonella enterica subsp. enterica* serovar Colorado SHV family class A beta-lactamase (*bla*_*SHV*_) gene, partial cds
PQ720693	*Salmonella enterica subsp. enterica* serovar Jerusalem SHV family class A beta-lactamase (*bla*_*SHV*_) gene, partial cds
PQ720694	*Salmonella enterica subsp. enterica* serovar Jerusalem CTX-M family beta-lactamase (*bla*_CTX−M_) gene, partial cds
PQ659181	*Salmonella enterica subsp. enterica* serovar Kentucky CMY family beta-lactamase (*CMY* −2) gene, partial cds
PQ659182	*Salmonella enterica subsp. enterica* serovar Jerusalem MCR family phosphoethatanolamine-lipid A transferase (*MCR* −1) gene, partial cds
PQ659183	*Salmonella enterica subsp. enterica* serovar Kentucky MCR family phosphoethatanolamine-lipid A transferase (*MCR* −1) gene, partial cds
PQ659184	*Salmonella enterica subsp. enterica* serovar Jerusalem class 1 integron integrase *IntI*1 (*IntI*1) gene, partial cds

### Serological identification of *Salmonella *isolates

Confirmed *Salmonella* isolates were serotyped using the slide agglutination technique with commercial antisera (SISIN, Berlin), according to the Kauffmann–White classification scheme (ISO 6579:2014). Serotyping analysis was carried out at the Serology Unit of the Animal Health Research Institute located in Dokki, Egypt, which is an ISO/IEC 17,025 accredited facility by the Egyptian Accreditation Council (EGAC). Serotyping was conducted following the manufacturer’s protocol (Lillidale^®^, United Kingdom). Initially, all isolates underwent preliminary screening using the omnivalent A-67 antisera for presumptive identification. Isolates that yielded positive results were further analyzed with monovalent anti-*Salmonella* O antisera, covering serogroups A to E and F-67. For more specific identification, additional testing was performed using anti-*Salmonella* O antisera targeting individual somatic (O) antigens (e.g., 2, 4, 7, 8, etc.). To complete the serotyping process, flagellar (H) antigens in both phase 1 and phase 2 were identified using grouped anti-H antisera

### Antimicrobial susceptibility testing of *Salmonella* serovars

The in vitro antimicrobial susceptibility of the *Salmonella* isolates (*n* = 29) was assessed using the Kirby–Bauer disc diffusion method [24] following the Clinical and Laboratory Standards Institute (CLSI, 2024) guidelines [25] (Table 3). Antimicrobial discs (*n* = 22) (Oxoid Ltd, Hampshire, England) representing multiple antimicrobial classes (*n* = 11) of both human and veterinary relevance were used. The selection of antimicrobial discs in the present study was based on several prescriptions by local veterinarians targeting *Salmonella* infections in local chicken farms. They include: β-lactams (Aminopenicillin): Amoxicillin (AX, 25 µg), Ampicillin (AM, 10 µg), β-lactams (Monobactam): Aztreonam (ATM, 10 µg), β-lactamase inhibitor: Amoxicillin-clavulanic (AMC, 30 µg), β-lactams (Cephalosporin): Cefoxitin (FOX, 30 µg), Ceftriaxone (CTR, 30 µg), Ceftazidime (CAZ, 30 µg), Cefepime (CPM/FEP), β-lactams (Carbapenems): Imipenem (IPM, 10 µg), Meropenem (MEM, 10 µg), Phenicol: Chloramphenicol (C, 30 µg), Phosphonic acid derivatives: Fosfomycin (FF, 30 µg), Tetracyclines: Doxycycline (DO, 30 µg), Fluoroquinolones: Ciprofloxacin (CIP, 5 µg), Quinolones: Nalidixic acid (NA, 30 µg), Macrolides: Erythromycin (E, 15 µg), Aminoglycosides: Amikacin (AK, 30 µg), Apramycin (APR, 15 µg), Gentamicin (CN, 10 µg), Kanamycin (K, 30 µg), and Streptomycin (S, 10 µg), DHFR inhibitor + Sulfonamides: Trimethoprim/sulfamethoxazole (SXT, 25 µg) (Supplementary file 2: Table S1)

**Table 3:** Interpretation and classification of antimicrobial susceptibility for *Salmonella* serovars according to CLSI/NCCLS guidelines and their medical importance according to WHO, (2024)

Antimicrobial	Disc Conc.	Group	Zone Diameter (mm) (*R* ≤/I/S ≥)	Category	Medical Importance (WHO, 2024) [28]
Amoxicillin (AX)	25 µg	β-lactams (Aminopenicillins)	≤ 13/14–16/≥17	II	Highly Important
Ampicillin (AM)	10 µg	β-lactams (Aminopenicillins)	≤ 13/14–16/≥17	II	Highly Important
Aztreonam (ATM)	10 µg	β-lactams (Monobactam)	≤ 17/18–20/≥21	I	Critically Important
Amoxicillin-clavulanic (AMC)	30 µg	Penicillin + β-lactamase inhibitor	≤ 13/14–17/≥18	II	Highly Important
Cefoxitin (FOX)	30 µg	Cephalosporin (2nd gen)	≤ 13/14–20/≥21	II	Highly Important
Ceftriaxone (CTR)	30 µg	Cephalosporin (3rd gen)	≤ 19/20–22/≥23	I	Highest priority critically important
Ceftazidime (CAZ)	30 µg	Cephalosporin (3rd gen)	≤ 17/18–20/≥21	I	Highest priority critically important
Cefepime (CPM/FEP)	30 µg	Cephalosporin (4th gen)	≤ 17/18–20/≥21	I	Highest priority critically important
Imipenem (IPM)	10 µg	Carbapenems	≤ 19/20–22/≥23	I	Highest priority critically important
Meropenem (MEM)	10 µg	Carbapenems	≤ 14/15–17/≥18	I	Highest priority critically important
Chloramphenicol (C)	30 µg	Phenicol	≤ 12/13–17/≥18	II(	Highly Important
Fosfomycin (FF)	30 µg	Phosphonic acid derivatives	≤ 11/12–14/≥15	I	Highest priority critically important
Doxycycline (DO)	30 µg	Tetracyclines	≤ 12/13–15/≥16	II	Highly Important
Ciprofloxacin (CIP)	5 µg	Fluoroquinolones	≤ 20/21–30/≥31	I	Highest priority critically important
Nalidixic acid (NA)	30 µg	Quinolones (1st gen)	≤ 13/14–18/≥19	I	Highest priority critically important
Erythromycin (E)	15 µg	Macrolides	≤ 13/14–22/≥23	II	Highly Important
Gentamicin (CN)	10 µg	Aminoglycoside	≤ 12/13–14/≥15	I	Critically Important
Streptomycin (S)	10 µg	Aminoglycoside	≤ 11/12–13/≥14	I	Critically Important
Amikacin (AK)	30 µg	Aminoglycoside	≤ 14/15–16/≥17	I	Critically Important
Kanamycin (K)	30 µg	Aminoglycoside	≤ 13/14–17/≥18	I	Critically Important
Apramycin (APR)	15 µg	Aminoglycoside	≤ 14/15–16/≥17	I	Critically Important
Trimethoprim/sulfamethoxazole (SXT)	25 µg	DHFR inhibitor + Sulfonamides	≤ 10/11–15/≥16	II	Highly Important

 Information regarding antimicrobial usage in the examined poultry farms was obtained directly from farm veterinarians, supervisors, and diagnostic laboratories through field visits and diagnostic submissions. The reported data reflect actual field practices as described by trusted veterinarians and poultry farm owners, rather than assumptions or general estimates.

A bacterial suspension was prepared by adjusting the turbidity of an overnight *Salmonella* culture to match the 0.5 McFarland standard. The standardized inoculum was uniformly spread over the surface of Mueller-Hinton agar (Oxoid Ltd, Hampshire, England) using a sterile cotton swab. Antimicrobial discs were aseptically placed onto the agar surface. The plates were incubated at 37 °C for 18–24 h, after which the diameters of inhibition zones were measured in millimeters. The results were interpreted based on CLSI (2024) breakpoints [25]. Multidrug-resistant (MDR) *Salmonella* isolates were identified according to the criteria established by Magiorakos et al. [26], where an isolate is considered MDR if it exhibited non-susceptibility to at least one antimicrobial agent in three or more antimicrobial categories. Additionally, extensively drug-resistant (XDR) *Salmonella* isolates were defined as those that showed resistance to all but remain susceptible to only one or two categories, while pandrug-resistant (PDR) isolates were those resistant to all antibiotics tested across all antimicrobial categories [27]. The multiple antibiotic resistance (MAR) index was calculated as the ratio between the number of antibiotics to which the isolate was resistant and the total number of antibiotics tested. Isolates with an MAR index greater than 0.2 were considered to originate from high-risk contamination sources, indicating significant antimicrobial resistance concerns.

### Detection of colistin resistance in*Salmonella*using the broth microdilution (BMD) method

The broth microdilution (BMD) technique was employed to assess the minimum inhibitory concentrations (MICs) of *Salmonella* isolates, in accordance with the standards set by the European Committee on Antimicrobial Susceptibility Testing (EUCAST) [29]. Based on EUCAST breakpoints, isolates with MIC values of ≤ 2 mg/L were considered susceptible, while those with MIC values >2 mg/L were classified as resistant. To prepare the antibiotic solution, colistin sulfate powder (Sigma, USA) was dissolved in sterile distilled water, and a series of twofold dilutions ranging from 0.25 to 16 µg/mL was prepared using cation-adjusted Mueller–Hinton broth (CAMHB) (Oxoid Ltd, Hampshire, England). *Salmonella* colonies grown for 18 h on tryptic soy agar (TSA) were collected and suspended in CAMHB. After incubation at 37 °C overnight, the bacterial suspension was standardized to the turbidity of 0.5 McFarland, then further diluted to achieve a final concentration of approximately 5 × 10⁵ CFU/mL. A total volume of 100 µL was dispensed into each well of a sterile 96-well U-bottom polystyrene microtiter plate, consisting of 50 µL of CAMHB, 25 µL of colistin solution, and 25 µL of the standardized bacterial suspension. The plates were then incubated at 37 °C for 20 h. The MIC was interpreted as the lowest colistin concentration that prevented visible bacterial growth.

### Molecular identification of genetic elements associated with antimicrobial resistance in *Salmonella*

A uniplex PCR was performed to detect key antimicrobial resistance genes in *Salmonella* isolates (*n* = 29). The targeted genes included β-lactamase resistance genes (*bla*_TEM_ [30], *bla*_SHV_ [30], *bla*_CTX−M_ [31], *bla*_VEB−1_ [32], *bla*_OXA−2_ [33], *bla*_OXA−10_ [32], and *bla*_CMY−2_ [34]), carbapenemase genes (*bla*_GES_ [35], *bla*_OXA−48_ [36], *bla*_IMP_ [36], *bla*_VIM_ [36], *bla*_KPC_ [37], and *bla*_NDM−1_ [36]), mobile colistin resistance genes (*mcr*−1 [38], *mcr-*2 [38], *mcr-*3 [38], *mcr-*4 [38], *mcr-*5 [38], *mcr-*7.1 [38], and *mcr*−9 [39]), and the integrase class 1 gene (*intI*1) [40]. *P. aeruginosa* OQ077571, OQ077572, and OQ077570 were used as control positives for the *mcr*−1, *intI*1, and *bla*_OXA−10_ genes, respectively. PCR primers and amplicon sizes are listed in Table 1. The amplified products of the *bla*_TEM_, *bla*_SHV_, *bla*_CTX−M_, *bla*_CMY−2,_*mcr*−1, and *intI*1 genes were sequenced as previously mentioned. The sequences were submitted to GenBank as illustrated in Table** 2**

### Statistical analysis

Data were statistically analyzed using GraphPad Prism version 9 (GraphPad Software, San Diego, CA, USA). The chi-square (χ²) test of independence was applied to assess associations between *Salmonella* serotypes and both resistance phenotypes (MDR, XDR) and the presence of individual antimicrobial resistance genes, with p-values < 0.05 considered statistically significant. In addition, Spearman’s rank correlation analysis was performed using binary data (presence/absence) to examine the strength and direction of co-occurrence patterns among resistance genes. Correlation coefficients were interpreted as follows: 0.00–0.19 = very weak, 0.20–0.39 = weak, 0.40–0.59 = moderate, 0.60–0.79 = strong, and 0.80–1.0 = very strong correlation. The correlation matrix was generated using Python (v3.10) with scipy, pandas, and seaborn libraries and visualized as a heatmap using seaborn v0.11.2. Additional heatmaps for other datasets were generated using Microsoft Excel 2019 and TBtools v1.098.

## Results

### Prevalence and molecular characterization of *Salmonella* isolates

**Fig. 1:**
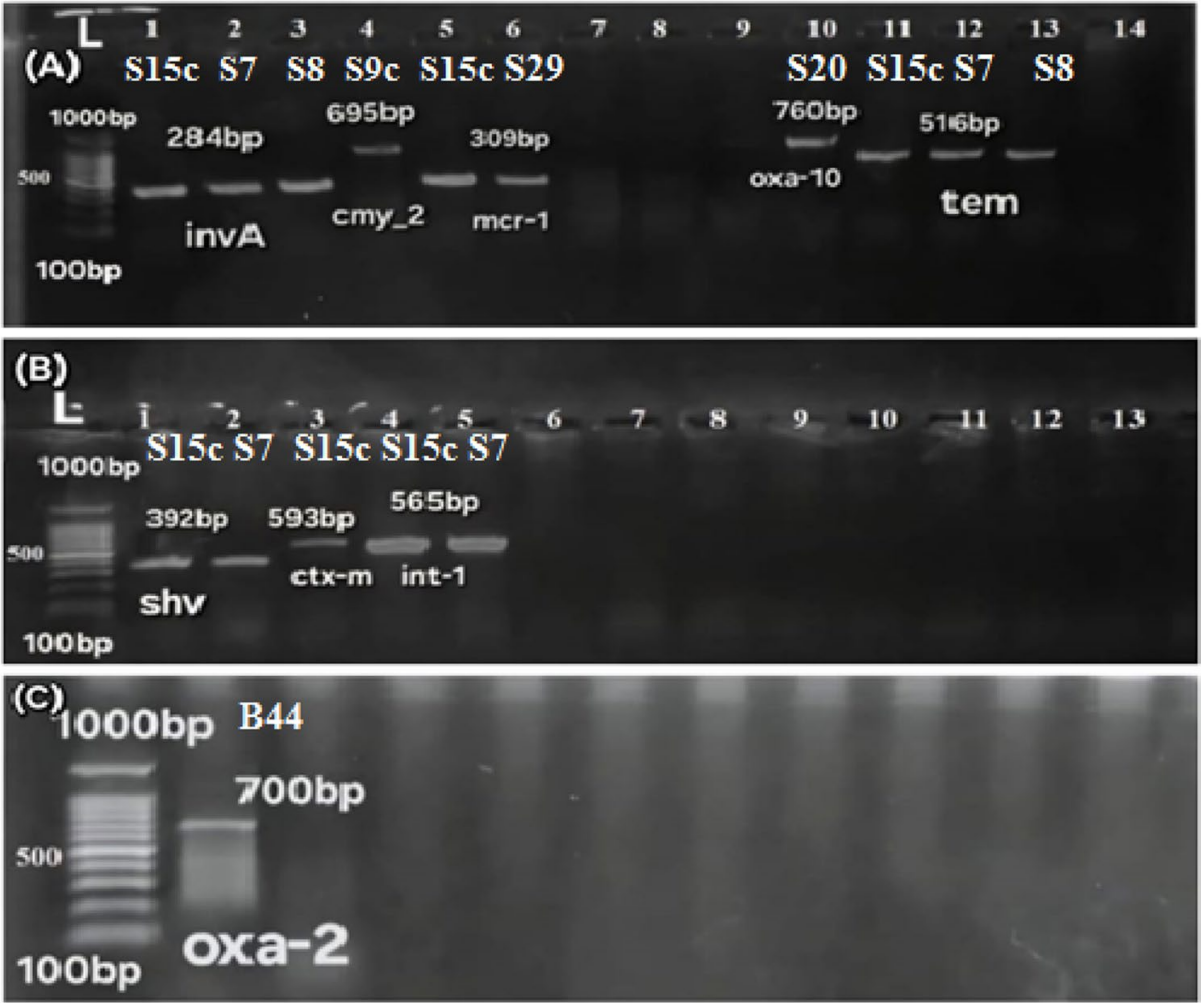
Agarose gel electrophoresis showing PCR amplification of *inv*A and antimicrobial resistance genes. **A**: lanes 1, 2, 3: *inv*A gene (284 bp), lane 4: *bla*_CMY−2_ gene (695 bp), lanes 5, 6: *mcr*−1 gene (309 bp), lane 9: *bla*_OXA−10_ gene (760 bp), lanes 11, 12, 13: *bla*_TEM_ gene (516 bp), lane 14: control negative. **B**: lanes 1, 2: *bla*_SHV_ gene (392 bp), lane 3: *bla*_CTX−M_ gene (593 bp), lanes 4, 5: *IntI*−1 gene (565 bp), lane 6: control negative. **C**: Lane 1: *bla*_OXA−2_ gene (700 bp), lane 2: control negative. (L): 1000 bp DNA ladder

 In this study, a total of 450 samples were cultured on Xylose-Lysine-Desoxycholate (XLD) agar and MacConkey’s agar, followed by aerobic incubation at 37 °C for 18 hours. Based on colony morphology, 35 isolates (7.78%) were initially suspected to be *Salmonella*, exhibiting red colonies with black centers on XLD agar (Supplementary file 2: Fig. S1)and pale, non-lactose-fermenting colonies on MacConkey’s agar. Gram staining revealed gram-negative, rod-shaped bacilli. The 35 suspected *Salmonella*isolates were subjected to a panel of biochemical tests for further confirmation. Based on the results of these biochemical tests, 29 isolates (82.9%) were confirmed as *Salmonella*. Further molecular confirmation was performed by PCR targeting the*invA* gene, which verified that all 29 isolates (100%) carried the *invA*gene (Fig. 1), with an overall prevalence of 6.44% (29/450), including 3/100 (3%) from apparently healthy birds, 16/200 (8%) from diseased birds, and 10/150 (6.7%) from freshly dead birds. Among the 29 confirmed *Salmonella*isolates, 9 (31%) were recovered from cloacal swabs, while the remaining 20 (69%) originated from organ samples (Supplementary file 2: Fig. S2).

### Serological identification of *Salmonella*isolates

**Fig. 2:**
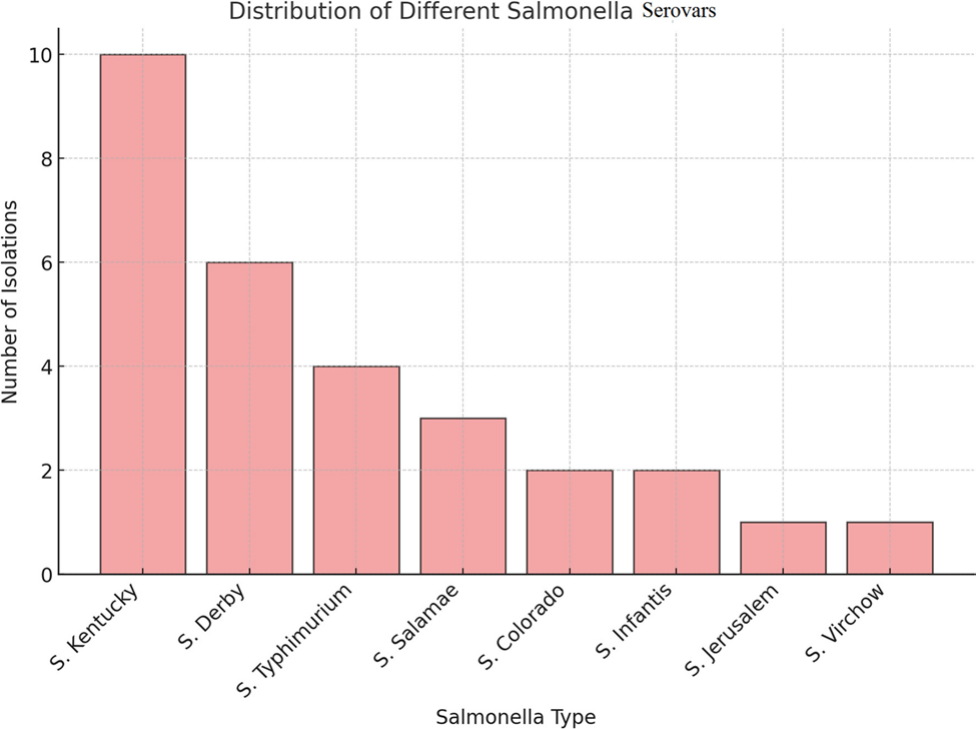
Bar chart illustrates the distribution of different *Salmonella* serotypes isolated from various farms. The x-axis represents the *Salmonella* serotypes, while the y-axis indicates the number of times each type was isolated

Confirmed *Salmonella* isolates were subjected to serological typing, which identified 8 distinct serovars among the tested *Salmonella* isolates (Table 4; Fig. 2) distributed as follows: *S.* Kentucky 10/29 (34.48%), *S.* Derby 6/29 (20.69%), *S.* Typhimurium 4/29 (13.79%), *S.* Salamae 3/29 (10.34%), *S.* Colorado 2/29 (6.90%), *S.* Infantis 2/29 (6.90%), *S.* Jerusalem 1/29 (3.45%), and *S.* Virchow 1/29 (3.45%) (Supplementary file 2: Fig. S3).

**Table 4:** Serotype distribution of *Salmonella* isolates with detailed case history

Sample ID	Serotype	Farm	Age/days	flock size	Symptoms and health issues	Mortality data
S7, S8 S9, S9c S12	*S*. Colorado 6,7:l, w:1,5 *S*. Kentucky 8,20:i: z6 *S*. Derby 1,4,[5],12:f, g:1,2	Farm 1	21–25	300,000	-Infectious Bursal Disease (IBD/Gumboro) -Infectious Bronchitis (IB)	High mortality At 16 days: 230 birds At 17 days: 222 birds At 18 days: 430 birds At 19 days: 615 birds
S15c	*S*. Jerusalem 6,7,14:z10:l, w	Farm 2	22	300,000	-IBD, IB, Chronic Respiratory Disease (CRD).	At 19 days: 29 birds At 21 days: 30 birds At 22 days: 50 birds
S18 S18c	*S*. Derby *S*. Kentucky	Farm 3	35	2,600	-IBD, IB, CRD -Newcastle (ND) -E. coli infection	High mortality rate.
S22 S23	*S*.Salamae 1,4,12,[27]: g,[m], s: [s], t:e, n,x *S*. Kentucky	Farm 4	27	16,000	-IBD, IB, CRD, ND	Mortality: 20 birds
S19, S19c S20 S20c S21, S21c	*S*. Derby *S*. Derby *S*.Typhimurium 1,4,[5],12:i:1,2 *S*.Salamae	Farm 5	31	6,000	-IBD, IB, CRD, ND -Growth Issue: Small body sizes	Mortality: 40 birds
S27	*S*. Derby	Farm 6	6	NR	-Chilled, huddling, ruffled feathers -Watery yellowish droppings, loss of appetite -Weakness and lethargy	Mortality: 6
S28, S29	*S*. Kentucky	Farm 7	1	24,000	-Chilled, huddling -Weakness and lethargy	NR
S30, S31	*S*. Kentucky	Farm 8	29	5000	-Ruffled feathers, watery yellowish droppings -Respiratory symptoms, poor growth	NR
K2	*S*.Typhimurium	Farm 9	12	NR	-Respiratory signs, ruffled feathers, watery yellowish droppings, weakness, and lethargy	NR
B12	*S*.Infantis 6,7,14:r:1,5	Farm 10	50	5000 (Sasso)	-Respiratory distress, greenish droppings -Weakness, and lethargy	NR
B17c	*S*.Infantis 6,7,14:r:1,5	Farm 11	29	NR	-CRD, sneezing, coughing, nasal discharge, gasping, swollen sinuses, watery yellowish droppings	NR
B19c	*S*.Virchow 6,7,14:r:1,2	Farm 12	NR	NR	-CRD, sneezing, nasal discharge, gasping, swollen sinuses, watery yellowish droppings	NR
B35c	S. Kentucky	Farm 13	13	NR	CRD, sneezing, nasal discharge, coughing, gasping, weakness, and poor growth -Unabsorbed yolk sac (omphalitis)	NR
B39	*S*. Kentucky	Farm 14	15	NR	CRD, sneezing, nasal discharge, coughing, gasping, weakness, and poor growth	NR
B43	*S*.Typhimurium	Farm 15	12	NR	CRD, sneezing, nasal discharge, coughing, gasping, weakness, and poor growth Unabsorbed yolk sac (omphalitis)	NR
B44	*S*.Typhimurium	Farm 16	13	NR	CRD, sneezing, nasal discharge, coughing, gasping, unabsorbed yolk sac (omphalitis)	NR

NR: not recorded due to lack of farm records. IBD: Infectious Bursal Disease. IB: Infectious Bronchitis. CRD: Chronic Respiratory Disease. ND: Newcastle Disease

### Antimicrobial susceptibility test

**Fig. 3:**
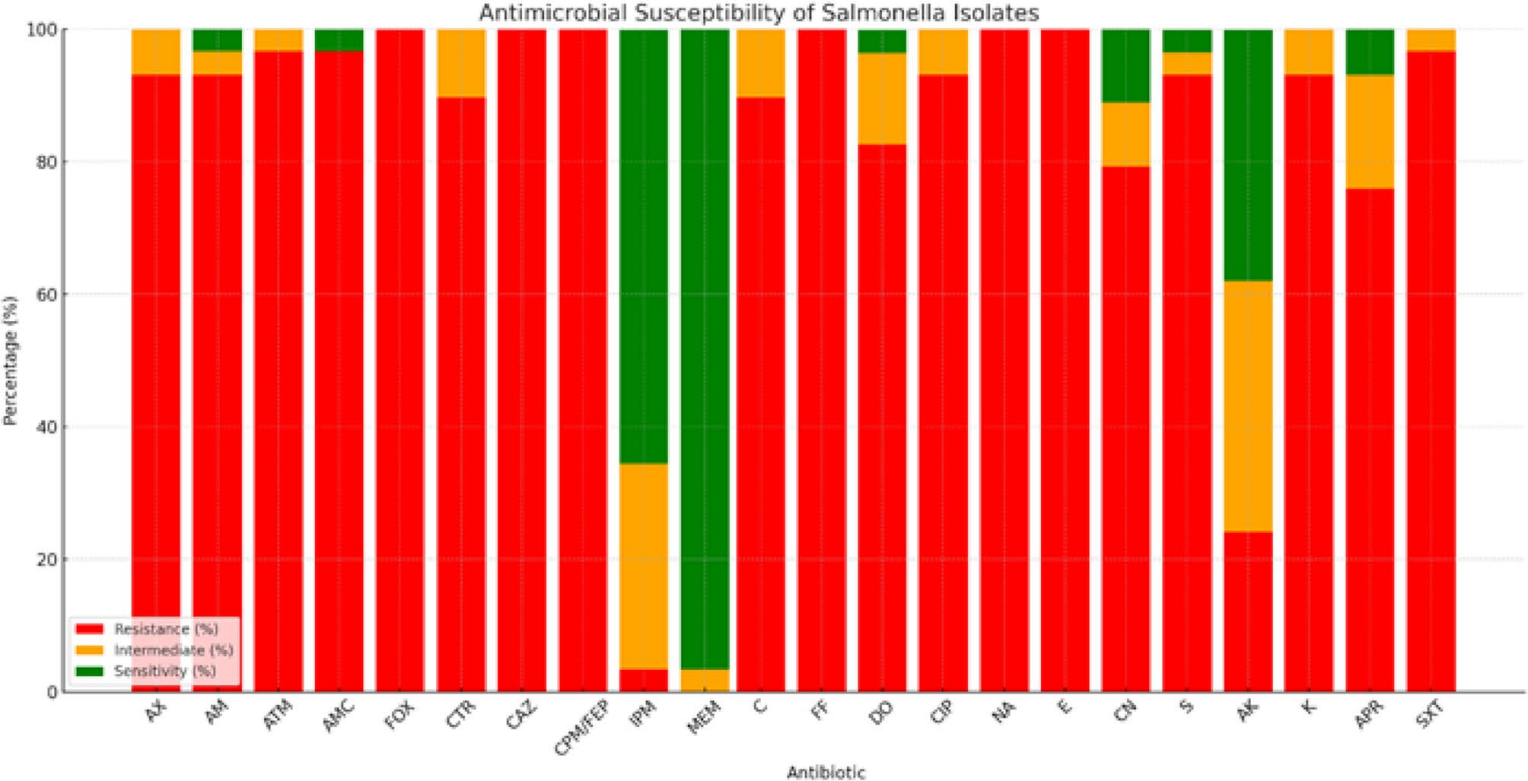
Bar chart showing the antimicrobial susceptibility results for the 29 *Salmonella* isolates. The red bars represent the percentage of resistance to each antimicrobial, while the green bars show the percentage of sensitivity

The result of the antimicrobial susceptibility test of *Salmonella* isolates (*n* = 29) revealed that all isolates exhibited complete resistance (100%) to cefoxitin (FOX, 30 µg), cefepime (CPM/FEP, 30 µg), ceftazidime (CAZ, 30 µg), nalidixic acid (NA, 30 µg), erythromycin (E, 15 µg), and fosfomycin (FF, 30 µg). High resistance was also observed against amoxicillin)AX, 25 µg) (93.1%), ampicillin (AM, 10 µg) (93.1%), aztreonam (ATM, 10 µg) (96.6%), amoxicillin-clavulanic acid (AMC, 30 µg) (96.6%), ceftriaxone (CTR, 30 µg) (89.7%), chloramphenicol (C, 30 µg) (89.7%), ciprofloxacin (CIP, 5 µg) (93.1%), gentamicin (CN, 10 µg) (79.3%), streptomycin (S, 10 µg) (93.1%), kanamycin (K, 30 µg) (93.1%), apramycin (APR, 15 µg) (75.9%), doxycycline (DO, 30 µg) (82.6%), and trimethoprim/sulfamethoxazole (SXT, 25 µg) (96.6%). Intermediate resistance was observed with amikacin (AK, 30 µg) (37.9%). In contrast, high sensitivity was observed to meropenem (MEM, 10 µg) (96.6%) and imipenem (IPM, 10 µg) (65.5%) (Table 5; Fig. 3) (Supplementary file 2: Fig. S4, 5).

**Table 5:** Antimicrobial susceptibility results of *Salmonella* isolates

Antimicrobial agents	Family	Disc code	CPD	Salmonella					
				Resistance		Intermediate		Sensitive	
				No	%	No	%	No	%
Amoxicillin	β-lactams (Aminopenicillins)	AX	25 µg	27	93.1	2	6.9	0	0.0
Ampicillin		AM	10 µg	27	93.1	1	3.4	1	0.0
Aztreonam	β-lactams (Monobactam)	ATM	10 µg	28	96.6	1	3.4	0	0.0
Amoxicillin-clavulanic	Penicillin + β-lactamase inhibitor	AMC	30 µg	28	96.6	0	0.0	1	3.4
Cefoxitin	Cephalosporin	FOX	30 µg	29	100	0	0.0	0	0.0
Ceftriaxone		CTR	30 µg	26	89.7	3	10.3	0	0.0
Ceftazidime		CAZ	30 µg	29	100	0	0.0	0	0.0
Cefepime		CPM/FEP	30 µg	29	100	0	0.0	0	0.0
Imipenem	Carbapenems	IPM	10 µg	1	3.4	9	31	19	65.5
Meropenem		MEM	10 µg	0	0.0	1	3.4	28	96.6
Chloramphenicol	Phenicol	C	30 µg	26	89.7	3	10.3	0	0.0
Fosfomycin	Phosphonic acid derivatives	FF	30 µg	29	100	0	0.0	0	0.0
Doxycycline	Tetracyclines	DO	30 µg	24	82.6	4	13.8	1	3.4
Ciprofloxacin	Fluoroquinolones	CIP	5 µg	27	93.1	2	6.9	0	0.0
Nalidixic acid	Quinolones (1st gen)	NA	30 µg	29	100	0	0.0	0	0.0
Erythromycin	Macrolides	E	15 µg	29	100	0	0.0	0	0.0
Gentamicin	Aminoglycoside	CN	10 µg	23	79.3	2	9.6	4	13.8
Streptomycin		S	10 µg	27	93.1	1	3.4	1	3.4
Amikacin		AK	30 µg	7	24.1	11	37.9	11	37.9
Kanamycin		K	30 µg	27	93.1	2	9.6	0	0.0
Apramycin		APR	15 µg	22	75.9	5	17.2	2	6.9
DHFR inhibitor + Sulfonamides	DHFR inhibitor + Sulfonamides	SXT	25 µg	28	96.6	1	3.4	0	0.0

The antimicrobial resistance patterns of *Salmonella* isolates were evaluated in relation to the World Health Organization (WHO) classification of antimicrobials according to their importance to human medicine. All isolates exhibited complete resistance to several antimicrobials categorized as highest priority critically important antimicrobials (HPCIAs), including cefepime, ceftazidime, nalidixic acid, and fosfomycin. Complete resistance was also observed to highly important antibiotics, including cefoxitin and erythromycin. Furthermore, high resistance rates were recorded against additional HPCIAs: ceftriaxone and ciprofloxacin, along with critically important antibiotics such as aztreonam, apramycin, gentamicin, streptomycin, and kanamycin, and the highly important drugs amoxicillin, ampicillin, amoxicillin-clavulanic acid, chloramphenicol, doxycycline, and trimethoprim/sulfamethoxazole. Moderate resistance was observed to the critically important antibiotic amikacin.

Based on the antibiogram profiles of the tested *Salmonella* isolates, resistance types were categorized into multidrug-resistant (MDR), extensively drug-resistant (XDR), and pandrug-resistant (PDR) according to internationally recognized definitions. Notably, extensively drug-resistant (XDR) phenotypes were identified in 22 isolates (75.49%), each exhibiting resistance to at least 9 out of 11 antimicrobial categories tested and remaining susceptible to only one or two categories. In addition, seven isolates (24.1%) were classified as multidrug-resistant (MDR), showing resistance to at least one agent in three or more antimicrobial classes. A total of 22 distinct antimicrobial resistance profiles were identified among the 29 *Salmonella* isolates. The most frequent resistance profile (*n* = 4) included AX, AM, ATM, AMC, FOX, CTR, CAZ, CPM\FEP, C, FF, DO, CIP, NA, E, CN, S, K, APR, and SXT. The MAR index among isolates ranged from 0.6 to 0.9 (Table 6) (Supplementary file 2: Table S2).

**Table 6:** Resistance profiles and antibiotyping of *Salmonella* isolates

Antibiotypes	Resistance pattern	Isolates, no.	No. of resistant Antibiotic	MDRINDEX	Type of resistance
I	AX, AM, ATM, AMC, FOX, CTR, CAZ, CPM\FEP, IPM, C, FF, DO, CIP, NA, E, CN, S, K, APR, SXT	1	20/22	0.9	XDR
II	AX, AM, ATM, AMC, FOX, CTR, CAZ, CPM\FEP, C, FF, DO, CIP, NA, E, CN, S, AK, K, APR, SXT	2	20/22	0.9	XDR
III	AX, AM, ATM, AMC, FOX, CTR, CAZ, CPM\FEP, C, FF, DO, CIP, NA, E, CN, S, AK, K, APR, SXT	1	20/22	0.9	XDR
IV	AX, AM, ATM, AMC, FOX, CTR, CAZ, CPM\FEP, C, FF, CIP, NA, E, CN, S, AK, K, APR, SXT	1	19/22	0.9	XDR
V	AX, AM, ATM, AMC, FOX, CTR, CAZ, CPM\FEP, C, FF, DO, CIP, NA, E, CN, S, K, APR, SXT	4	19/22	0.9	XDR
VI	AX, AM, ATM, AMC, FOX, CTR, CAZ, CPM\FEP, FF, DO, CIP, NA, E, CN, S, AK, K, APR, SXT	1	19/22	0.**9**	MDR
VII	AX, AM, ATM, AMC, FOX, CTR, CAZ, CPM\FEP, C, FF, DO, CIP, NA, E, CN, S, AK, K, SXT	1	19/22	0.**9**	XDR
VIII	AX, AM, ATM, AMC, FOX, CTR, CAZ, CPM\FEP, C, FF, DO, CIP, NA, E, CN, S, K, APR, SXT	3	19/22	0.**9**	XDR
IX	AX, AM, ATM, AMC, FOX, CTR, CAZ, CPM\FEP, C, FF, DO, CIP, NA, E, CN, S, K, APR, SXT	1	19/22	0.**9**	XDR
X	AX, AM, ATM, AMC, FOX, CTR, CAZ, CPM\FEP, C, FF, DO, CIP, NA, E, CN, S, K, SXT	2	18/2**2**	0.8	XDR
XI	AX, AM, ATM, AMC, FOX, CTR, CAZ, CPM\FEP, C, FF, DO, CIP, NA, E, S, K, APR, SXT	1	18/22	0.8	XDR
XII	AX, AM, ATM, AMC, FOX, CTR, CAZ, C, FF, DO, CIP, NA, E, S, K, APR, SXT	1	17/22	0.8	XDR
XIII	AM, ATM, AMC, FOX, CTR, CAZ, CPM\FEP, C, FF, CIP, NA, E, CN, S, AK, APR, SXT	1	17/22	0.8	XDR
XIV	AX, AM, ATM, AMC, FOX, CTR, CAZ, CPM\FEP, C, FF, DO, CIP, NA, E, S, K, SXT	1	17/22	0.8	XDR
XV	AX, AM, ATM, AMC, FOX, CTR, CAZ, CPM\FEP, C, FF, DO, CIP, NA, E, K, APR, SXT	1	17/22	0.**8**	XDR
XVI	AX, ATM, AMC, FOX, CTR, CAZ, CPM\FEP, FF, DO, CIP, NA, E, CN, S, K, APR, SXT	1	17/22	0.**8**	MDR
XVII	AX, AM, ATM, AMC, FOX, CAZ, CPM\FEP, C, FF, CIP, NA, E, CN, S, K, APR, SXT	1	17/22	0.**8**	MDR
XVIII	AX, AM, ATM, AMC, FOX, CTR, CAZ, CPM\FEP, C, FF, CIP, NA, E, CN, S, APR	1	16/22	0.7	MDR
XIX	ATM, AMC, FOX, CAZ, CPM\FEP, C, FF, DO, CIP, NA, E, CN, S, K, APR, SXT	1	16/22	0.7	XDR
XX	AX, AM, ATM, AMC, FOX, CTR, CAZ, CPM\FEP, C, FF, CIP, NA, E, S, K, SXT	1	16/22	0.7	MDR
XXI	AX, AM, ATM, AMC, FOX, CTR, CAZ, CPM\FEP, FF, DO, NA, E, CN, K, SXT	1	15\22	0.7	MDR
XXII	AX, AM, ATM, AMC, FOX, CTR, CAZ, CPM\FEP, FF, DO, NA, E, K, SXT	1	14/22	0.6	MDR

To evaluate whether antimicrobial resistance patterns varied significantly among the different *Salmonella* serotypes, a chi-square test of independence was performed. The test assessed the association between the identified serotypes and their corresponding resistance profiles (MDR and XDR). The analysis revealed no statistically significant association between the *Salmonella* serotype and resistance type (χ² = 6.61, df = 7, *p* = 0.471) (*p* > 0.05). This indicates that the distribution of resistance categories (MDR and XDR) was not dependent on the serotype in this study. Although certain serotypes, such as *S*. Kentucky and *S*. Derby, appeared to exhibit a higher frequency of XDR phenotypes, this trend was not statistically significant.

**Fig. 4:**
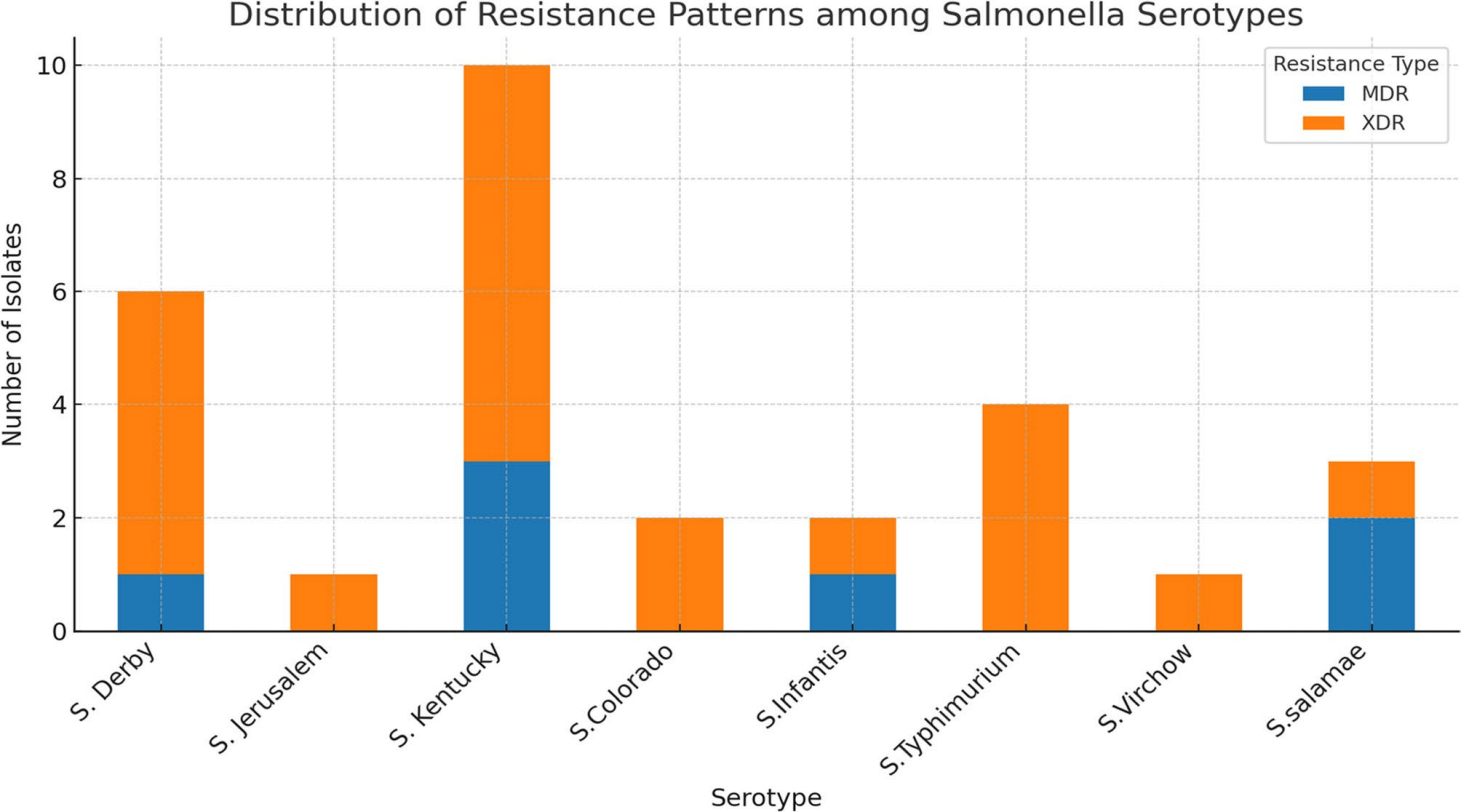
Bar chart illustrating the distribution of antimicrobial resistance patterns among *Salmonella* serotypes, showing a predominance of extensively drug-resistant (XDR) strains across multiple serotypes, with relatively fewer multidrug-resistant (MDR) strains

The distribution of phenotypic resistance types among the investigated *Salmonella* serotypes is shown in Fig. 4. Overall, XDR patterns predominated across most serotypes, with *S*. Kentucky, *S*. Derby, and *S*. Typhimurium exhibiting the highest frequencies of XDR profiles. MDR phenotypes were less common and mainly observed in *S*. Salamae, *S*. Virchow, and selected isolates of *S*. Derby and *S*. Infantis. This trend highlights the alarming prevalence of extensively drug-resistant strains, particularly among the most frequently detected serotypes.

### Determination of colistin resistance among *Salmonella* isolates using the broth microdilution method

The minimum inhibitory concentrations (MICs) of colistin for *Salmonella* isolates were determined using the broth microdilution (BMD) method, following the guidelines of EUCAST. According to EUCAST breakpoints, isolates with MIC values ≤ 2 mg/L were interpreted as susceptible, whereas those with MIC values > 2 mg/L were classified as resistant. In this study, out of the 29 tested *Salmonella* isolates, 8 isolates (27.6%) demonstrated resistance to colistin, with MIC values exceeding the 2 mg/L threshold, while 21 isolates (72.4%) were sensitive.

### Distribution of antimicrobial resistance genes among* Salmonella* isolates

**Fig. 5:**
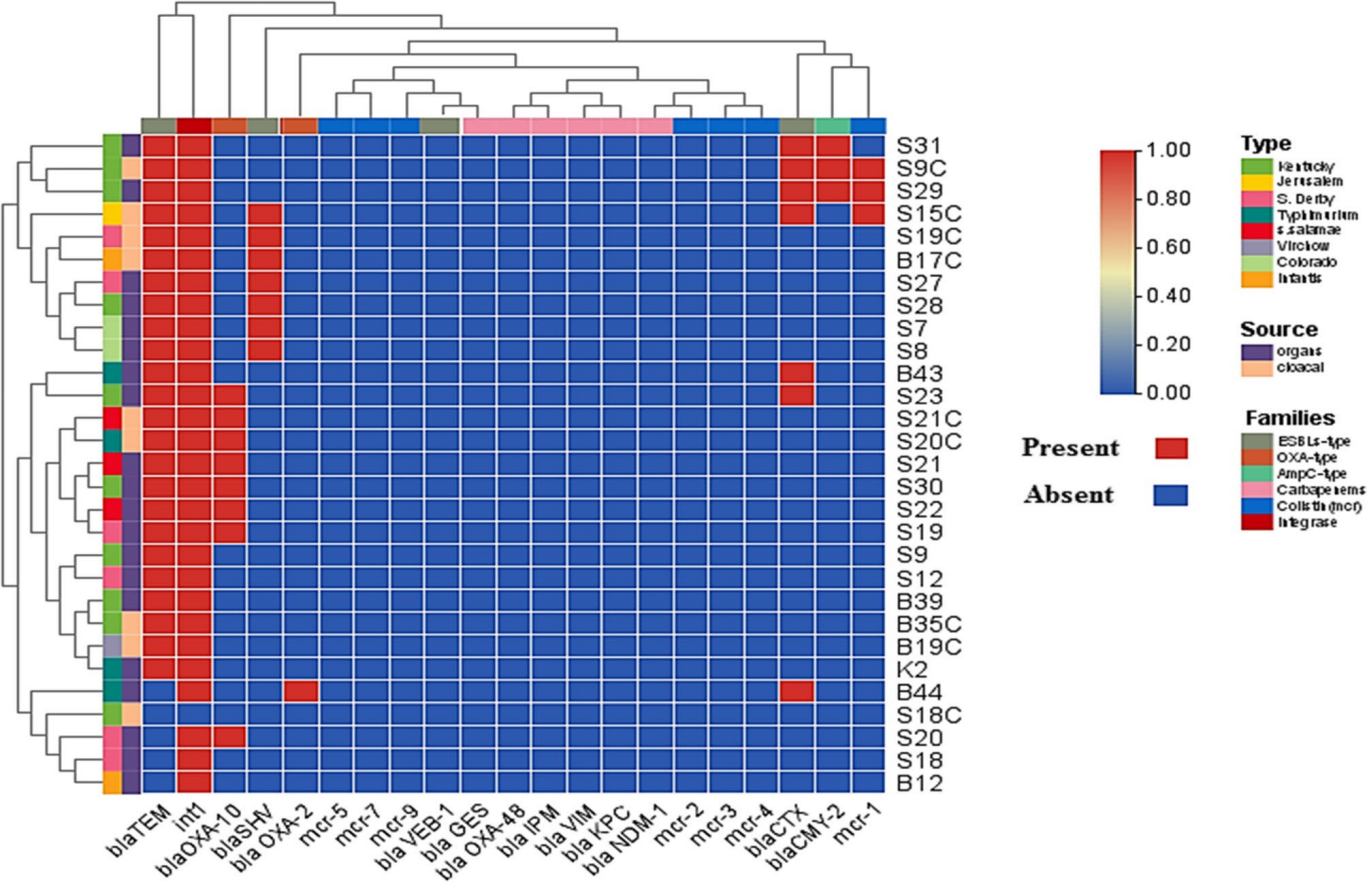
Heatmap illustrates the distribution of antibiotic resistance genes (ARGs). The color gradient indicates the presence or absence of specific resistance genes, with red indicating presence and blue indicating absence. The hierarchical clustering at the top and left side groups similar samples and genes based on their expression patterns. The families of resistance genes are color-coded on the top. The source of isolates (organs vs. cloacal samples) and serotyping are color-coded on the left

In this study, the distribution of antimicrobial resistance genes among 29 *Salmonella* isolates was investigated. The most prevalent β-lactamase gene was *bla*_TEM_ (82.8%), followed by *bla*_OXA−10_ (27.6%), *bla*_SHV_ (24.1%), and *bla*_CTX−M_ (24.1%). Other β-lactamase genes were detected at lower frequencies, including *bla*_CMY−2_ (10.3%) and *bla*_OXA−2_ (3.4%), while *bla*_VEB−1_ was not detected. Carbapenemase genes, including *bla*_GES_, *bla*_OXA−48_, *bla*_IPM_, *bla*_VIM_, *bla*_KPC_, and *bla*_NDM−1_, were not detected in any of the isolates. Regarding colistin resistance, only *mcr*−1 was identified (10.3%), while *mcr*−2, *mcr*−3, *mcr*−4, *mcr*−5, *mcr*−7.1, and *mcr*−9 were absent in all isolates. The class 1 integron gene *intI*1 showed the highest prevalence, being present in 96.6% of isolates (Fig. 1, 5) (Supplementary file 2: Fig. S6, S7, S8, S9). A chi-square analysis was performed to assess the association between *Salmonella* serotypes and the presence of the antimicrobial resistance genes *bla*_TEM_, *bla*_OXA−10,_*bla*_SHV_, *bla*_CTX−M_, *bla*_CMY−2_, *bla*_OXA−2,_*mcr*−1, and *intI*1. The results revealed no statistically significant differences in the distribution of these genes among the serotypes (*p* > 0.05), indicating that the presence of resistance genes was not significantly associated with specific serotypes. For example, *bla*_TEM_ and *intI*1 were widely distributed across nearly all serotypes. Although *bla*_CTX−M_ appeared to cluster in specific serotypes such as *S*. Kentucky and *S*. Jerusalem, the overall number of positive isolates was limited, which may have reduced the statistical power of the chi-square test. Consequently, no significant association was detected (*p* > 0.05).

Notably, the *mcr*−1 gene was identified specifically in *S.* Kentucky (*n* = 2) and *S*. Jerusalem (*n* = 1). A Chi-square test showed no statistically significant association between *mcr*−1 carriage and serotype (χ² = 2.14, df = 3, *p* = 0.542). Similarly, *bla*_SHV_ with no significant serotype correlation (χ² = 4.32, df = 3, *p* = 0.229). The *bla*_OXA−10_ gene was detected across various serotypes without statistical significance (χ² = 2.31, df = 2, *p* = 0.315). The *bla*_CMY−2_ gene, although exclusively found in *S*. Kentucky (2/29, 6.9%), did not show a statistically significant association (χ² = 2.72, df = 1, *p* = 0.099), possibly due to the small number of positive isolates. Finally, *bla*_OXA−2_ was detected in only one isolate (3.4%), specifically in *S*. Typhimurium. Due to the limited number of positive isolates, statistical analysis could not be performed. Nevertheless, its exclusive detection in this serotype may warrant further investigation in larger sample sets to determine any potential association.

**Fig. 6:**
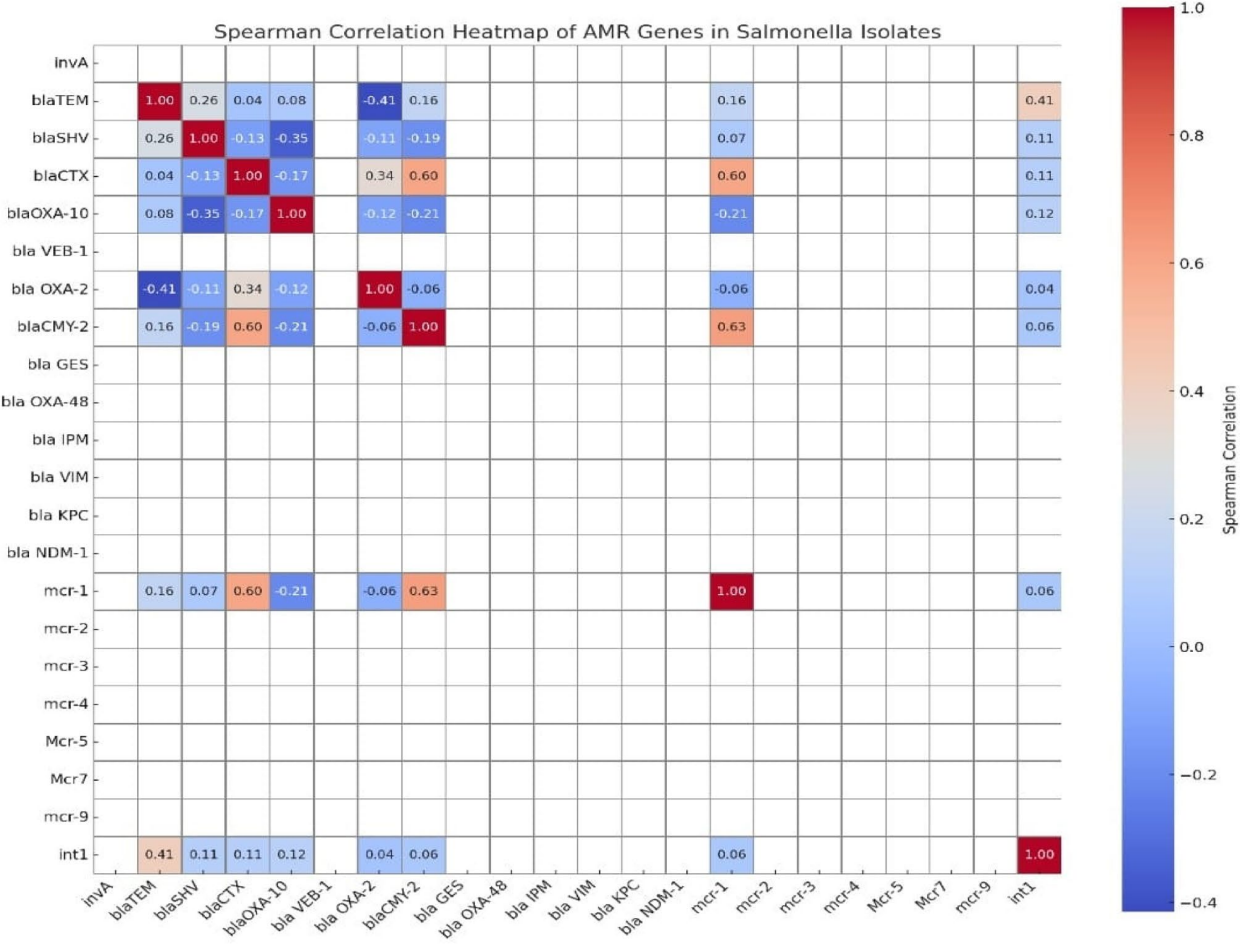
Spearman correlation heatmap showing the co-occurrence patterns among antimicrobial resistance genes

To explore the co-occurrence patterns of antimicrobial resistance (AMR) genes among *Salmonella* isolates, a Spearman correlation analysis was conducted using binary presence/absence data. Spearman correlation analysis revealed several positive associations between antimicrobial resistance genes among *Salmonella* isolates (Fig. 6). A moderate correlation was detected between *bla*_TEM_ and *intI*1 (ρ = 0.41). *Bla*_CTX−M_ showed strong correlations with *mcr*−1 (ρ = 0.60) and *bla*_CMY−2_ (ρ = 0.60), while *bla*_OXA−2_ was moderately correlated with *bla*_CTX−M_ (ρ = 0.34). *Bla*_CMY−2_ also exhibited a strong correlation with *mcr*−1 (ρ = 0.63). The *mcr*−1 was the most prevalent colistin resistance gene and showed multiple associations with β-lactamase genes. Other genes, including *bla*_VEB−1_, *bla*_GES_, *bla*_OXA−48_, *bla*_IPM_, *bla*_VIM_, *bla*_KPC_, *bla*_NDM−1_, and other *mcr* variants (*mcr*−2, *mcr*−3, *mcr*−4, *mcr*−5, *mcr*−7.1, and *mcr*−9), displayed no significant correlations.

### Phylogenetic analysis of virulence and antibiotic resistance genes in *Salmonella *strains Phylogenetic analysis of the *invA* gene in *Salmonella enterica *strains

**Fig. 7:**
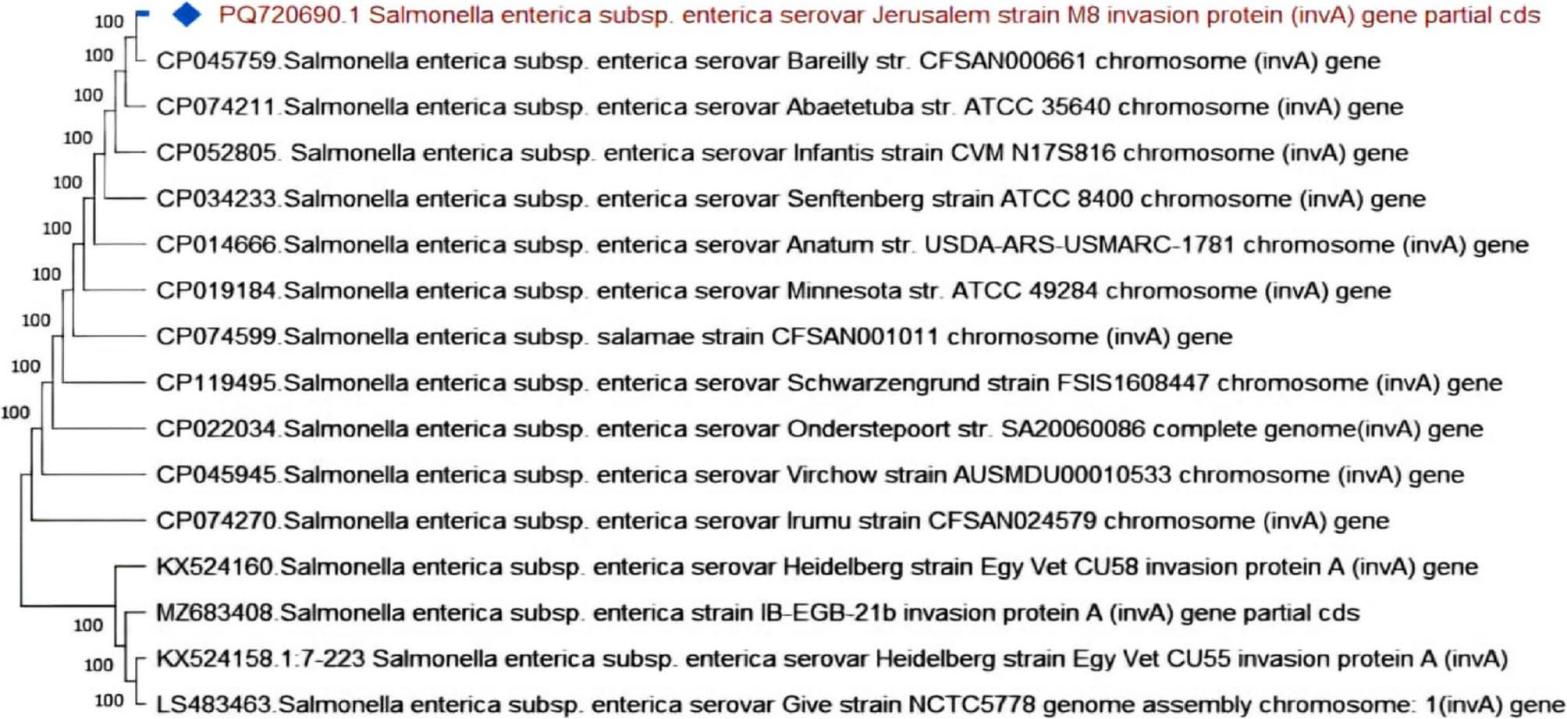
Phylogenetic tree represents the genetic relationship of the invasion protein (*inv*A) gene among different *Salmonella* strains. The strain reported in this study is marked with a diamond symbol. The *inv*A gene in *Salmonella* Jerusalem strain M8 shares 100% homology with various *Salmonella* serovars

**Fig. 8:**
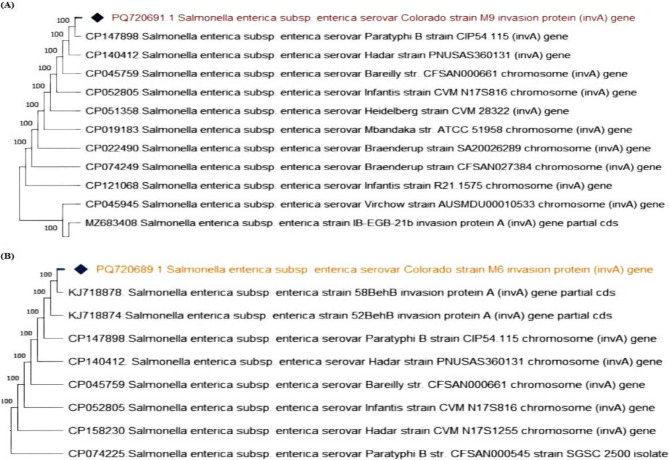
(**A**): The *inv*A gene of *Salmonella enterica* Colorado strain M9 exhibits 100% similarity to other serovars. **B**: The phylogenetic tree shows 100% homology of the *inv*A gene in *Salmonella enterica* serovar Colorado strain M6, clustering closely with other *Salmonella* serovars

The phylogenetic analysis of the invasion protein (*inv*A) gene revealed a high degree of genetic conservation among various serovars of *Salmonella enterica subsp. enterica*. The strains examined in this study, *S.* Jerusalem strain M8, *S.* Colorado strain M9, and *S.* Colorado strain M6, clustered closely with other serovars, supported by a strong bootstrap value of 100%. These findings are indicative of robust phylogenetic relationships and highlight the widespread conservation of this virulence gene across diverse serovars (Fig. 7, 8).

### Phylogenetic analysis of β-lactamase resistance genes (*bla*_SHV_, *bla *_TEM_,*bla*_CTX−M,_ and *bla *_CMY−2_)

**Fig. 9:**
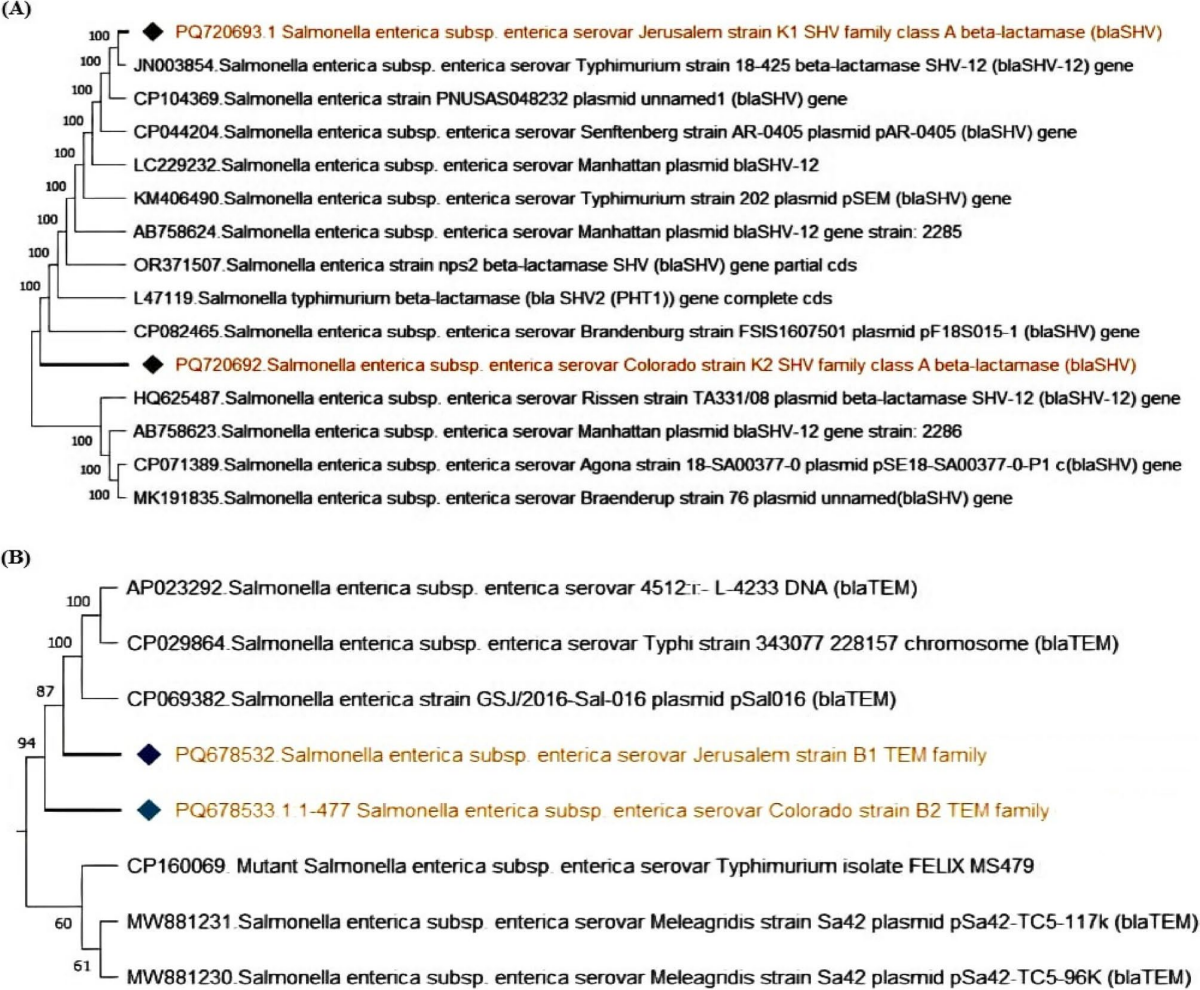
Phylogenetic analysis of *bla*_SHV_ and *bla*_TEM_. The strains presented in this study are labeled with diamond symbols. **A**: The *bla*_SHV_ gene in *Salmonella enterica* Jerusalem strain K1 and Colorado strain K2 shows 100% homology with SHV-producing strains. **B**: The *bla*_TEM_ gene in *Salmonella enterica* Jerusalem strain B1 and Colorado strain B2 clusters closely with other resistant strains

**Fig. 10:**
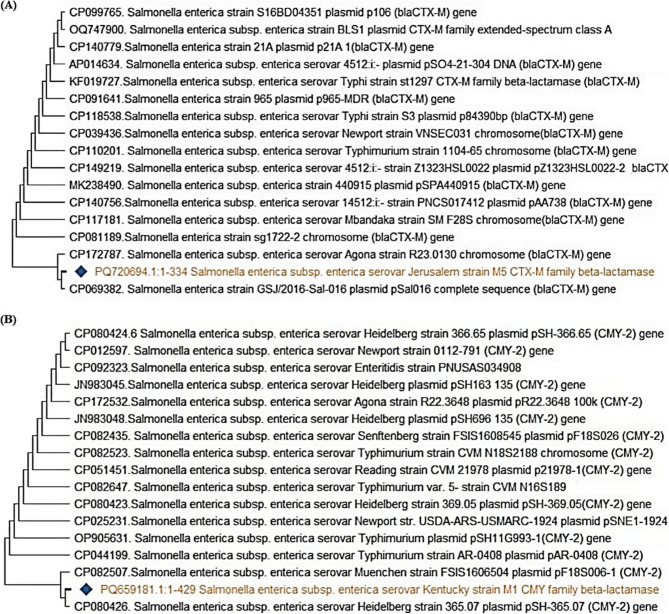
The phylogenetic tree illustrates the genetic relatedness of the *bla*_CTX−M_ and *bla*_CMY−2_ β-lactamase genes among different *Salmonella* serovars. The strain reported in this study is marked with a diamond symbol. **A**: *Salmonella enterica* Jerusalem shares significant genetic similarity with various plasmid-borne *bla*_CTX−M_ genes from *Salmonella* strains. **B**: The highlighted strain, *S*. Kentucky M1, carries *bla*_CMY−2_ gene, clustering with other resistant isolates

The phylogenetic trees for beta-lactamase genes demonstrated a high degree of similarity among *Salmonella enterica* strains, suggesting global widespread dissemination of these resistance determinants and emphasizing the importance of monitoring beta-lactamase-mediated resistance in foodborne pathogens. The *bla*_SHV_ gene in *Salmonella enterica* Jerusalem and Colorado displayed 100% homology with SHV-producing strains such as *S*.Typhimurium, *S*. Senftenberg, and *S.* Manhattan, indicating a common genetic origin or horizontal gene transfer and the widespread presence of this resistance gene in different *Salmonella* serovars (Fig. 9A). The *bla*_TEM_ gene in *S.* Jerusalem and *S*. Colorado clustered closely with resistant strains of *S*. Typhimurium and *S.* Meleagridis, underscoring the role of *bla*_TEM_ in multidrug resistance (Fig. 9B). The *bla*_CTX−M_ gene in *S.* Jerusalem exhibited significant similarity to plasmid-borne *bla*_CTX−M_ genes from *S.* Typhimurium, *S*. Anatum, and *S.* Heidelberg, suggesting plasmid-mediated dissemination of resistance (Fig. 10A). The *bla*_CMY−2_ gene in *S*. Kentucky strain M1 clustered with resistant isolates of *S.* Heidelberg, *S*. Newport, and *S.* Typhimurium, highlighting its role in cephalosporin resistance (Fig. 10B).

### Phylogenetic analysis of *mcr−*1 gene

**Fig. 11:**
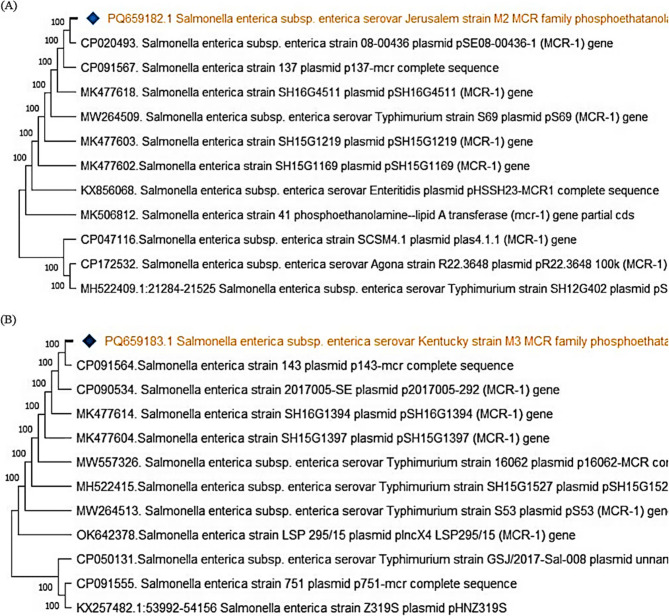
Phylogenetic tree of *mcr*−1 gene among different *Salmonella* strains. The strain reported in this study is marked with a diamond symbol. **A**: *Salmonella enterica* Jerusalem shares high genetic similarity (100%) with multiple *Salmonella* strains carrying the *mcr*−1 gene. **B**: *Salmonella enterica* Kentucky Strain M3 exhibits 100% similarity with other *Salmonella* strains carrying plasmid-borne *mcr*−1 genes

The *mcr*−1 gene, associated with colistin resistance, was identified in *S*. Jerusalem and *S*. Kentucky strain M3, showing 100% genetic similarity to *mcr*−1-carrying strains of *S*. Typhimurium and *S.* Enteritidis, suggesting horizontal gene transfer (Fig. 11).

### Phylogenetic analysis of class 1 integron (*intI*1)

**Fig. 12:**
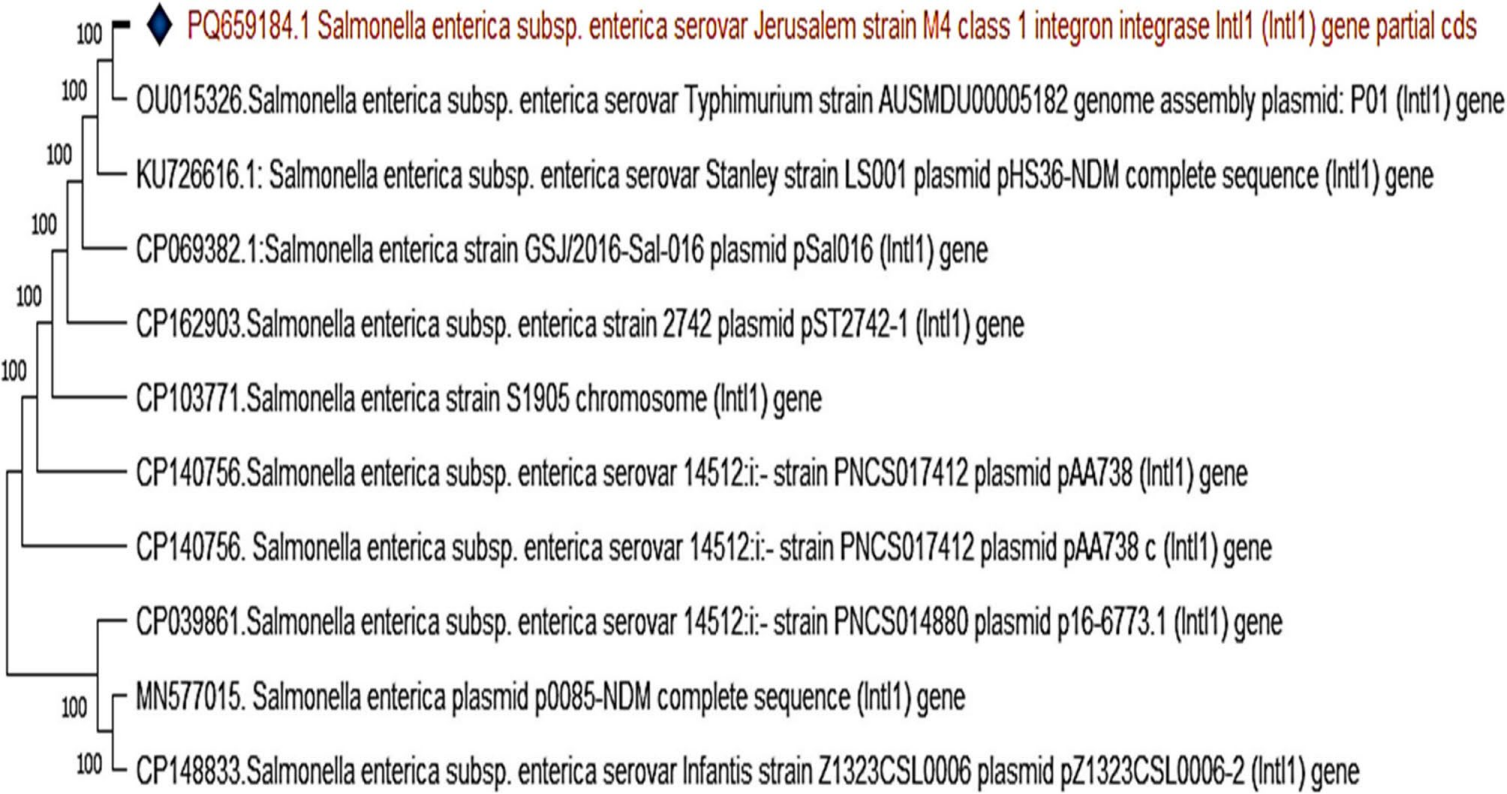
The phylogenetic tree illustrates the genetic relationship of the intI1 gene among different Salmonella enterica strains. The highlighted strain, Salmonella enterica serovar Jerusalem, shows a 100% homology with other strains

The *intI*1 gene in *S*. Jerusalem exhibited 100% homology with strains of *S.* Typhimurium, *S*. Stanley, and *S*. Infantis, emphasizing its role in resistance gene dissemination (Fig. 12).

## Discussion

The phylogenetic tree illustrates the genetic relationship of the *intI*1 gene among different *Salmonella enterica* strains. The highlighted strain, *Salmonella enterica* serovar Jerusalem, shows a 100% homology with other strains *Salmonella* is a major foodborne pathogen that represents a significant public health concern. In poultry, infections are associated with reduced productivity, increased mortality, and considerable economic losses. The pathogen’s ability to persist in the environment, colonize birds without clinical signs, and survive through the food processing chain contributes to its epidemiological success [7]. This underscores the importance of assessing its prevalence in poultry production systems.

 In this study, 29 isolates (6.4%) were recovered from 450 poultry samples from various broiler farms and confirmed as *Salmonella enterica* through detection of the *inv*A gene. This prevalence aligns with several Egyptian studies, which reported comparable rates between 6.0% and 6.9% in broiler farms and poultry carcasses [41–45], indicating a consistent moderate presence of *Salmonella* in Egypt’s poultry sector. Other investigations documented higher rates, such as Abd El-Mohsen et al. [46] (11.36%) and Tawfik et al. [47] (8%), whereas Saad et al. [48] (5.3%) and Shalaby et al. [49] (2.28%) reported lower levels. Internationally, similar prevalence has been documented, including 6.4% in Ethiopia [50], 6.4% in Nigeria [51], and 5.66% in China [52]. The observed differences among studies could be attributed to variations in sample sources, collection methods, farm management practices, biosecurity measures, poultry type (broilers vs. layers), and production systems (open vs. closed). Additional contributing factors include geographical location, antibiotic usage, water quality, and handling during processing [53, 54]. These variations emphasize the need for further investigations into the role of farming practices, biosecurity, and environmental conditions. Such research is essential for designing targeted control strategies and implementing effective measures to limit *Salmonella* dissemination in poultry production.

Accurate diagnosis and epidemiological tracking of *Salmonella* rely on laboratory-based approaches, particularly bacterial isolation followed by serotyping. These methods provide essential information on circulating serovars, which is indispensable for outbreak investigations, source attribution, and the development of sustainable prevention measures [55]. Notably, the rare serovars *Salmonella enterica* ser. Jerusalem and ser. Colorado were detected in poultry samples from Egypt. To our knowledge, this is the first report of these serovars in the Egyptian poultry sector. Nevertheless, further genomic analyses (e.g., WGS, cgMLST) are required to confirm their novelty and phylogenetic relationships. Globally, reports of *S*. Jerusalem are scarce, with occasional outbreaks linked to organic soya feed in poultry farms in Switzerland and Italy [56]. Similarly, *S*. Colorado is an uncommon serotype, first described by Edwards and Hermann (1949) from human and animal cases [57], with only sporadic reports since then. The detection of these rare serovars in Egyptian poultry raises important epidemiological questions about their possible origins, which may include introduction via breeder flocks, contaminated feed, or cross-border trade. Their presence may also indicate previously undetected endemic circulation.

Another noteworthy finding was the isolation of *S*. Derby, a serotype primarily associated with swine and pork products worldwide [58] and not commonly linked to poultry. Although infrequently detected in avian hosts, it was identified in this study from chicken samples. In Egypt, only a few reports have documented its occurrence in poultry, including ducks [59], table eggs [60], and a small proportion of broiler chickens [61]. Its detection in the present study indicates an atypical occurrence in poultry hosts and further underscores the serotype diversity within Egypt’s poultry production systems. In addition to these serovars, several common types were also identified, including *S*. Kentucky, *S*. Typhimurium, *S*. Salamae, *S*. Infantis, and *S*. Virchow, which are consistent with earlier Egyptian reports [62–65]. Taken together, these findings demonstrate the wide diversity of *Salmonella enterica* serotypes circulating in poultry, ranging from frequently detected types to rare serovars. This highlights the urgent need for enhanced, integrated surveillance systems combining serotyping and molecular tools to monitor serovar distribution and zoonotic potential. Such measures are critical for developing effective surveillance and intervention programs, ensuring food safety, and safeguarding both poultry production and public health.

 In Egypt, there is no legislation regulating the use of antibiotics in poultry farms. A wide range of antimicrobials, including colistin, erythromycin, tetracycline, kanamycin, streptomycin, gentamicin, quinolones, ciprofloxacin, and beta-lactams, are frequently applied for both therapeutic and prophylactic purposes. Such improper and unregulated usage facilitates the rapid selection of multidrug resistant Enterobacteriaceae in poultry and promotes the transmission of resistant bacteria along the food chain to humans [66–68]. In this study, antimicrobial susceptibility testing of *Salmonella* isolates (*n* = 29) revealed a high level of MDR, consistent with earlier reports from Egypt. For example, Awad et al. [63] documented high resistance to erythromycin (96.78%), trimethoprim-sulfamethoxazole (93.55%), doxycycline (93.55%), streptomycin (80.65%), and amoxicillin (67.8%), which are in close agreement with our findings. A notable exception was gentamicin: while Awad et al. [63] reported a high susceptibility rate (96.77%), our study found only 13.8% susceptibility, indicating a marked decline in its efficacy and raising concerns about its continued therapeutic value. Similarly, Youssef et al. [69] reported complete resistance to amoxicillin and erythromycin, as well as high resistance to ampicillin (98%) and doxycycline (94%). However, their isolates remained more susceptible to ciprofloxacin (80%) compared to our isolates, which exhibited 93.1% resistance, suggesting a worrying increase in fluoroquinolone resistance among Egyptian *Salmonella*. El-Sherry [64] also observed complete resistance (100%) to amoxicillin and ampicillin, alongside high resistance to erythromycin (83.33%) and doxycycline (72.22%). Globally, resistance to a wide range of antimicrobials, including ampicillin, amoxicillin/clavulanic acid, ceftazidime, ceftriaxone, cefotaxime, imipenem, tetracycline, gentamicin, amikacin, kanamycin, streptomycin, nalidixic acid, ciprofloxacin, erythromycin, chloramphenicol, colistin, and trimethoprim/sulfamethoxazole, has been reported among *Salmonella* serovars [70].

The widespread resistance observed in this study can be explained by several factors identified through the questionnaire survey with farmers. Antimicrobials from multiple drug classes were frequently used, often repeatedly within the same rearing cycle, and included both veterinary and human pharmaceutical preparations. Drug administration was usually carried out by untrained personnel, with little awareness of legislation governing veterinary drug use and poor compliance with withdrawal periods. Interviews with farm owners and veterinarians further revealed that florfenicol, flumequine, danofloxacin, colistin, fosfomycin, thiamphenicol, and apramycin are commonly used for treating *Salmonella* infections, while tylosin and thiamphenicol are routinely applied as part of the reception protocol for one-day-old chicks. These findings highlight the misuse of veterinary drugs by unqualified individuals in Egypt’s poultry industry, contributing to the development of antimicrobial resistance and the presence of drug residues on farms. Accordingly, the present study provides important baseline data on antimicrobial resistance in Egyptian broiler farms.

Although colistin, fosfomycin, and apramycin are either banned or not officially registered for poultry use in Egypt, several reports indicate that these agents continue to be used in the field. This is likely due to weak enforcement of antimicrobial stewardship regulations and limited farmer awareness. Similar findings were reported by ElSayed et al. [71], who highlighted the ongoing use of last-resort antimicrobials in animal farms, and by Elmonir et al. [72], who detected colistin-resistant strains in Egyptian poultry. Pharmacokinetic and field observations have also shown that apramycin and fosfomycin are occasionally used off-label in broilers for enteric infections [72–74]. Our results therefore likely reflect true field practices rather than data bias, emphasizing the urgent need for stricter regulation, improved surveillance, and farmer education regarding antibiotic use in poultry production.

Despite the widespread resistance, moderate resistance levels were observed for amikacin (37.9%), suggesting that this antibiotic may still retain some therapeutic value. More importantly, carbapenems (meropenem and imipenem), considered last-resort treatments for MDR *Salmonella* infections, remained largely effective against the isolates tested. High susceptibility was observed to meropenem (96.6%) and, to a lesser extent, to imipenem (65.5%). However, the detection of intermediate resistance to imipenem is concerning and underscores the need for careful restriction of carbapenem use in animal health to prevent the emergence of carbapenem-resistant strains, alongside continuous monitoring of their efficacy.

Multidrug resistance (MDR) remains a growing global health concern. In the present study, *Salmonella* isolates showed a high prevalence of MDR, with multiple antibiotic resistance (MAR) indices ranging from 0.6 to 0.9. Importantly, all values exceeded the critical threshold of 0.2, confirming exposure to environments with intensive antimicrobial use, such as poultry farms. Seven isolates were classified as MDR (resistant to three or more antibiotic categories), while 22 isolates exhibited resistance to more than nine of the eleven antibiotic categories tested and were therefore classified as extensively drug-resistant (XDR). These findings strongly support the role of poultry production systems as reservoirs for MDR and XDR *Salmonella.*

Particularly concerning was the detection of newly reported serovars with extraordinarily high resistance levels. Isolates S7 and S8 (*S*. Colorado) and S15c (*S*. Jerusalem) displayed MAR indices of 0.8–0.9 and were categorized as XDR, resisting 17–20 of the 22 antimicrobials tested. The emergence of these serovars in Egypt is especially alarming, as they are rarely encountered locally, and little is known about their characteristics or epidemiological behavior. Their presence in the food supply chain therefore represents a major public health threat, particularly for vulnerable populations such as children, the elderly, and immunocompromised individuals. If disseminated through contaminated food or water, these isolates could cause limited treatment options, increased healthcare costs, and higher mortality. These findings underscore the outbreaks with urgent need for antimicrobial stewardship, enhanced surveillance programs, and strict infection control measures to prevent the spread of such highly resistant and unfamiliar pathogens.

 In the present study, statistical analysis revealed no significant association between *Salmonella* serotype and antimicrobial resistance category (MDR or XDR) (χ² = 6.61, df = 7, *p* = 0.471). This indicates that the distribution of resistance phenotypes among the investigated isolates was not serotype-dependent, suggesting that the dissemination of MDR and XDR traits is likely driven by horizontal gene transfer via plasmids, integrons, or transposons rather than clonal expansion within specific serovars [75]. Although certain serotypes, such as *S*. Kentucky and *S*. Derby, exhibited higher frequencies of XDR phenotypes, these differences were not statistically significant. Similar patterns have been documented in poultry production systems, where diverse *Salmonella* serotypes frequently harbor identical resistance determinants, reflecting the critical role of mobile genetic elements in disseminating antimicrobial resistance across lineages [76]. The predominance of XDR phenotypes across multiple serotypes observed in this study is consistent with global trends, where resistance determinants such as *bla*_CTX−M_, *bla*_TEM_, and *mcr* genes are widely disseminated irrespective of serotype [77]. These findings underscore the urgent need for integrated surveillance strategies in poultry production that combine phenotypic and genomic approaches to monitor the spread of resistance determinants and inform targeted interventions.

Broth microdilution (BMD) is recognized as the gold standard for determining the minimum inhibitory concentration (MIC) of colistin, since disk diffusion methods are unreliable due to the poor diffusion of colistin in agar [78]. In this study, colistin MICs were determined using the BMD method following EUCAST guidelines. Based on EUCAST breakpoints (MIC ≤ 2 mg/L = susceptible; MIC >2 mg/L = resistant), 27.6% (8/29) of the isolates were resistant to colistin, while 72.4% (21/29) were classified as susceptible.

Given the critical role of colistin as a last-resort therapeutic option, the detection of resistance in more than one-quarter of isolates is alarming. The unregulated and widespread use of colistin in livestock production, particularly as a growth promoter, likely contributes to this emerging resistance. These findings underscore the urgent need for strict regulation of colistin use in animal farming to preserve its efficacy in human medicine and to prevent further dissemination of colistin-resistant *Salmonella* along the food chain In this study, the distribution of β-lactam resistance genes was examined in 29 *Salmonella* isolates of poultry origin. The most prevalent gene was *bla*_TEM_ (82.8%), followed by *bla*_OXA−10_ (27.6%), *bla*_SHV_ (24.1%), and *bla*_CTX−M_ (24.1%). Less frequently detected genes included *bla*_CMY−2_ (10.3%) and *bla*_OXA−2_ (3.4%), while *bla*_VEB−1_ was not detected. Importantly, none of the isolates harbored carbapenemase genes, including *bla*_GES_, *bla*_OXA−48_, *bla*_IMP_, *bla*_VIM_, *bla*_KPC_, or *bla*_NDM−1._

To the best of our knowledge, this is the first report from Egypt documenting the presence of *bla*_OXA−10_ and *bla*_OXA−2_ in poultry-derived *Salmonella*. This novel finding expands current knowledge on OXA-type β-lactamase distribution in animal reservoirs and raises global public health concerns due to the potential for horizontal transfer of these resistance determinants to human pathogens via the food chain. Previous studies from Egypt and the Middle East have not reported these genes in poultry-associated *Salmonella*, highlighting the uniqueness of our results and underscoring the urgent need for continued molecular surveillance in the poultry sector.

Our results are generally consistent with earlier Egyptian reports, although variations in gene frequencies were observed. For instance, Abdel-Maksoud et al. [79] reported *bla*_TEM−1_ in 57.6% and *bla*_SHV−1_ in 6.8% of poultry isolates, with no detection of OXA-type genes. The prevalence of *bla*_TEM_ is lower than that reported in the present study, suggesting a possible increase in its dissemination over the past decade. Similarly, Adel et al. [80] detected *bla*_TEM−1_ in 79.4%, *bla*_CTX−M_ in 32.4%, *bla*_SHV−12_ in 14.7%, and *bla*_CMY−2_ in 26.5% of isolates, showing a higher frequency of *bla*_CMY−2_ than in our findings, potentially due to differences in poultry practices or antimicrobial usage policies. Kamel et al. [81] reported *bla*_TEM_ in 100% and *bla*_SHV_ in 20% of isolates, but no *bla*_CTX−M_, highlighting the dominant role of *bla*_TEM_ while suggesting that *bla*_CTX−M_ carriage may depend on local antimicrobial pressure or plasmid dissemination.

Notably, carbapenemase-encoding genes were absent in our isolates, likely reflecting the limited veterinary use of carbapenems in the study region and the resulting low selection pressure. Nevertheless, other Egyptian studies have reported their emergence: Abdel-Kader et al. [82] identified *bla*_KPC_ in *Salmonella* from chicken giblets, while another study detected *bla*_NDM_ in poultry-derived isolates [83], suggesting that poultry may act as a potential reservoir for the horizontal transfer of carbapenem resistance genes Taken together, these findings highlight the dynamic distribution of β-lactamase genes in poultry-associated *Salmonella* in Egypt. The emergence of OXA-type genes and the potential for horizontal transfer of resistance determinants underscore the need for broader molecular investigations, including whole-genome sequencing, to characterize their genetic contexts and assess their clinical and epidemiological relevance. Furthermore, although carbapenemase genes were not detected in this study, their sporadic occurrence in other Egyptian reports points to an emerging risk. Continuous surveillance using advanced molecular approaches is therefore recommended to enable early detection of these resistance determinants and to prevent their establishment in poultry production systems.

The mobilized colistin resistance (*mcr*) genes are plasmid-mediated determinants that confer resistance to colistin. These genes are frequently located on plasmids that also harbor ESBL genes, leading to co-resistance to both colistin and β-lactam antibiotics. Such co-localization poses a major public health threat, as it facilitates rapid horizontal gene transfer among Enterobacteriaceae and complicates therapeutic options for multidrug-resistant infections [14].

In the present study, genotypic analysis revealed the presence of the *mcr*−1 gene in 10.3% (3/29) of the isolates, while other variants (*mcr*−2, *mcr*−3, *mcr*−4, *mcr*−5, *mcr*−7.1, and *mcr*−9) were not detected. Previous reports from Egypt documented variable prevalence of *mcr*−1 among different Enterobacteriaceae species: Sadek et al. [84] detected *mcr*−1 in *E. coli* from retail chicken meat, while Elmonir et al. [72] described co-occurrence of colistin and carbapenem resistance in *Klebsiella pneumoniae* from poultry and humans. Similarly, Salem et al. [85] reported *mcr*−1 in 18.2% of *Pseudomonas aeruginosa* isolates from broiler chicks and dead in-shell chicks. These studies highlight the zoonotic potential of *mcr*-positive bacteria and their risk of transmission through the food chain. 

Globally, dissemination of *mcr* genes has been documented in poultry-associated *Salmonella*. For example, in Bangladesh, *mcr*−1 was detected in *Salmonella* from chickens [86]; in Korea, the *mcr*−9 gene was identified in *Salmonella enterica* from retail chicken meat [87]; and in China, *mcr*−1-positive *Salmonella* isolates were recovered from food samples [88].

Taken together, our findings confirm the emergence of plasmid-mediated colistin resistance in poultry-associated *Salmonella* in Egypt. This emphasizes the urgent need for enhanced surveillance and molecular characterization of resistance determinants, particularly in food-producing animals, to inform effective mitigation strategies and safeguard public health.

Integrons are genetic elements that play a critical role in the acquisition and dissemination of antimicrobial resistance genes, including β-lactamases and *mcr* genes, and they stimulate their transcription and expression. The capture and spread of antibiotic resistance determinants by integrons contribute to the rapid evolution of multidrug resistance (MDR) among Gram-negative bacteria [89].

In the present study, the class 1 integron gene (*intI*1) was detected in 96.6% of the *Salmonella* isolates, representing a notably high prevalence compared to previous reports in Egypt. Earlier studies documented variable prevalence rates of *intI*1 in poultry isolates, ranging from 39.1% [90] to 95% [91], with intermediate frequencies reported by Abdel-Maksoud et al. [79] (82%), Ahmed et al. [92] (80%), Elkenany et al. [93] (76.9%), and El-Sendiony et al. [45] (50%). Collectively, these results suggest a potential upward trend in integron dissemination among poultry-associated *Salmonella*, contributing to the persistence and spread of MDR strains.

In this study, only one isolate was found to lack the class 1 integron gene (*intI*1) and simultaneously tested negative for all screened antimicrobial resistance genes. This observation underscores the critical role of integrons in the acquisition and dissemination of resistance determinants among *Salmonella*. Interestingly, despite the absence of detectable resistance genes, this isolate exhibited phenotypic resistance to multiple antibiotics. Such resistance is likely mediated by alternative mechanisms or undetected genes not included in our PCR panel. Possible explanations include chromosomal mutations, efflux pump overexpression, or structural modifications in drug target sites. This discrepancy underscores the complexity of antimicrobial resistance and the necessity of broader genomic investigations to fully elucidate underlying mechanisms.

In this study, no significant associations were observed between *Salmonella* serotypes and the distribution of major antimicrobial resistance genes. Although some genes (*mcr*−1, *bla*_CMY−2_, and *bla*_OXA−2_) were detected in specific serotypes, the small number of positive isolates may have limited the statistical power to detect meaningful associations. The widespread presence of *bla*_TEM_ and *intI*1 across multiple serotypes suggests that horizontal gene transfer mediated by plasmids or integrons, rather than serotype-specific clonal expansion, is the predominant mechanism driving the dissemination of resistance genes.

The correlation analysis demonstrated clear genetic linkages between ESBL determinants and colistin resistance genes. Most concerning were the strong correlations between *bla*_CTX−M_, *mcr*−1, and *bla*_CMY−2_, as their co-localization on conjugative plasmids or transposons could enable simultaneous selection under antimicrobial pressure. In poultry systems where β-lactams and colistin are widely used or persist as residues, such linkages may accelerate the dissemination and stabilization of multidrug resistance. The predominance of *mcr*−1 as the key colistin resistance gene, together with its strong association with ESBLs, poses serious therapeutic challenges. Since colistin is considered a last-resort treatment for carbapenem and cephalosporin resistant Enterobacteriaceae, its genetic linkage with ESBL determinants indicates that colistin resistance can be co-selected even in the absence of direct exposure. This amplifies the risk of untreatable *Salmonella* infections and represents a critical barrier to effective antimicrobial stewardship.

Additionally, a moderate association between *bla*_TEM_ and *intI*1 indicates that integron-mediated transfer plays a central role in their persistence and spread across different *Salmonella* serotypes. Similarly, the association of *bla*_OXA−2_ with *bla*_CTX−M_ supports the hypothesis of shared mobilization pathways among β-lactamase genes. In contrast, carbapenemase genes showed no correlations with other resistance determinants, likely reflecting the limited or absent use of carbapenems in Egyptian poultry production and, consequently, minimal selection pressure.

Despite the valuable insights provided by this study, several limitations should be acknowledged. First, discrepancies were noted between phenotypic resistance and the genotypic detection of resistance genes, which may be attributed to untested resistance mechanisms, variability in gene expression, or limitations in methodological sensitivity. Second, although efforts were made to obtain a representative sample, the inclusion of mixed sample types (e.g., cloacal swabs, organs, and dead birds) without stratified analysis may have introduced sampling bias, particularly with respect to post-mortem bacterial overgrowth. Finally, the identification of rare serovars such as *S*. Jerusalem and *S*. Colorado relied solely on serotyping without confirmation through whole-genome sequencing (WGS) or core-genome multilocus sequence typing (cgMLST). This limits the ability to verify their novelty and assess clonality. Future research should incorporate these genomic approaches, and additional molecular studies are warranted to validate and expand upon the current findings.

## Conclusion

This study reveals a concerning diversity of *Salmonella enterica* serovars circulating in Egyptian poultry farms, including the detection of rare and highly drug-resistant serovars such as *S*. Jerusalem and *S*. Colorado. Notably, the *S*. Jerusalem isolate carried a cluster of critical resistance genes, including *bla*_TEM_, *bla*_SHV_, *bla*_CTX−M_, *mcr*−1, and *intI*1. The emergence of such atypical and potentially zoonotic strains poses a serious public health threat. Of particular concern is the co-occurrence of colistin resistance determinants with β-lactamase-encoding genes, especially *bla*_OXA−10_ and *bla*_OXA−2_, which are reported here for the first time, as both antibiotic classes, especially colistin, a last-resort treatment, are essential for managing life-threatening infections. The additional presence of class 1 integrons further exacerbates this risk by facilitating horizontal gene transfer, thereby accelerating the dissemination of multidrug resistance. Collectively, these findings highlight a dangerous convergence of resistance determinants that jeopardizes therapeutic options and increases the risk of untreatable infections in both veterinary and human health contexts. Urgent action is required through continuous surveillance, advanced molecular characterization, and the enforcement of stricter biosecurity and antimicrobial stewardship policies. Such measures are critical to contain the spread of both common and emerging *Salmonella* serovars of public health importance and to safeguard the efficacy of critically important antimicrobials.

## Supplementary Information

Supplementary Material 1

Supplementary Material 2

Supplementary Material 3

## Data Availability

The current study includes the entire information that was gathered throughout this manuscript. The data supporting the study conclusions are accessible from the authors upon request. Sequence data that support the findings of this study have been deposited in gene bank with the primary accession codes: PQ720689, PQ720690, PQ720691, PQ678532, PQ678533, PQ720692, PQ720693, PQ720694, PQ659181, PQ659182, PQ659183, and PQ659184.1.
