# Supplementary material for: Isolation and characterization of Salmonella enterica serovars from poultry in Egypt: a comprehensive genetic analysis of ESBLs, MCR, integron and other resistance genes
Source: BMC Vet Res. 2025 Nov 21;21:700. doi: 10.1186/s12917-025-05121-z (PMC12702162; doi:10.1186/s12917-025-05121-z)
Supplement: Supplementary file 1 — Supplementary Material 1 [file 12917_2025_5121_MOESM1_ESM.pdf]

Questionnaire

Section A: Farm Identification

1. Farm ID: Farm 1

2. Loca on (Governorate/District): Al Mansoura, Dakahlia

Section B: Demographic Information

1. What is your age?

☐ Under 30 ☑ 30–50 ☐ Over 50

2. What is your level of educa on?

☐ Illiterate ☐ Primary ☑ Secondary ☐ University

3. What is your role on the farm?

☐ Owner ☑ Worker

4. How many years have you been working in poultry farming?

☐ Less than 5 years ☑ 5–10 years ☐ More than 10 years

Section C: Farm and Flock Information

1. Type of poultry:

☑ Broiler ☐ Layer ☐ Breeder

2. Flock size: 300,000 birds

3. Age of birds at sampling: 21-25 days

4. Mortality observed in the past week: High mortality at 16 days → 230 birds
17 days → 222 birds
18 days → 430 birds
19 days → 615 birds

5. Clinical signs observed in the flock:

☑ Diarrhea

☑ Anorexia (loss of appetite)

☐ Ruffled feathers

☑ Respiratory signs (coughing/sneezing)

☐ unabsorbed yolk sac

☑ Depression or inactivity

☐ Neurological signs (tremors, twisting of neck)

☐ Other: ________

CS CamScanner

6. Known or suspected diseases on the farm (past or current):

☐ Newcastle disease (ND)

☑ Infectious Bursal Disease (IBD / Gumboro)

☑ Infectious Bronchitis (IB)

☐ Avian Influenza (AI)

☐ Colibacillosis (E. coli)

☐ Salmonellosis

☐ Other: ________

Section D: Husbandry Practices

1. What is your source of drinking water for the flock?

☑ Tap water ☐ Well ☐ Surface water

2. Do you clean and disinfect the poultry house regularly?

☑ Yes ☐ No

3. Is rodent or wild bird access controlled on the farm?

☑ Yes ☐ No

Section E: Antibiotic Use

1. Have you used antibiotics in this flock cycle?

☑ Yes ☐ No

2. Use of antibiotics in the past 2 weeks?

☑ Yes ☐ No Florfenicol, fosfomycin, Apramycin and Colistin

3. If yes, who recommended the antibiotic?

☑ Veterinarian ☐ Feed supplier ☐ Self-decision

4. Do you follow the withdrawal period before slaughtering?

☑ Yes ☐ No ☐ Not sure

5. Do you use the same antibiotics in multiple flock cycles?

☑ Yes ☐ No

CS CamScanner

Section F: Knowledge about Salmonella spp.

1. Have you ever heard of Salmonella infection in poultry?

☑ Yes ☐ No

2. Do you know that Salmonella can be transmitted to humans through meat or eggs?

☑ Yes ☐ No

3. Have you ever had a confirmed case of Salmonella on your farm?

☑ Yes ☐ No ☐ Not sure

4. Do you take any specific measures to prevent Salmonella infection?

☑ Yes ☐ No

CamScanner

Questionnaire

Section A: Farm Identification

1. Farm ID: Farm 2
2. Loca on (Governorate/District): AL Mansoura, Dakahlia

Section B: Demographic Information

1. What is your age?

☐ Under 30 ☑ 30–50 ☐ Over 50

2. What is your level of educa on?

☐ Illiterate ☐ Primary ☑ Secondary ☐ University

3. What is your role on the farm?

☐ Owner ☑ Worker

4. How many years have you been working in poultry farming?

☐ Less than 5 years ☑ 5–10 years ☐ More than 10 years

Section C: Farm and Flock Information

1. Type of poultry:

☑ Broiler ☐ Layer ☐ Breeder

2. Flock size: 30,000 birds
3. Age of birds at sampling: 22 days
4. Mortality observed in the past week: At 19 days → 29 birds
21 days → 30 birds
22 days → 50 birds
5. Clinical signs observed in the flock:

☑ Diarrhea

☑ Anorexia (loss of appetite)

☐ Ruffled feathers

☐ Respiratory signs (coughing/sneezing)

☐ unabsorbed yolk sac

☑ Depression or inactivity

☐ Neurological signs (tremors, twisting of neck)

☐ Other: _________

CS CamScanner

6. Known or suspected diseases on the farm (past or current):

☐ Newcastle disease (ND)

☑ Infectious Bursal Disease (IBD / Gumboro)

☑ Infectious Bronchitis (IB)

☐ Avian Influenza (AI)

☐ Colibacillosis (E. coli)

☐ Salmonellosis

☐ Other: ________

Section D: Husbandry Practices

1. What is your source of drinking water for the flock?

☑ Tap water ☐ Well ☐ Surface water

2. Do you clean and disinfect the poultry house regularly?

☑ Yes ☐ No

3. Is rodent or wild bird access controlled on the farm?

☑ Yes ☐ No

Section E: Antibiotic Use

1. Have you used antibiotics in this flock cycle?

☑ Yes ☐ No

2. Use of antibiotics in the past 2 weeks?

☑ Yes ☐ No Florfenicol, Fosfomycin, APramycin and colistin

3. If yes, who recommended the antibiotic?

☑ Veterinarian ☐ Feed supplier ☐ Self-decision

4. Do you follow the withdrawal period before slaughtering?

☑ Yes ☐ No ☐ Not sure

5. Do you use the same antibiotics in multiple flock cycles?

☑ Yes ☐ No

CS CamScanner

Section F: Knowledge about Salmonella spp.

1. Have you ever heard of Salmonella infection in poultry?

☑ Yes ☐ No

2. Do you know that Salmonella can be transmitted to humans through meat or eggs?

☑ Yes ☐ No

3. Have you ever had a confirmed case of Salmonella on your farm?

☑ Yes ☐ No ☐ Not sure

4. Do you take any specific measures to prevent Salmonella infection?

☑ Yes ☐ No

CS CamScanner

Questionnaire

Section A: Farm Identification

1. Farm ID: Farm 3

2. Loca on (Governorate/District): AL Mansoura, Dakahlia

Section B: Demographic Information

1. What is your age?

☑ Under 30 ☐ 30–50 ☐ Over 50

2. What is your level of educa on?

☐ Illiterate ☑ Primary ☐ Secondary ☐ University

3. What is your role on the farm?

☑ Owner ☐ Worker

4. How many years have you been working in poultry farming?

☐ Less than 5 years ☑ 5–10 years ☐ More than 10 years

Section C: Farm and Flock Information

1. Type of poultry:

☑ Broiler ☐ Layer ☐ Breeder

2. Flock size: 2,600 birds

3. Age of birds at sampling: 35 days

4. Mortality observed in the past week: High mortality rate

5. Clinical signs observed in the flock:

☑ Diarrhea

☑ Anorexia (loss of appetite)

☐ Ruffled feathers

☑ Respiratory signs (coughing/sneezing)

☐ unabsorbed yolk sac

☑ Depression or inactivity

☐ Neurological signs (tremors, twisting of neck)

☐ Other: __________

CS CamScanner

6. Known or suspected diseases on the farm (past or current):

☑ Newcastle disease (ND)

☑ Infectious Bursal Disease (IBD / Gumboro)

☑ Infectious Bronchitis (IB)

☐ Avian Influenza (AI)

☑ Colibacillosis (E. coli)

☐ Salmonellosis

☐ Other: CRD

Section D: Husbandry Practices

1. What is your source of drinking water for the flock?

☑ Tap water ☐ Well ☐ Surface water

2. Do you clean and disinfect the poultry house regularly?

☑ Yes ☐ No

3. Is rodent or wild bird access controlled on the farm?

☐ Yes ☑ No

Section E: Antibiotic Use

1. Have you used antibiotics in this flock cycle?

☑ Yes ☐ No

2. Use of antibiotics in the past 2 weeks?

☑ Yes ☐ No Apramycin and colistin

3. If yes, who recommended the antibiotic?

☐ Veterinarian ☐ Feed supplier ☑ Self-decision

4. Do you follow the withdrawal period before slaughtering?

☐ Yes ☐ No ☑ Not sure

5. Do you use the same antibiotics in multiple flock cycles?

☑ Yes ☐ No

CamScanner

Section F: Knowledge about Salmonella spp.

1. Have you ever heard of Salmonella infection in poultry?

☑ Yes ☐ No

2. Do you know that Salmonella can be transmitted to humans through meat or eggs?

☑ Yes ☐ No

3. Have you ever had a confirmed case of Salmonella on your farm?

☑ Yes ☐ No ☐ Not sure

4. Do you take any specific measures to prevent Salmonella infection?

☑ Yes ☐ No

CamScanner

Questionnaire

Section A: Farm Identification

1. Farm ID: Farm 4
2. Loca on (Governorate/District): AL Mansoura, Dakahlia

Section B: Demographic Information

1. What is your age?

☑ Under 30 ☐ 30–50 ☐ Over 50

2. What is your level of educa on?

☐ Illiterate ☑ Primary ☐ Secondary ☐ University

3. What is your role on the farm?

☑ Owner ☐ Worker

4. How many years have you been working in poultry farming?

☐ Less than 5 years ☑ 5–10 years ☐ More than 10 years

Section C: Farm and Flock Information

1. Type of poultry:

☑ Broiler ☐ Layer ☐ Breeder

2. Flock size: 16,000 birds
3. Age of birds at sampling: 27 days
4. Mortality observed in the past week: 20 birds
5. Clinical signs observed in the flock:

☑ Diarrhea

☑ Anorexia (loss of appetite)

☐ Ruffled feathers

☑ Respiratory signs (coughing/sneezing)

☐ unabsorbed yolk sac

☑ Depression or inactivity

☐ Neurological signs (tremors, twisting of neck)

☐ Other: ________

CamScanner

6. Known or suspected diseases on the farm (past or current):

☑ Newcastle disease (ND)

☑ Infectious Bursal Disease (IBD / Gumboro)

☑ Infectious Bronchitis (IB)

☐ Avian Influenza (AI)

☐ Colibacillosis (E. coli)

☐ Salmonellosis

☐ Other: CRD

Section D: Husbandry Practices

1. What is your source of drinking water for the flock?

☑ Tap water ☐ Well ☐ Surface water

2. Do you clean and disinfect the poultry house regularly?

☑ Yes ☐ No

3. Is rodent or wild bird access controlled on the farm?

☐ Yes ☑ No

Section E: Antibiotic Use

1. Have you used antibiotics in this flock cycle?

☑ Yes ☐ No

2. Use of antibiotics in the past 2 weeks?

☑ Yes ☐ No Florfenicol, Apramycin and Colistin

3. If yes, who recommended the antibiotic?

☐ Veterinarian ☐ Feed supplier ☑ Self-decision

4. Do you follow the withdrawal period before slaughtering?

☐ Yes ☐ No ☑ Not sure

5. Do you use the same antibiotics in multiple flock cycles?

☑ Yes ☐ No

CS CamScanner

Section F: Knowledge about Salmonella spp.

1. Have you ever heard of Salmonella infection in poultry?

☑ Yes ☐ No

2. Do you know that Salmonella can be transmitted to humans through meat or eggs?

☑ Yes ☐ No

3. Have you ever had a confirmed case of Salmonella on your farm?

☑ Yes ☐ No ☐ Not sure

4. Do you take any specific measures to prevent Salmonella infection?

☑ Yes ☐ No

CS CamScanner

Questionnaire

Section A: Farm Identification

1. Farm ID: Farm 5
2. Loca on (Governorate/District): AL Mansoura, Dakahlia

Section B: Demographic Information

1. What is your age?

☐ Under 30 ☑ 30–50 ☐ Over 50

2. What is your level of educa on?

☐ Illiterate ☑ Primary ☐ Secondary ☐ University

3. What is your role on the farm?

☑ Owner ☐ Worker

4. How many years have you been working in poultry farming?

☐ Less than 5 years ☑ 5–10 years ☐ More than 10 years

Section C: Farm and Flock Information

1. Type of poultry:

☑ Broiler ☐ Layer ☐ Breeder

2. Flock size: 6,000 birds
3. Age of birds at sampling: 31 days
4. Mortality observed in the past week: 40 birds
5. Clinical signs observed in the flock:

☑ Diarrhea

☑ Anorexia (loss of appetite)

☑ Ruffled feathers

☑ Respiratory signs (coughing/sneezing)

☐ unabsorbed yolk sac

☑ Depression or inactivity

☐ Neurological signs (tremors, twisting of neck)

☐ Other: Small body sizes

CS CamScanner

6. Known or suspected diseases on the farm (past or current):

☑ Newcastle disease (ND)

☑ Infectious Bursal Disease (IBD / Gumboro)

☑ Infectious Bronchitis (IB)

☐ Avian Influenza (AI)

☐ Colibacillosis (E. coli)

☐ Salmonellosis

☐ Other: CRD

Section D: Husbandry Practices

1. What is your source of drinking water for the flock?

☑ Tap water ☐ Well ☐ Surface water

2. Do you clean and disinfect the poultry house regularly?

☐ Yes ☑ No

3. Is rodent or wild bird access controlled on the farm?

☐ Yes ☑ No

Section E: Antibiotic Use

1. Have you used antibiotics in this flock cycle?

☑ Yes ☐ No

2. Use of antibiotics in the past 2 weeks?

☑ Yes ☐ No fosfomycin, lincomycin, streptomycin and tylvalosin

3. If yes, who recommended the antibiotic?

☐ Veterinarian ☐ Feed supplier ☑ Self-decision

4. Do you follow the withdrawal period before slaughtering?

☐ Yes ☐ No ☑ Not sure

5. Do you use the same antibiotics in multiple flock cycles?

☑ Yes ☐ No

CS CamScanner

Section F: Knowledge about Salmonella spp.

1. Have you ever heard of Salmonella infection in poultry?

☑ Yes ☐ No

2. Do you know that Salmonella can be transmitted to humans through meat or eggs?

☑ Yes ☐ No

3. Have you ever had a confirmed case of Salmonella on your farm?

☑ Yes ☐ No ☐ Not sure

4. Do you take any specific measures to prevent Salmonella infection?

☑ Yes ☐ No

CS CamScanner

Questionnaire

Section A: Farm Identification

1. Farm ID: Farm6

2. Loca on (Governorate/District): Meniet EL-Nasr, Dakahlia

Section B: Demographic Information

1. What is your age?

☐ Under 30 ☑ 30–50 ☐ Over 50

2. What is your level of educa on?

☐ Illiterate ☐ Primary ☑ Secondary ☐ University

3. What is your role on the farm?

☑ Owner ☐ Worker

4. How many years have you been working in poultry farming?

☐ Less than 5 years ☐ 5–10 years ☑ More than 10 years

Section C: Farm and Flock Information

1. Type of poultry:

☑ Broiler ☐ Layer ☐ Breeder

2. Flock size: unknown birds

3. Age of birds at sampling: 6 days

4. Mortality observed in the past week: 6 birds

5. Clinical signs observed in the flock:

☑ Diarrhea

☑ Anorexia (loss of appetite)

☑ Ruffled feathers

☑ Respiratory signs (coughing/sneezing)

☐ unabsorbed yolk sac

☑ Depression or inactivity

☐ Neurological signs (tremors, twisting of neck)

☐ Other: ________

CS CamScanner

6. Known or suspected diseases on the farm (past or current):

☐ Newcastle disease (ND)

☐ Infectious Bursal Disease (IBD / Gumboro)

☐ Infectious Bronchitis (IB)

☐ Avian Influenza (AI)

☐ Colibacillosis (E. coli)

☑ Salmonellosis

☐ Other: ________

Section D: Husbandry Practices

1. What is your source of drinking water for the flock?

☑ Tap water ☐ Well ☐ Surface water

2. Do you clean and disinfect the poultry house regularly?

☑ Yes ☐ No

3. Is rodent or wild bird access controlled on the farm?

☐ Yes ☑ No

Section E: Antibiotic Use

1. Have you used antibiotics in this flock cycle?

☑ Yes ☐ No

2. Use of antibiotics in the past 2 weeks?

☑ Yes ☐ No Colistin

3. If yes, who recommended the antibiotic?

☐ Veterinarian ☐ Feed supplier ☑ Self-decision

4. Do you follow the withdrawal period before slaughtering?

☐ Yes ☐ No ☑ Not sure

5. Do you use the same antibiotics in multiple flock cycles?

☑ Yes ☐ No

CamScanner

Section F: Knowledge about Salmonella spp.

1. Have you ever heard of Salmonella infection in poultry?

☑ Yes ☐ No

2. Do you know that Salmonella can be transmitted to humans through meat or eggs?

☑ Yes ☐ No

3. Have you ever had a confirmed case of Salmonella on your farm?

☑ Yes ☐ No ☐ Not sure

4. Do you take any specific measures to prevent Salmonella infection?

☑ Yes ☐ No

CS CamScanner

Questionnaire

Section A: Farm Identification

1. Farm ID: Farm 7

2. Loca on (Governorate/District): Meniet El-Nasr, Dakahlia

Section B: Demographic Information

1. What is your age?

☐ Under 30 ☑ 30–50 ☐ Over 50

2. What is your level of educa on?

☐ Illiterate ☐ Primary ☐ Secondary ☑ University

3. What is your role on the farm?

☑ Owner ☐ Worker

4. How many years have you been working in poultry farming?

☐ Less than 5 years ☐ 5–10 years ☑ More than 10 years

Section C: Farm and Flock Information

1. Type of poultry:

☑ Broiler ☐ Layer ☐ Breeder

2. Flock size: 24,000 birds

3. Age of birds at sampling: 1 days

4. Mortality observed in the past week: —

5. Clinical signs observed in the flock:

☐ Diarrhea

☐ Anorexia (loss of appetite)

☐ Ruffled feathers

☐ Respiratory signs (coughing/sneezing)

☐ unabsorbed yolk sac

☑ Depression or inactivity

☐ Neurological signs (tremors, twisting of neck)

☐ Other: huddling together

6. Known or suspected diseases on the farm (past or current):

☐ Newcastle disease (ND)

☐ Infectious Bursal Disease (IBD / Gumboro)

☐ Infectious Bronchitis (IB)

☐ Avian Influenza (AI)

☐ Colibacillosis (E. coli)

☐ Salmonellosis

☐ Other: ________

Section D: Husbandry Practices

1. What is your source of drinking water for the flock?

☑ Tap water ☐ Well ☐ Surface water

2. Do you clean and disinfect the poultry house regularly?

☑ Yes ☐ No

3. Is rodent or wild bird access controlled on the farm?

☑ Yes ☐ No

Section E: Antibiotic Use

1. Have you used antibiotics in this flock cycle?

☑ Yes ☐ No (Colistin) in the first day

2. Use of antibiotics in the past 2 weeks?

☐ Yes ☐ No

3. If yes, who recommended the antibiotic?

☐ Veterinarian ☐ Feed supplier ☑ Self-decision

4. Do you follow the withdrawal period before slaughtering?

☐ Yes ☐ No ☑ Not sure

5. Do you use the same antibiotics in multiple flock cycles?

☑ Yes ☐ No

CamScanner

Section F: Knowledge about Salmonella spp.

1. Have you ever heard of Salmonella infection in poultry?

☑ Yes ☐ No

2. Do you know that Salmonella can be transmitted to humans through meat or eggs?

☑ Yes ☐ No

3. Have you ever had a confirmed case of Salmonella on your farm?

☑ Yes ☐ No ☐ Not sure

4. Do you take any specific measures to prevent Salmonella infection?

☑ Yes ☐ No

CS CamScanner

Questionnaire

Section A: Farm Identification

1. Farm ID: Farm 8

2. Loca on (Governorate/District): AL Mansoura, Dakahlia

Section B: Demographic Information

1. What is your age?

☐ Under 30 ☑ 30–50 ☐ Over 50

2. What is your level of educa on?

☐ Illiterate ☐ Primary ☑ Secondary ☐ University

3. What is your role on the farm?

☑ Owner ☐ Worker

4. How many years have you been working in poultry farming?

☐ Less than 5 years ☐ 5–10 years ☑ More than 10 years

Section C: Farm and Flock Information

1. Type of poultry:

☑ Broiler ☐ Layer ☐ Breeder

2. Flock size: 5000 birds

3. Age of birds at sampling: 29 days

4. Mortality observed in the past week: ___

5. Clinical signs observed in the flock:

☑ Diarrhea

☑ Anorexia (loss of appetite)

☑ Ruffled feathers

☑ Respiratory signs (coughing/sneezing)

☐ unabsorbed yolk sac

☐ Depression or inactivity

☐ Neurological signs (tremors, twisting of neck)

☐ Other: ________

CamScanner

6. Known or suspected diseases on the farm (past or current):

☐ Newcastle disease (ND)

☐ Infectious Bursal Disease (IBD / Gumboro)

☐ Infectious Bronchitis (IB)

☐ Avian Influenza (AI)

☐ Colibacillosis (E. coli)

☐ Salmonellosis

☐ Other: __________

Section D: Husbandry Practices

1. What is your source of drinking water for the flock?

☑ Tap water ☐ Well ☐ Surface water

2. Do you clean and disinfect the poultry house regularly?

☑ Yes ☐ No

3. Is rodent or wild bird access controlled on the farm?

☐ Yes ☑ No

Section E: Antibiotic Use

1. Have you used antibiotics in this flock cycle?

☑ Yes ☐ No

2. Use of antibiotics in the past 2 weeks?

☑ Yes ☐ No Flumequine - Danofloxcin and Colistin

3. If yes, who recommended the antibiotic?

☑ Veterinarian ☐ Feed supplier ☐ Self-decision

4. Do you follow the withdrawal period before slaughtering?

☑ Yes ☐ No ☐ Not sure

5. Do you use the same antibiotics in multiple flock cycles?

☑ Yes ☐ No

CS CamScanner

Section F: Knowledge about Salmonella spp.

1. Have you ever heard of Salmonella infection in poultry?

☑ Yes ☐ No

2. Do you know that Salmonella can be transmitted to humans through meat or eggs?

☑ Yes ☐ No

3. Have you ever had a confirmed case of Salmonella on your farm?

☑ Yes ☐ No ☐ Not sure

4. Do you take any specific measures to prevent Salmonella infection?

☑ Yes ☐ No

CS CamScanner

Questionnaire

Section A: Farm Identification

1. Farm ID: Farm 9

2. Loca on (Governorate/District): AL Mansoura, Dakahlia

Section B: Demographic Information

1. What is your age?

☐ Under 30 ☑ 30–50 ☐ Over 50

2. What is your level of educa on?

☐ Illiterate ☐ Primary ☐ Secondary ☑ University

3. What is your role on the farm?

☑ Owner ☐ Worker

4. How many years have you been working in poultry farming?

☐ Less than 5 years ☐ 5–10 years ☑ More than 10 years

Section C: Farm and Flock Information

1. Type of poultry:

☑ Broiler ☐ Layer ☐ Breeder

2. Flock size: ________ birds

3. Age of birds at sampling: 12 days

4. Mortality observed in the past week: —

5. Clinical signs observed in the flock:

☑ Diarrhea

☐ Anorexia (loss of appetite)

☑ Ruffled feathers

☑ Respiratory signs (coughing/sneezing)

☐ unabsorbed yolk sac

☑ Depression or inactivity

☐ Neurological signs (tremors, twisting of neck)

☐ Other: __________

CamScanner

6. Known or suspected diseases on the farm (past or current):

☐ Newcastle disease (ND)

☐ Infectious Bursal Disease (IBD / Gumboro)

☐ Infectious Bronchitis (IB)

☐ Avian Influenza (AI)

☐ Colibacillosis (E. coli)

☒ Salmonellosis

☐ Other: __________

Section D: Husbandry Practices

1. What is your source of drinking water for the flock?

☒ Tap water ☐ Well ☐ Surface water

2. Do you clean and disinfect the poultry house regularly?

☐ Yes ☒ No

3. Is rodent or wild bird access controlled on the farm?

☐ Yes ☒ No

Section E: Antibiotic Use

1. Have you used antibiotics in this flock cycle?

☒ Yes ☐ No

2. Use of antibiotics in the past 2 weeks?

☒ Yes ☐ No Colistin, thiamPhenicol and Fosfomycin

3. If yes, who recommended the antibiotic?

☒ Veterinarian ☐ Feed supplier ☐ Self-decision

4. Do you follow the withdrawal period before slaughtering?

☐ Yes ☒ No ☐ Not sure

5. Do you use the same antibiotics in multiple flock cycles?

☒ Yes ☐ No

CamScanner

Section F: Knowledge about Salmonella spp.

1. Have you ever heard of Salmonella infection in poultry?

   ☑ Yes ☐ No

2. Do you know that Salmonella can be transmitted to humans through meat or eggs?

   ☑ Yes ☐ No

3. Have you ever had a confirmed case of Salmonella on your farm?

   ☑ Yes ☐ No ☐ Not sure

4. Do you take any specific measures to prevent Salmonella infection?

   ☑ Yes ☐ No

CS CamScanner

Questionnaire

Section A: Farm Identification

1. Farm ID: Farm 10

2. Loca on (Governorate/District): ALSharqia Governorate

Section B: Demographic Information

1. What is your age?

☐ Under 30 ☑ 30–50 ☐ Over 50

2. What is your level of educa on?

☐ Illiterate ☐ Primary ☑ Secondary ☐ University

3. What is your role on the farm?

☑ Owner ☐ Worker

4. How many years have you been working in poultry farming?

☐ Less than 5 years ☐ 5–10 years ☑ More than 10 years

Section C: Farm and Flock Information

1. Type of poultry:

☑ Broiler ☐ Layer ☐ Breeder (Sasso)

2. Flock size: 5000 birds

3. Age of birds at sampling: 50 days

4. Mortality observed in the past week: ——

5. Clinical signs observed in the flock:

☑ Diarrhea

☐ Anorexia (loss of appetite)

☐ Ruffled feathers

☑ Respiratory signs (coughing/sneezing)

☐ unabsorbed yolk sac

☑ Depression or inactivity

☐ Neurological signs (tremors, twisting of neck)

☐ Other: ________

CS CamScanner

6. Known or suspected diseases on the farm (past or current):

☐ Newcastle disease (ND)

☐ Infectious Bursal Disease (IBD / Gumboro)

☐ Infectious Bronchitis (IB)

☐ Avian Influenza (AI)

☐ Colibacillosis (E. coli)

☒ Salmonellosis

☐ Other: ________

Section D: Husbandry Practices

1. What is your source of drinking water for the flock?

☒ Tap water ☐ Well ☐ Surface water

2. Do you clean and disinfect the poultry house regularly?

☐ Yes ☒ No

3. Is rodent or wild bird access controlled on the farm?

☐ Yes ☒ No

Section E: Antibiotic Use

1. Have you used antibiotics in this flock cycle?

☒ Yes ☐ No

2. Use of antibiotics in the past 2 weeks? thiamphenicol, Apramycin, Colistin

☒ Yes ☐ No

3. If yes, who recommended the antibiotic?

☒ Veterinarian ☐ Feed supplier ☐ Self-decision

4. Do you follow the withdrawal period before slaughtering?

☐ Yes ☐ No ☒ Not sure

5. Do you use the same antibiotics in multiple flock cycles?

☒ Yes ☐ No

CamScanner

Section F: Knowledge about Salmonella spp.

1. Have you ever heard of Salmonella infection in poultry?

☒ Yes ☐ No

2. Do you know that Salmonella can be transmitted to humans through meat or eggs?

☐ Yes ☒ No

3. Have you ever had a confirmed case of Salmonella on your farm?

☒ Yes ☐ No ☐ Not sure

4. Do you take any specific measures to prevent Salmonella infection?

☒ Yes ☐ No

CamScanner

Questionnaire

Section A: Farm Identification

1. Farm ID: Farm 11

2. Loca on (Governorate/District): Kafr El-sheikh, Dakahlia

Section B: Demographic Information

1. What is your age?

☐ Under 30 ☐ 30–50 ☒ Over 50

2. What is your level of educa on?

☐ Illiterate ☐ Primary ☒ Secondary ☐ University

3. What is your role on the farm?

☒ Owner ☐ Worker

4. How many years have you been working in poultry farming?

☐ Less than 5 years ☐ 5–10 years ☒ More than 10 years

Section C: Farm and Flock Information

1. Type of poultry:

☒ Broiler ☐ Layer ☐ Breeder

2. Flock size: — birds

3. Age of birds at sampling: 29 days

4. Mortality observed in the past week: —

5. Clinical signs observed in the flock:

☒ Diarrhea

☐ Anorexia (loss of appetite)

☐ Ruffled feathers

☒ Respiratory signs (coughing/sneezing)

☐ unabsorbed yolk sac

☒ Depression or inactivity

☐ Neurological signs (tremors, twisting of neck)

☐ Other: ________

CamScanner

6. Known or suspected diseases on the farm (past or current):

☐ Newcastle disease (ND)

☐ Infectious Bursal Disease (IBD / Gumboro)

☐ Infectious Bronchitis (IB)

☐ Avian Influenza (AI)

☐ Colibacillosis (E. coli)

☒ Salmonellosis

☐ Other: CRD

Section D: Husbandry Practices

1. What is your source of drinking water for the flock?

☒ Tap water ☐ Well ☐ Surface water

2. Do you clean and disinfect the poultry house regularly?

☒ Yes ☐ No

3. Is rodent or wild bird access controlled on the farm?

☐ Yes ☒ No

Section E: Antibiotic Use

1. Have you used antibiotics in this flock cycle?

☒ Yes ☐ No

2. Use of antibiotics in the past 2 weeks?

☒ Yes ☐ No thiamphenicol

3. If yes, who recommended the antibiotic?

☐ Veterinarian ☐ Feed supplier ☒ Self-decision

4. Do you follow the withdrawal period before slaughtering?

☐ Yes ☐ No ☒ Not sure

5. Do you use the same antibiotics in multiple flock cycles?

☒ Yes ☐ No

CS CamScanner

Section F: Knowledge about Salmonella spp.

1. Have you ever heard of Salmonella infection in poultry?

☑ Yes ☐ No

2. Do you know that Salmonella can be transmitted to humans through meat or eggs?

☑ Yes ☐ No

3. Have you ever had a confirmed case of Salmonella on your farm?

☑ Yes ☐ No ☐ Not sure

4. Do you take any specific measures to prevent Salmonella infection?

☑ Yes ☐ No

CS CamScanner

Questionnaire

Section A: Farm Identification

1. Farm ID: Farm 12

2. Loca on (Governorate/District): Damietta City

Section B: Demographic Information

1. What is your age?

☐ Under 30 ☒ 30–50 ☐ Over 50

2. What is your level of education?

☐ Illiterate ☐ Primary ☒ Secondary ☐ University

3. What is your role on the farm?

☒ Owner ☐ Worker

4. How many years have you been working in poultry farming?

☐ Less than 5 years ☐ 5–10 years ☒ More than 10 years

Section C: Farm and Flock Information

1. Type of poultry:

☒ Broiler ☐ Layer ☐ Breeder

2. Flock size: — birds

3. Age of birds at sampling: — days

4. Mortality observed in the past week: —

5. Clinical signs observed in the flock:

☒ Diarrhea

☐ Anorexia (loss of appetite)

☒ Ruffled feathers

☒ Respiratory signs (coughing/sneezing)

☐ unabsorbed yolk sac

☒ Depression or inactivity

☐ Neurological signs (tremors, twisting of neck)

☐ Other: ________

CS CamScanner

6. Known or suspected diseases on the farm (past or current):

☐ Newcastle disease (ND)

☐ Infectious Bursal Disease (IBD / Gumboro)

☐ Infectious Bronchitis (IB)

☐ Avian Influenza (AI)

☐ Colibacillosis (E. coli)

☒ Salmonellosis

☐ Other: CRD

Section D: Husbandry Practices

1. What is your source of drinking water for the flock?

☒ Tap water ☐ Well ☐ Surface water

2. Do you clean and disinfect the poultry house regularly?

☐ Yes ☒ No

3. Is rodent or wild bird access controlled on the farm?

☐ Yes ☒ No

Section E: Antibiotic Use

1. Have you used antibiotics in this flock cycle?

☒ Yes ☐ No

2. Use of antibiotics in the past 2 weeks?

☒ Yes ☐ No Apramycin, thiamphenicol

3. If yes, who recommended the antibiotic?

☒ Veterinarian ☐ Feed supplier ☐ Self-decision

4. Do you follow the withdrawal period before slaughtering?

☐ Yes ☒ No ☐ Not sure

5. Do you use the same antibiotics in multiple flock cycles?

☒ Yes ☐ No

CS CamScanner

Section F: Knowledge about Salmonella spp.

1. Have you ever heard of Salmonella infection in poultry?

☒ Yes ☐ No

2. Do you know that Salmonella can be transmitted to humans through meat or eggs?

☒ Yes ☐ No

3. Have you ever had a confirmed case of Salmonella on your farm?

☒ Yes ☐ No ☐ Not sure

4. Do you take any specific measures to prevent Salmonella infection?

☒ Yes ☐ No

CS CamScanner

Questionnaire

Section A: Farm Identification

1. Farm ID: Farm 13

2. Loca on (Governorate/District): AL Mansoura, Dakahlia

Section B: Demographic Information

1. What is your age?

   ☐ Under 30 ☒ 30–50 ☐ Over 50

2. What is your level of educa ion?

   ☐ Illiterate ☐ Primary ☒ Secondary ☐ University

3. What is your role on the farm?

   ☒ Owner ☐ Worker

4. How many years have you been working in poultry farming?

   ☐ Less than 5 years ☐ 5–10 years ☒ More than 10 years

Section C: Farm and Flock Information

1. Type of poultry:

   ☒ Broiler ☐ Layer ☐ Breeder

2. Flock size: [redacted] birds

3. Age of birds at sampling: 13 days

4. Mortality observed in the past week: [redacted]

5. Clinical signs observed in the flock:

   ☐ Diarrhea

   ☒ Anorexia (loss of appetite)

   ☐ Ruffled feathers

   ☒ Respiratory signs (coughing/sneezing)

   ☒ unabsorbed yolk sac

   ☒ Depression or inactivity

   ☐ Neurological signs (tremors, twisting of neck)

   ☐ Other: Poor growth

CS CamScanner

6. Known or suspected diseases on the farm (past or current):

☐ Newcastle disease (ND)

☐ Infectious Bursal Disease (IBD / Gumboro)

☐ Infectious Bronchitis (IB)

☐ Avian Influenza (AI)

☐ Colibacillosis (E. coli)

☒ Salmonellosis

☐ Other: CRD

Section D: Husbandry Practices

1. What is your source of drinking water for the flock?

☒ Tap water ☐ Well ☐ Surface water

2. Do you clean and disinfect the poultry house regularly?

☒ Yes ☐ No

3. Is rodent or wild bird access controlled on the farm?

☐ Yes ☒ No

Section E: Antibiotic Use

1. Have you used antibiotics in this flock cycle?

☒ Yes ☐ No

2. Use of antibiotics in the past 2 weeks?

☒ Yes ☐ No thiamPhenicol, APramycin

3. If yes, who recommended the antibiotic?

☐ Veterinarian ☐ Feed supplier ☒ Self-decision

4. Do you follow the withdrawal period before slaughtering?

☒ Yes ☐ No ☐ Not sure

5. Do you use the same antibiotics in multiple flock cycles?

☒ Yes ☐ No

CS CamScanner

Section F: Knowledge about Salmonella spp.

1. Have you ever heard of Salmonella infection in poultry?

☒ Yes ☐ No

2. Do you know that Salmonella can be transmitted to humans through meat or eggs?

☒ Yes ☐ No

3. Have you ever had a confirmed case of Salmonella on your farm?

☒ Yes ☐ No ☐ Not sure

4. Do you take any specific measures to prevent Salmonella infection?

☒ Yes ☐ No

CS CamScanner

—

Questionnaire

Section A: Farm Identification

1. Farm ID: Farm 14

2. Loca on (Governorate/District): AL Mansoura, Dakahlia

Section B: Demographic Information

1. What is your age?

☐ Under 30 ☒ 30–50 ☐ Over 50

2. What is your level of educa on?

☐ Illiterate ☐ Primary ☒ Secondary ☐ University

3. What is your role on the farm?

☐ Owner ☒ Worker

4. How many years have you been working in poultry farming?

☐ Less than 5 years ☐ 5–10 years ☒ More than 10 years

Section C: Farm and Flock Information

1. Type of poultry:

☒ Broiler ☐ Layer ☐ Breeder

2. Flock size: — birds

3. Age of birds at sampling: 15 days

4. Mortality observed in the past week: —

5. Clinical signs observed in the flock:

☐ Diarrhea

☒ Anorexia (loss of appetite)

☐ Ruffled feathers

☒ Respiratory signs (coughing/sneezing)

☐ unabsorbed yolk sac

☒ Depression or inactivity

☐ Neurological signs (tremors, twisting of neck)

☐ Other: Poor growth

CS CamScanner

6. Known or suspected diseases on the farm (past or current):

☐ Newcastle disease (ND)

☐ Infectious Bursal Disease (IBD / Gumboro)

☐ Infectious Bronchitis (IB)

☐ Avian Influenza (AI)

☐ Colibacillosis (E. coli)

☒ Salmonellosis

☐ Other: CRD

Section D: Husbandry Practices

1. What is your source of drinking water for the flock?

☒ Tap water ☐ Well ☐ Surface water

2. Do you clean and disinfect the poultry house regularly?

☒ Yes ☐ No

3. Is rodent or wild bird access controlled on the farm?

☐ Yes ☒ No

Section E: Antibiotic Use

1. Have you used antibiotics in this flock cycle?

☒ Yes ☐ No

2. Use of antibiotics in the past 2 weeks?

☒ Yes ☐ No Colistin, Apramycin, thiamphenicol

3. If yes, who recommended the antibiotic?

☒ Veterinarian ☐ Feed supplier ☐ Self-decision

4. Do you follow the withdrawal period before slaughtering?

☐ Yes ☐ No ☒ Not sure

5. Do you use the same antibiotics in multiple flock cycles?

☒ Yes ☐ No

CS CamScanner

Section F: Knowledge about Salmonella spp.

1. Have you ever heard of Salmonella infection in poultry?

☒ Yes ☐ No

2. Do you know that Salmonella can be transmitted to humans through meat or eggs?

☒ Yes ☐ No

3. Have you ever had a confirmed case of Salmonella on your farm?

☐ Yes ☐ No ☒ Not sure

4. Do you take any specific measures to prevent Salmonella infection?

☒ Yes ☐ No

CS CamScanner

Questionnaire

Section A: Farm Identification

1. Farm ID: Farm 15
2. Loca on (Governorate/District): AL Mansoura, Dakahlia

Section B: Demographic Information

1. What is your age?

☒ Under 30 ☐ 30–50 ☐ Over 50

2. What is your level of educa on?

☐ Illiterate ☐ Primary ☒ Secondary ☐ University

3. What is your role on the farm?

☐ Owner ☒ Worker

4. How many years have you been working in poultry farming?

☐ Less than 5 years ☒ 5–10 years ☐ More than 10 years

Section C: Farm and Flock Information

1. Type of poultry:

☒ Broiler ☐ Layer ☐ Breeder

2. Flock size: — birds
3. Age of birds at sampling: 12 days
4. Mortality observed in the past week: —
5. Clinical signs observed in the flock:

☐ Diarrhea

☒ Anorexia (loss of appetite)

☐ Ruffled feathers

☒ Respiratory signs (coughing/sneezing)

☒ unabsorbed yolk sac

☒ Depression or inactivity

☐ Neurological signs (tremors, twisting of neck)

☐ Other: ________

CS CamScanner

6. Known or suspected diseases on the farm (past or current):

☐ Newcastle disease (ND)

☐ Infectious Bursal Disease (IBD / Gumboro)

☐ Infectious Bronchitis (IB)

☐ Avian Influenza (AI)

☐ Colibacillosis (E. coli)

☒ Salmonellosis

☐ Other: CRD

Section D: Husbandry Practices

1. What is your source of drinking water for the flock?

☒ Tap water ☐ Well ☐ Surface water

2. Do you clean and disinfect the poultry house regularly?

☒ Yes ☐ No

3. Is rodent or wild bird access controlled on the farm?

☐ Yes ☒ No

Section E: Antibiotic Use

1. Have you used antibiotics in this flock cycle?

☒ Yes ☐ No

2. Use of antibiotics in the past 2 weeks?

☒ Yes ☐ No Colistin – APramycin, tylosin, thiamPhenicoL

3. If yes, who recommended the antibiotic?

☐ Veterinarian ☐ Feed supplier ☒ Self-decision

4. Do you follow the withdrawal period before slaughtering?

☐ Yes ☐ No ☒ Not sure

5. Do you use the same antibiotics in multiple flock cycles?

☒ Yes ☐ No

CamScanner

2. [illegible] Secondary ☐ [illegible]

Section F: Knowledge about Salmonella spp.

1. Have you ever heard of Salmonella infection in poultry?

☒ Yes ☐ No

2. Do you know that Salmonella can be transmitted to humans through meat or eggs?

☒ Yes ☐ No

3. Have you ever had a confirmed case of Salmonella on your farm?

☒ Yes ☐ No ☐ Not sure

4. Do you take any specific measures to prevent Salmonella infection?

☒ Yes ☐ No

CamScanner

Questionnaire

Section A: Farm Identification

1. Farm ID: Farm 16

2. Loca on (Governorate/District): ALMansoura, Dakahlia

Section B: Demographic Information

1. What is your age?

☐ Under 30 ☒ 30–50 ☐ Over 50

2. What is your level of educati on?

☐ Illiterate ☐ Primary ☒ Secondary ☐ University

3. What is your role on the farm?

☐ Owner ☒ Worker

4. How many years have you been working in poultry farming?

☐ Less than 5 years ☒ 5–10 years ☐ More than 10 years

Section C: Farm and Flock Information

1. Type of poultry:

☒ Broiler ☐ Layer ☐ Breeder

2. Flock size: [redacted] birds

3. Age of birds at sampling: 13 days

4. Mortality observed in the past week: [redacted]

5. Clinical signs observed in the flock:

☐ Diarrhea

☐ Anorexia (loss of appetite)

☐ Ruffled feathers

☒ Respiratory signs (coughing/sneezing)

☒ unabsorbed yolk sac

☒ Depression or inactivity

☐ Neurological signs (tremors, twisting of neck)

☐ Other: ________

CS CamScanner

6. Known or suspected diseases on the farm (past or current):

☐ Newcastle disease (ND)

☐ Infectious Bursal Disease (IBD / Gumboro)

☐ Infectious Bronchitis (IB)

☐ Avian Influenza (AI)

☐ Colibacillosis (E. coli)

☒ Salmonellosis

☐ Other: CRD

Section D: Husbandry Practices

1. What is your source of drinking water for the flock?

☒ Tap water ☐ Well ☐ Surface water

2. Do you clean and disinfect the poultry house regularly?

☒ Yes ☐ No

3. Is rodent or wild bird access controlled on the farm?

☐ Yes ☒ No

Section E: Antibiotic Use

1. Have you used antibiotics in this flock cycle?

☒ Yes ☐ No

2. Use of antibiotics in the past 2 weeks?

☒ Yes ☐ No tylosin, thiamphenicol, Apramycin, colistin

3. If yes, who recommended the antibiotic?

☒ Veterinarian ☐ Feed supplier ☐ Self-decision

4. Do you follow the withdrawal period before slaughtering?

☐ Yes ☐ No ☒ Not sure

5. Do you use the same antibiotics in multiple flock cycles?

☒ Yes ☐ No

CamScanner

Section F: Knowledge about Salmonella spp.

1. Have you ever heard of Salmonella infection in poultry?

☒ Yes ☐ No

2. Do you know that Salmonella can be transmitted to humans through meat or eggs?

☒ Yes ☐ No

3. Have you ever had a confirmed case of Salmonella on your farm?

☒ Yes ☐ No ☐ Not sure

4. Do you take any specific measures to prevent Salmonella infection?

☒ Yes ☐ No

CS CamScanner
