# Supplementary material for: Isolation and characterization of Salmonella enterica serovars from poultry in Egypt: a comprehensive genetic analysis of ESBLs, MCR, integron and other resistance genes
Source: BMC Vet Res. 2025 Nov 21;21:700. doi: 10.1186/s12917-025-05121-z (PMC12702162; doi:10.1186/s12917-025-05121-z)
Supplement: Supplementary file 2 — Supplementary Material 2 [file 12917_2025_5121_MOESM2_ESM.pdf]

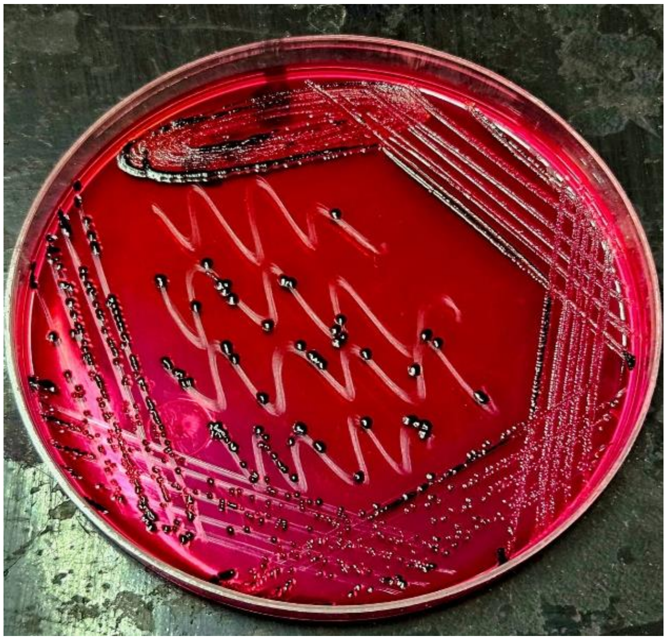

Fig. S1: *Salmonella* colonies on XLD (Xylose Lysine Deoxycholate) agar. The colonies appear red with black centers.

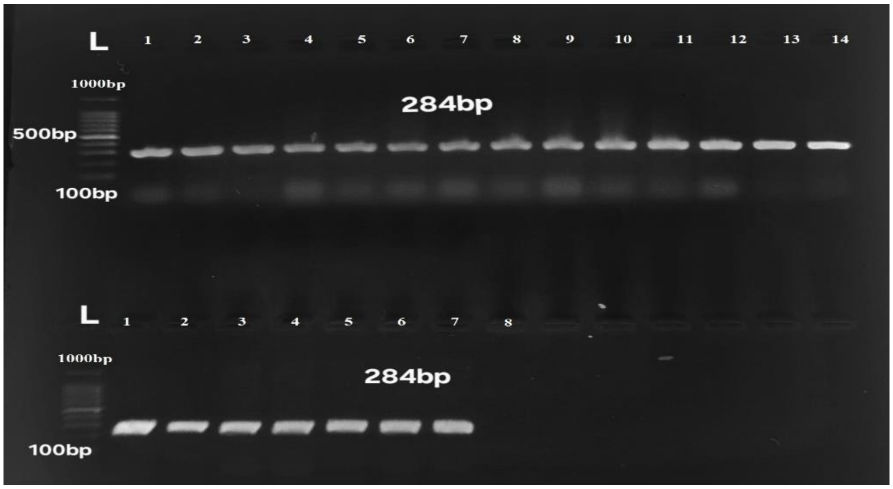

Fig. S2: PCR amplification of the *inv*A gene at 284 bp, confirming the presence of *Salmonella* serovars. (L): 1000bp DNA ladder. 8: negative control

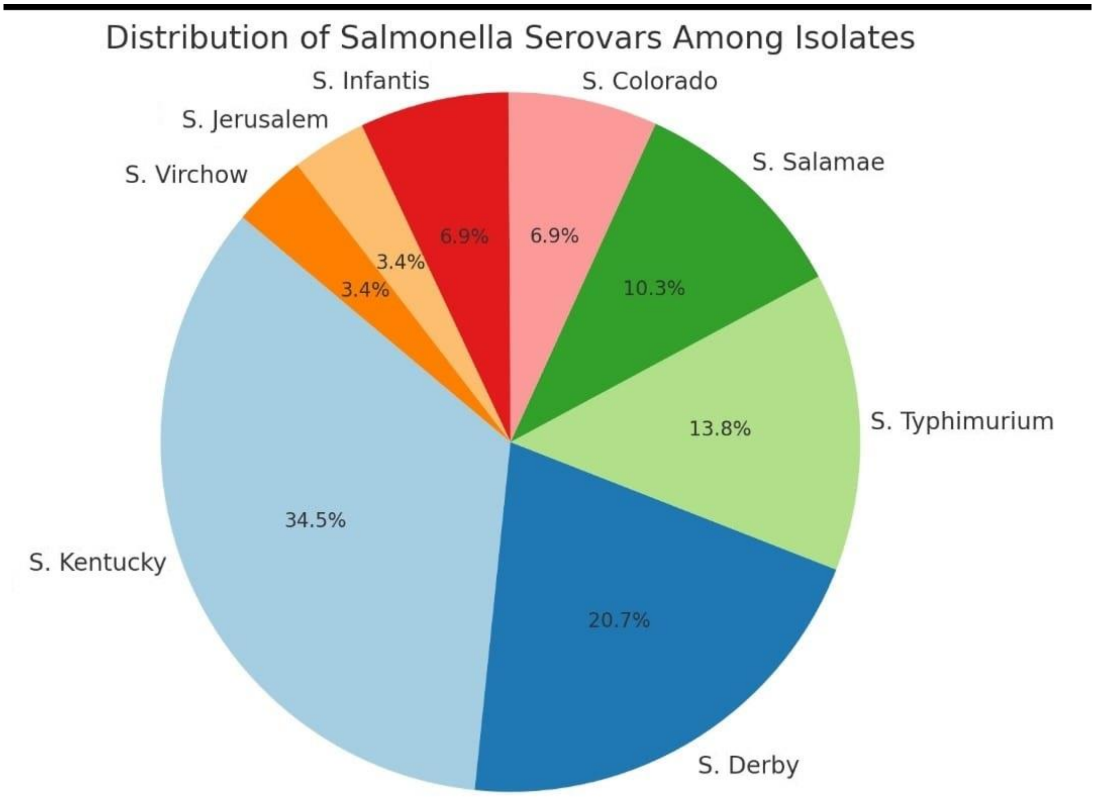

Fig. S3: Pie chart illustrates the percentage distribution of different Salmonella serotypes.

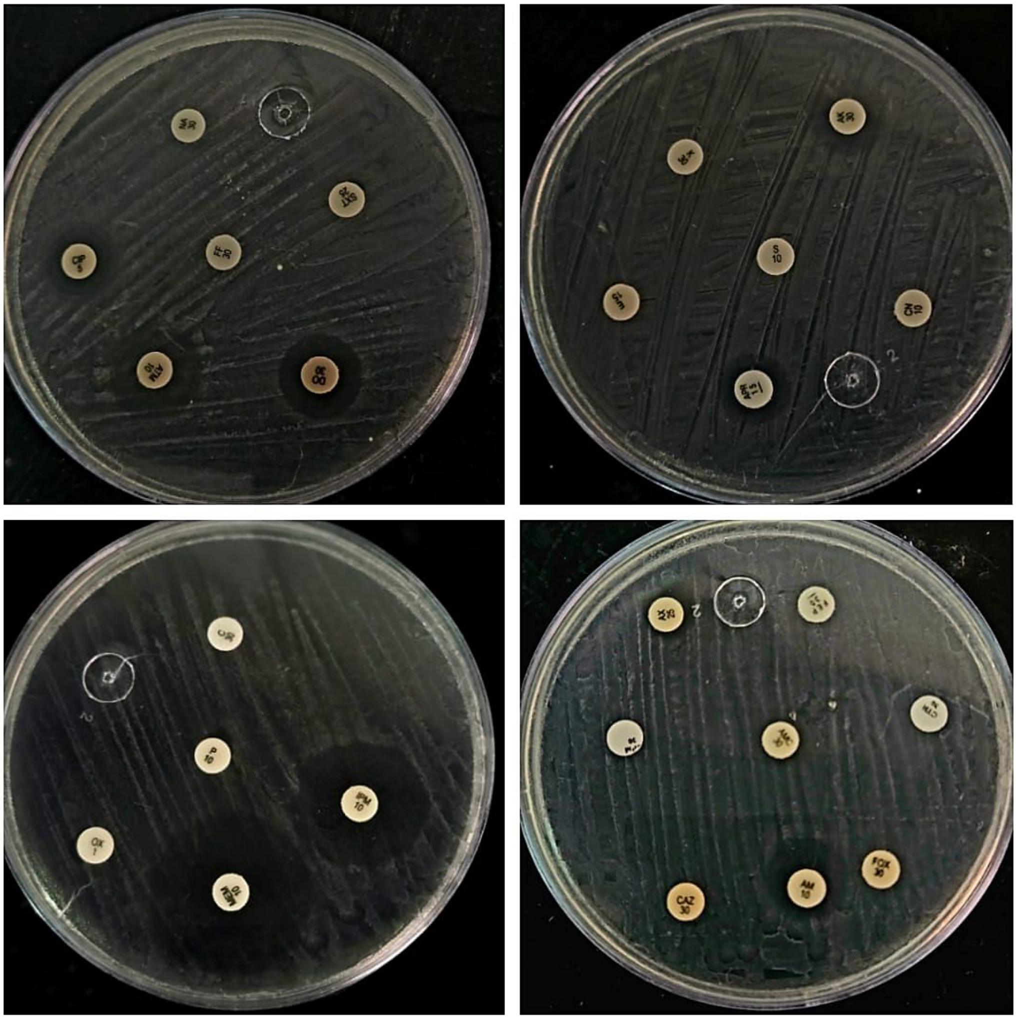

Fig. S4: Results of antimicrobial susceptibility test.

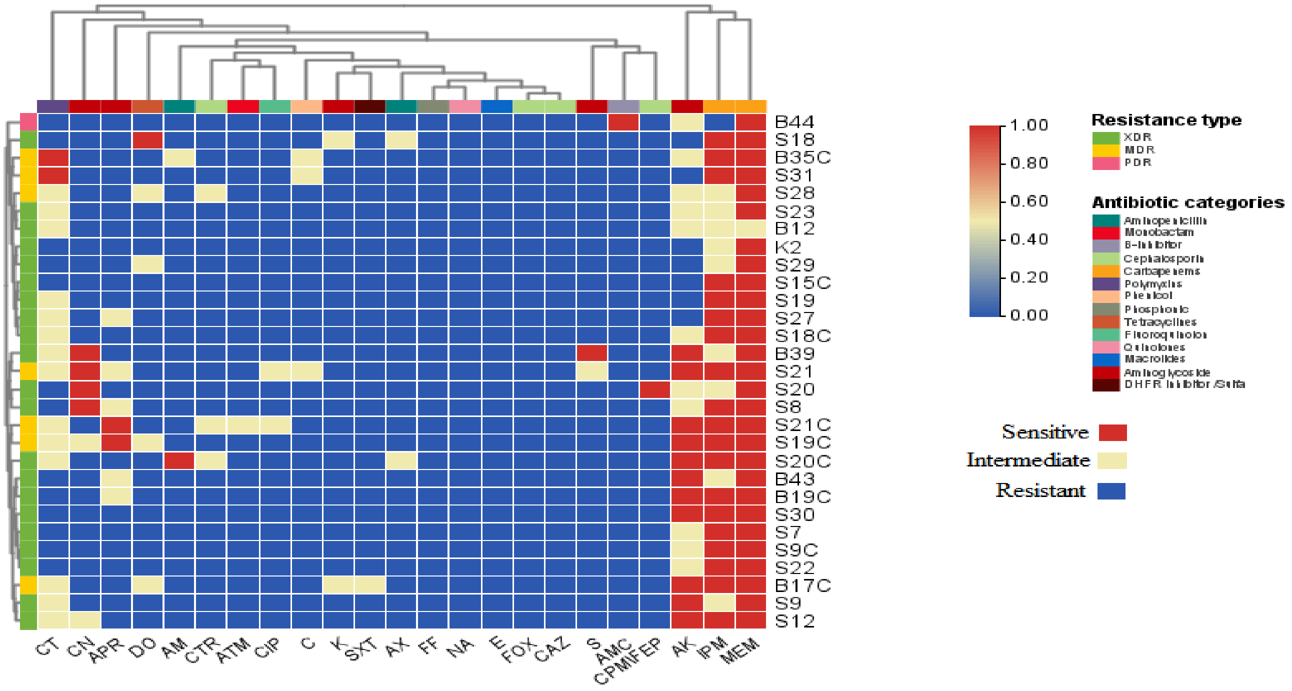

Fig. S5: Heatmap of antibiotic resistance patterns across different bacterial isolates. Blue for resistant, yellow for intermediate, and red for sensitive. The clustering at the top and side suggests similarities in resistance profiles, possibly indicating related strains or common resistance mechanisms.

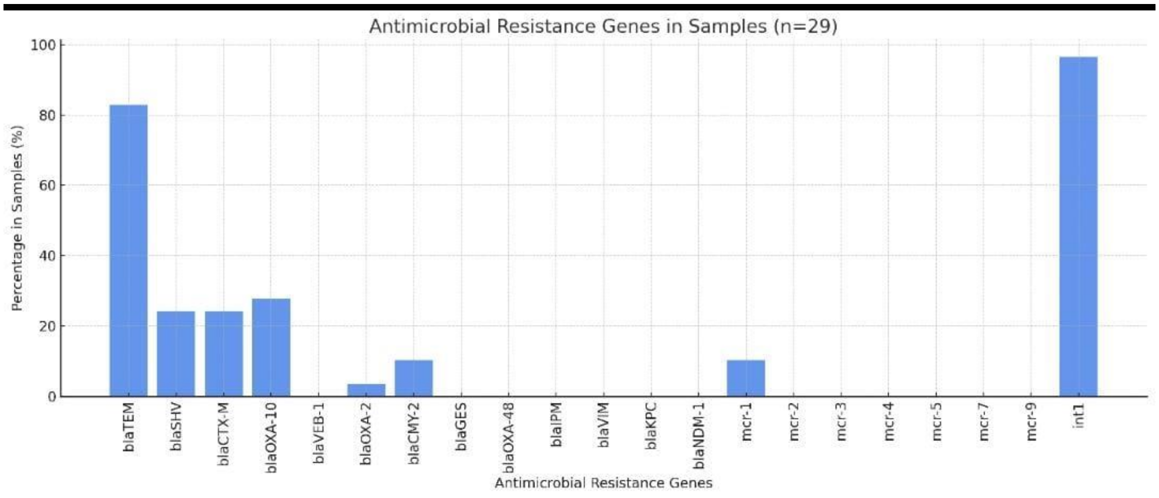

Fig. S6: Prevalence (%) of antimicrobial resistance genes in 29 Salmonella isolates.

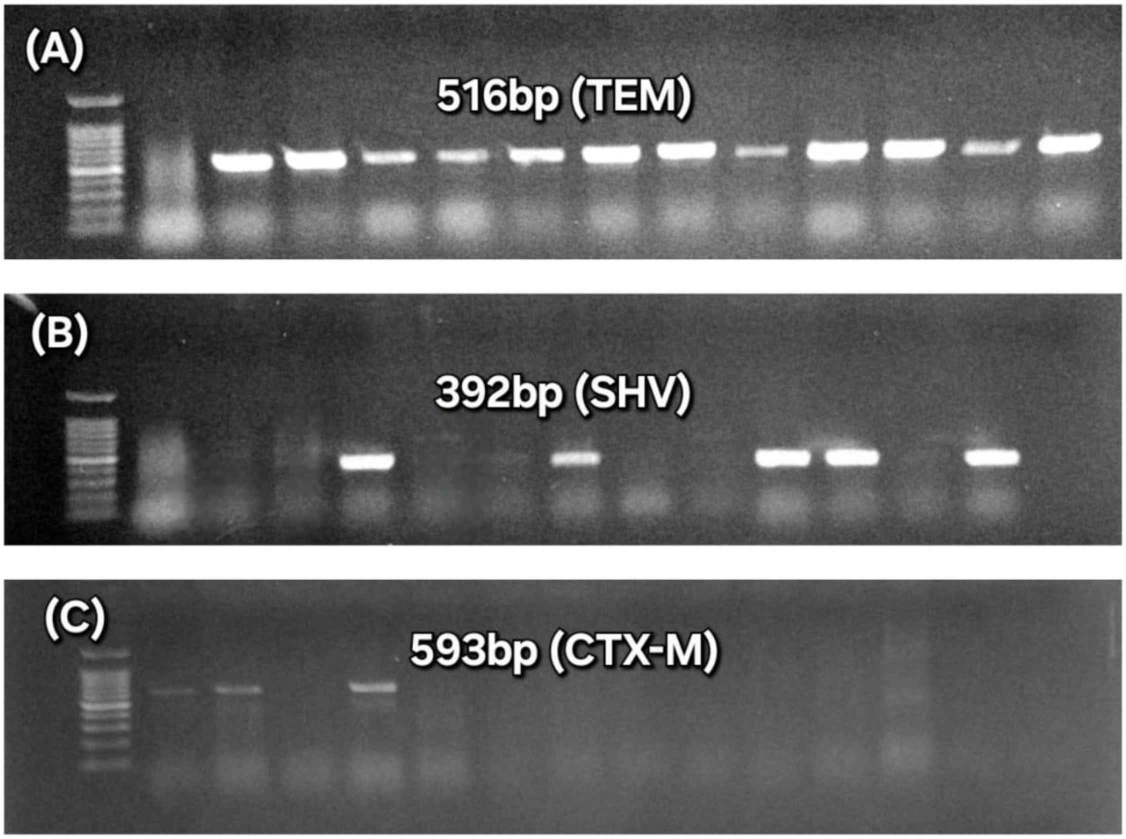

Fig. S7: Agarose gel electrophoresis of PCR-amplified antimicrobial resistance genes. **(A)**: TEM gene (516bp). **(B)**: SHV gene (392bp). **(C)**: CTX-M gene (593bp).

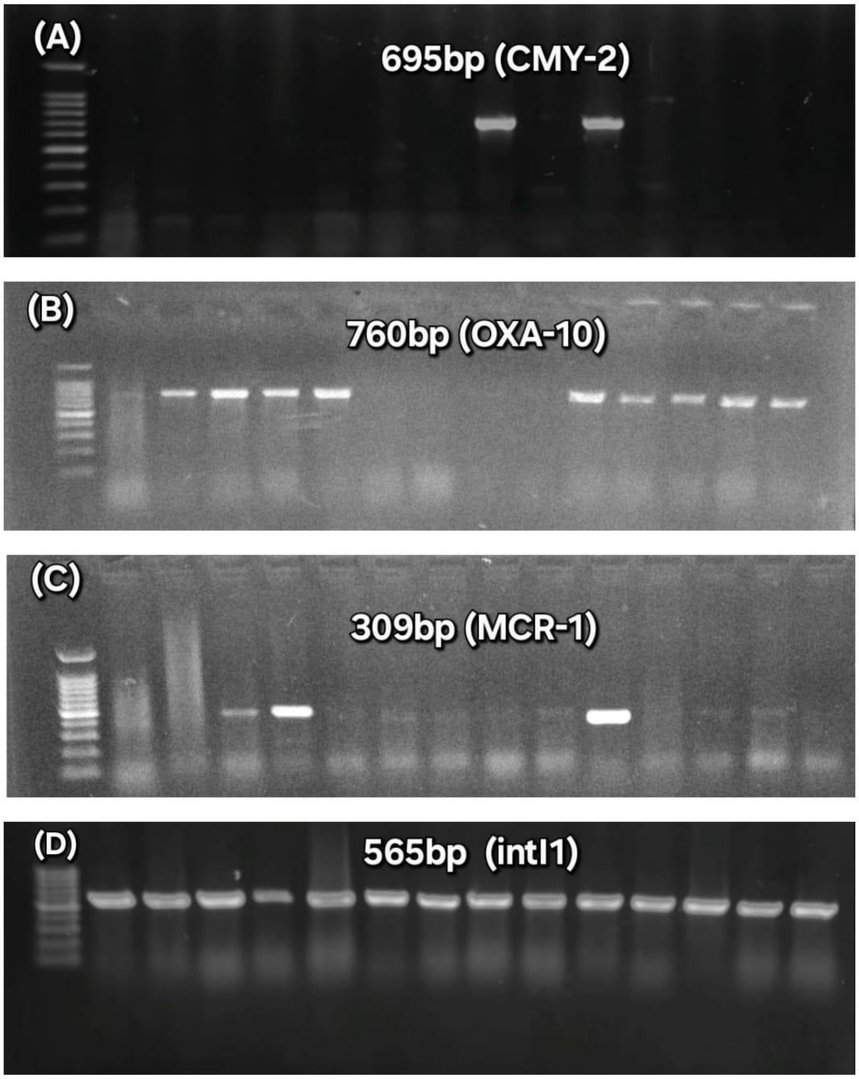

Fig. S8: Agarose gel electrophoresis of PCR-amplified antimicrobial resistance genes. **(A)**: CMY-2 gene (695bp). **(B)**: OXA-10 gene (760bp). **(C)**: *mcr*-1 gene (309bp). **(D)**: IntI11-1 gene (565bp).

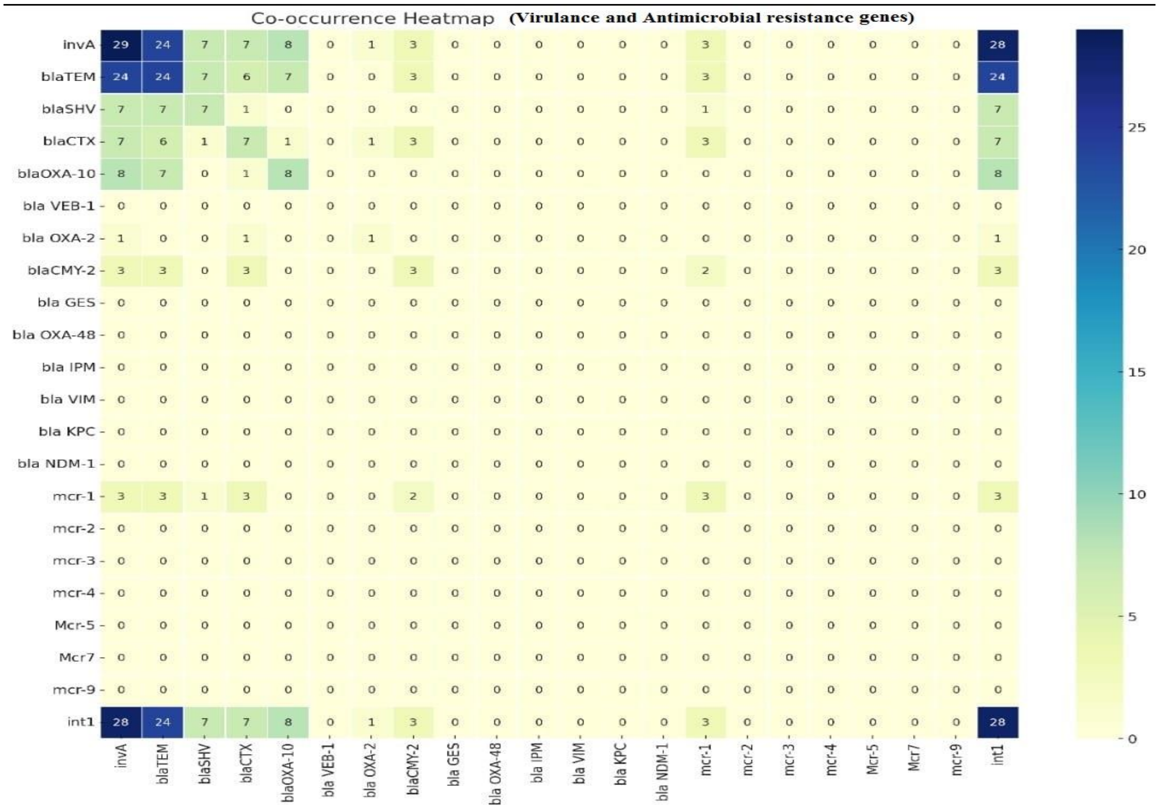

Fig. S9: Heatmap illustrates the co-occurrence patterns among virulance and antibiotic resistance genes across the tested isolates. Higher values, indicated by darker colors, suggest a frequent co-presence.

Table S1: Phenotypic and genotypic antimicrobial resistance pattern and classes in each isolate

Isolate	Phenotypic antimicrobial resistance pattern	Phenotypic antimicrobia resistance classes	Genotypic Antimicrobial resistance pattern	Genotypic antimicrobial resistance classes
S7: S.Colorado	AX,AM,ATM, AMC, FOX,CTR,CAZ,CPM\FEP,CT, C, FF, DO, CIP, NA,E, CN, S, K, APR, SXT<br>MDRI=0.9<br>Type of resistance: XDR (Extensively drug-resistant)	β-lactams (Aminopenicillins, Monobactam, β-lactamase inhibitor, Cephalosporin), Polymyxins, Phenicol, Phosphonic acid derivatives, Tetracyclines, Fluoroquinolones, Quinolones, Macrolides, Aminoglycoside, DHFR inhibitor + Sulfonamides	blaTEM,blaSHV,intI1	ESBLs, Integrase

S8: S.Colorado	AX,AM,ATM, AMC, FOX,CTR,CAZ,CPM\FEP, CT, C, FF, DO, CIP, NA, E, S, K, SXT<br>MDRI=0.8<br>Type of resistance: XDR	β-lactams (Aminopenicillins,Monobactam, β-lactamase inhibitor, Cephalosporin), Polymyxins, Phenicol, Phosphonic acid derivatives, Tetracyclines, Fluoroquinolones, Quinolones, Macrolides, Aminoglycoside, DHFR inhibitor + Sulfonamides	blaTEM,blaSHV,intI1	ESBLs, Integrase
S9: S. Kentucky	AX,AM,ATM, AMC, FOX,CTR,CAZ,CPM\FEP, C, FF, DO, CIP, NA,E, CN, S, K, APR, SXT<br>MDRI=0.8<br>Type of resistance: XDR	β-lactams (Aminopenicillins,Monobactam, β-lactamase inhibitor, Cephalosporin), Phenicol, Phosphonic acid derivatives, Tetracyclines, Fluoroquinolones, Quinolones, Macrolides, Aminoglycoside, DHFR inhibitor + Sulfonamides	blaTEM,intI1	ESBLs, Integrase
S9c:<br>S. Kentucky	AX,AM,ATM, AMC, FOX,CTR,CAZ,CPM\FEP, CT, C, FF, DO, CIP, NA, E, CN, S, K, APR, SXT<br>MDRI=0.9<br>Type of resistance: XDR	β-lactams (Aminopenicillins,Monobactam, β-lactamase inhibitor, Cephalosporin), Polymyxins, Phenicol, Phosphonic acid derivatives, Tetracyclines, Fluoroquinolones, Quinolones, Macrolides, Aminoglycoside, DHFR inhibitor + Sulfonamides	blaTEM,,blaCTX-M blaCMY-2, mcr-1, intI1	ESBLs ,AmpC ,Colistin, Integrase
S12: S. Derby	AX,AM,ATM, AMC, FOX,CTR,CAZ,CPM\FEP, C, FF, DO, CIP, NA, E, S, K, APR, SXT<br>MDRI=0.8<br>Type of resistance: XDR	β-lactams (Aminopenicillins,Monobactam, β-lactamase inhibitor, Cephalosporin), Phenicol, Phosphonic acid derivatives, Tetracyclines, Fluoroquinolones, Quinolones, Macrolides, Aminoglycoside, DHFR inhibitor + Sulfonamides	blaTEM,intI1	ESBLs, Integrase
S15c:<br>S. Jerusalem	AX,AM,ATM, AMC, FOX,CTR,CAZ,CPM\FEP, CT, C, FF, DO, CIP, NA, E, CN, S, AK, K, APR, SXT<br>MDRI=0.9<br>Type of resistance: XDR	β-lactams (Aminopenicillins,Monobactam, β-lactamase inhibitor, Cephalosporin), Polymyxins, Phenicol, Phosphonic acid derivatives, Tetracyclines, Fluoroquinolones, Quinolones, Macrolides, Aminoglycoside, DHFR inhibitor + Sulfonamides	blaTEM,blaSHV blaCTX-M mcr-1,intI1	ESBLs, OXA, ,Colistin, Integrase
S18: S. Derby	AM,ATM,AMC, FOX,CTR,CAZ,CPM\FEP,CT,C,	β-lactams (Aminopenicillins,Monobactam, β-lactamase inhibitor, Cephalosporin), Polymyxins, Phenicol, Phosphonic acid derivatives, Tetracyclines,	intI1	Integrase

	FF,CIP,<br>NA,E,CN,S,AK,APR,<br>SXT<br>MDRI=0.8<br>Type of resistance: XDR	Fluoroquinolones, Quinolones, Macrolides, Aminoglycoside, DHFR inhibitor + Sulfonamides		
S18c:<br>S. Kentucky	AX,AM,ATM, AMC,<br>FOX,CTR,CAZ,CPM\FEP, C, FF, DO, CIP,<br>NA,E, CN, S, K, APR,<br>SXT<br>MDRI=0.8<br>Type of resistance: XDR	β-lactams (Aminopenicillins,Monobactam, β-lactamase inhibitor, Cephalosporin), Phenicol, Phosphonic acid derivatives, Tetracyclines, Fluoroquinolones, Quinolones, Macrolides, Aminoglycoside, DHFR inhibitor + Sulfonamides	-	-
S22: S.salamae	AX,AM,ATM, AMC,<br>FOX,CTR,CAZ,CPM\FEP, CT, C, FF, DO, CIP,<br>NA, E, CN, S, K, APR,<br>SXT 20<br>MDRI=0.9<br>Type of resistance: XDR	β-lactams (Aminopenicillins,Monobactam, β-lactamase inhibitor, Cephalosporin),Polymyxins, Phenicol, Phosphonic acid derivatives, Tetracyclines, Fluoroquinolones, Quinolones, Macrolides, Aminoglycoside, DHFR inhibitor + Sulfonamides	blaTEM,blaOXA-10,<br>intI1	ESBLs, OXA,<br>Integrase
S23:<br>S. Kentucky	AX,AM,ATM, AMC,<br>FOX,CTR,CAZ,CPM\FEP, C, FF, DO, CIP,<br>NA,E, CN, S, K, APR,<br>SXT<br>MDRI=0.8<br>Type of resistance: XDR	β-lactams (Aminopenicillins,Monobactam, β-lactamase inhibitor, Cephalosporin), Phenicol, Phosphonic acid derivatives, Tetracyclines, Fluoroquinolones, Quinolones, Macrolides, Aminoglycoside, DHFR inhibitor + Sulfonamides	blaTEM,blaCTX-M<br>blaOXA-10, intI1	ESBLs, OXA,<br>Integrase
S19:<br>S. Derby	AX,AM,ATM, AMC,<br>FOX,CTR,CAZ,CPM\FEP, C, FF, DO, CIP, NA,<br>E, CN, S, AK, K, APR,<br>SXT<br>MDRI=0.9<br>Type of resistance: XDR	β-lactams (Aminopenicillins,Monobactam, β-lactamase inhibitor, Cephalosporin), Phenicol, Phosphonic acid derivatives, Tetracyclines, Fluoroquinolones, Quinolones, Macrolides, Aminoglycoside, DHFR inhibitor + Sulfonamides	blaTEM,blaOXA-10<br>intI1	ESBLs, OXA,<br>Integrase
S19c :<br>S. Derby	AX,AM,ATM, AMC,<br>FOX,CTR,CAZ,CPM\F	β-lactams (Aminopenicillins,Monobactam, β-lactamase inhibitor,	blaTEM,blaSHV,intI1	ESBLs, Integrase

	EP, C, FF, CIP, NA, E, S, K, SXT<br>MDRI=0.7<br>Type of resistance: MDR (Multidrug-resistant)	Cephalosporin), Phenicol, Phosphonic acid derivatives, Fluoroquinolones, Quinolones, Macrolides, Aminoglycoside, DHFR inhibitor + Sulfonamides		
S20:<br>S. Derby	AX,AM,ATM, AMC, FOX, CTR, CAZ, CT, C, FF, DO, CIP, NA E, S, K, APR, SXT<br>MDRI=0.8<br>Type of resistance: XDR	β-lactams (Aminopenicillins,Monobactam, β-lactamase inhibitor, Cephalosporin), , Polymyxins, Phenicol, Phosphonic acid derivatives, Tetracyclines, Fluoroquinolones, Quinolones, Macrolides, Aminoglycoside, DHFR inhibitor + Sulfonamides	blaOXA-10,intI1	OXA, Integrase
S20c:<br>S.Typhimurium	ATM,AMC,FOX, CAZ, CPM\FEP, C, FF, DO, CIP, NA, E, CN, S, K, APR, SXT<br>MDRI=0.7<br>Type of resistance: XDR	β-lactams (Monobactam), β-lactamase inhibitor, Cephalosporin, Phenicol, Phosphonic acid derivatives, Tetracyclines, Fluoroquinolones, Quinolones, Macrolides, Aminoglycoside, DHFR inhibitor + Sulfonamides	blaTEM,blaOXA10, intI1	ESBLs, OXA, Integrase
S21: S.salamae	AX,AM,ATM, AMC, FOX,CTR,CAZ,CPM\FEP, FF, DO, NA, E, K, SXT<br>MDRI=0.6<br>Type of resistance: MDR	β-lactams (Aminopenicillins,Monobactam, β-lactamase inhibitor, Cephalosporin), Phosphonic acid derivatives, Tetracyclines, quinolones, Macrolides, Aminoglycoside, DHFR inhibitor + Sulfonamides	blaTEM,blaOXA-10, intI1	ESBLs, OXA, Integrase
S21c: S.salamae	AX,AM,ATM, AMC, FOX,CTR,CAZ,CPM\FEP, FF, DO, NA, E, CN, K, SXT<br>MDRI=0.7<br>Type of resistance: MDR	β-lactams (Aminopenicillins,Monobactam, β-lactamase inhibitor, Cephalosporin), Phosphonic acid derivatives, Tetracyclines, Fluoroquinolones, Quinolones, Macrolides, Aminoglycoside, DHFR inhibitor + Sulfonamides	blaTEM,blaOXA-10, intI1	ESBLs, OXA, Integrase
S27: S. Derby	AX,AM,ATM, AMC, FOX,CTR,CAZ,CPM\FEP, C, FF, DO, CIP, NA, E, CN, S, AK, K, SXT<br>MDRI=0.8<br>Type of resistance: XDR	β-lactams (Aminopenicillins,Monobactam, β-lactamase inhibitor, Cephalosporin), Phenicol, Phosphonic acid derivatives, Tetracyclines, Fluoroquinolones, Quinolones,Macrolides, Aminoglycoside, DHFR inhibitor + Sulfonamides	blaTEM, blaSHV intI1	ESBLs, Integrase
S28 :<br>S. Kentucky	AX,AM,ATM, AMC, FOX,CAZ,CPM\FEP, C,	β-lactams (Aminopenicillins,Monobactam, β-lactamase inhibitor,	blaTEM,blaSHV,intI1,	ESBLs, Integrase

	FF, CIP, NA, E, CN, S, K, APR, SXT<br>MDRI=0.7<br>Type of resistance: MDR	Cephalosporin), Phenicol, Phosphonic acid derivatives, Fluoroquinolones, Quinolones, Macrolides, Aminoglycoside, DHFR inhibitor + Sulfonamides		
S29:<br>S. Kentucky	AX,AM,ATM, AMC, FOX,CTR,CAZ,CPM\FEP, CT, C, FF, CIP, NA, E, CN, S, AK, K, APR, SXT<br>MDRI=0.9<br>Type of resistance: XDR	β-lactams (Aminopenicillins,Monobactam, β-lactamase inhibitor, Cephalosporin), Polymyxins, Phenicol, Phosphonic acid derivatives, Fluoroquinolones, Quinolones ,Macrolides, Aminoglycoside, DHFR inhibitor + Sulfonamides	blaTEM,blaCTX-M, blaCMY-2,mcr-1,intI1	ESBLs, AmpC ,Colistin, Integrase
S30:<br>S. Kentucky	AX,AM,ATM, AMC, FOX,CTR,CAZ,CPM\FEP, CT, C, FF, DO, CIP, NA, E, CN, S, K, APR, SXT<br>MDRI=0.9<br>Type of resistance: XDR	β-lactams (Aminopenicillins,Monobactam, β-lactamase inhibitor, Cephalosporin), Polymyxins, Phenicol, Phosphonic acid derivatives, Tetracyclines, Fluoroquinolones, Quinolones, Macrolides, Aminoglycoside, DHFR inhibitor + Sulfonamides	blaTEM,blaOXA-10, intI1	ESBLs, OXA, Integrase
S31:<br>S. Kentucky	AX,AM,ATM, AMC, FOX,CTR,CAZ,CPM\FEP, FF, DO, CIP, NA, E, CN, S, AK, K, APR, SXT<br>MDRI=0.8<br>Type of resistance: MDR	β-lactams (Aminopenicillins,Monobactam, β-lactamase inhibitor, Cephalosporin), Phosphonic acid derivatives, Tetracyclines, Fluoroquinolones, Quinolones, Macrolides, Aminoglycoside, DHFR inhibitor + Sulfonamides	blaTEM,,blaCTX-M blaCMY-2,intI1	ESBLs, AmpC , Integrase
K2:<br>S.Typhimurium	AX,AM,ATM, AMC, FOX,CTR,CAZ,CPM\FEP, CT, C, FF, DO, CIP, NA, E, CN, S, AK, K, APR, SXT<br>MDRI=0.9<br>Type of resistance: XDR	β-lactams (Aminopenicillins,Monobactam, β-lactamase inhibitor, Cephalosporin), Polymyxins, Phenicol, Phosphonic acid derivatives, Tetracyclines, Fluoroquinolones, Quinolones, Macrolides, Aminoglycoside, DHFR inhibitor + Sulfonamides	blaTEM,intI1	ESBLs, Integrase
B12: S.Infantis	AX,AM,ATM, AMC, FOX,CTR,CAZ,CPM\FEP, C, FF, DO, CIP, NA,E, CN, S, K, APR, SXT	β-lactams (Aminopenicillins,Monobactam, β-lactamase inhibitor, Cephalosporin), Phenicol, Phosphonic acid derivatives, Tetracyclines, Fluoroquinolones, Quinolones, Macrolides,	intI1	Integrase

	MDRI=0.8<br>Type of resistance: XDR	Aminoglycoside, DHFR inhibitor + Sulfonamides		
B17c: S.Infantis	AX,AM,ATM, AMC, FOX,CTR,CAZ,CPM\FEP, C, FF, CIP, NA, E, CN, S, APR<br>MDRI=0.7<br>Type of resistance: MDR	β-lactams (Aminopenicillins,Monobactam, β-lactamase inhibitor, Cephalosporin), Phenicol, Phosphonic acid derivatives, Fluoroquinolones, Quinolones, Macrolides, Aminoglycoside	blaTEM, blaSHV, intI1	ESBLs, Integrase
B19c:<br>S.Virchow	AX,AM,ATM, AMC, FOX,CTR,CAZ,CPM\FEP, CT, C, FF, DO, CIP, NA, E, CN, S, K, SXT<br>MDRI=0.8<br>Type of resistance: XDR	β-lactams (Aminopenicillins,Monobactam, β-lactamase inhibitor, Cephalosporin), Polymyxins, Phenicol, Phosphonic acid derivatives, Tetracyclines, Fluoroquinolones, Quinolones, Macrolides, Aminoglycoside, DHFR inhibitor + Sulfonamides	blaTEM, intI1	ESBLs, Integrase
B35c:<br>S. Kentucky	AX,ATM,AMC,FOX CTR,CAZ,CPM\FEP, FF, DO, CIP, NA, E, CN, S,K, APR, SXT<br>MDRI=0.7<br>Type of resistance: MDR	β-lactams (Aminopenicillins,Monobactam, β-lactamase inhibitor, Cephalosporin), Phosphonic acid derivatives, Tetracyclines, Fluoroquinolones, Quinolones, Macrolides, Aminoglycoside, DHFR inhibitor + Sulfonamides	blaTEM,intI1	ESBLs, Integrase
B39:<br>S. Kentucky	AX,AM,ATM, AMC, FOX,CTR,CAZ,CPM\FEP,C, FF, DO, CIP, NA, E, K, APR, SXT<br>MDRI=0.7<br>Type of resistance: XDR	β-lactams (Aminopenicillins,Monobactam, β-lactamase inhibitor, Cephalosporin), Phenicol, Phosphonic acid derivatives, Tetracyclines, Fluoroquinolones, Quinolones, Macrolides, Aminoglycoside, DHFR inhibitor + Sulfonamides	blaTEM, intI1	ESBLs, Integrase
B43:<br>S.Typhimurium	AX,AM,ATM, AMC, FOX,CTR,CAZ,CPM\FEP, CT, C, FF, DO, CIP, NA, E, CN, S, K, SXT<br>MDRI=0.8<br>Type of resistance: XDR	β-lactams (Aminopenicillins,Monobactam, β-lactamase inhibitor, Cephalosporin), Polymyxins, Phenicol, Phosphonic acid derivatives, Tetracyclines, Fluoroquinolones, Quinolones, Macrolides, Aminoglycoside, DHFR inhibitor + Sulfonamides	blaTEM,blaCTXM, intI1	ESBLs, Integrase
B44:<br>S.Typhimurium	AX,AM,ATM, AMC, FOX,CTR,CAZ,CPM\FEP, IPM, CT, C, FF, DO, CIP, NA, E, CN, S, K, APR, SXT	β-lactams (Aminopenicillins,Monobactam, β-lactamase inhibitor, Cephalosporin, Carbapenems), Polymyxins, Phenicol, Phosphonic acid derivatives, Tetracyclines, Fluoroquinolones,	blaCTX-M, bla OXA-2, intI1	ESBLs, OXA, Integrase

	MDRI=0.9<br>Type of resistance: PDR<br>(Pandrug-resistant)	Quinolones, Macrolides, Aminoglycoside, DHFR inhibitor + Sulfonamides		
