# Supplementary material for: Isolation and characterization of Salmonella enterica serovars from poultry in Egypt: a comprehensive genetic analysis of ESBLs, MCR, integron and other resistance genes
Source: BMC Vet Res. 2025 Nov 21;21:700. doi: 10.1186/s12917-025-05121-z (PMC12702162; doi:10.1186/s12917-025-05121-z)
Supplement: Supplementary file 3 — Supplementary Material 3 [file 12917_2025_5121_MOESM3_ESM.pdf]

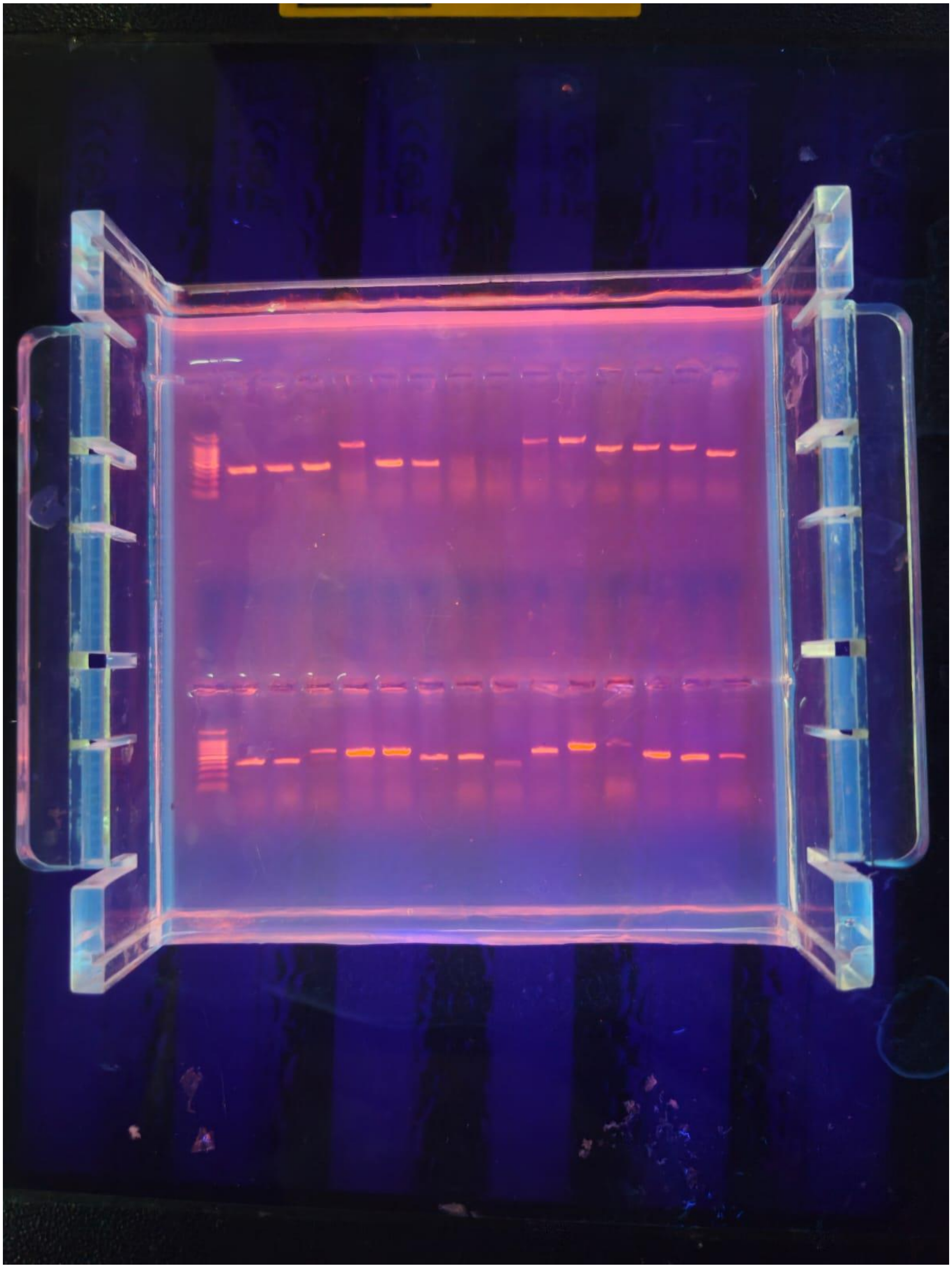

Fig. 1

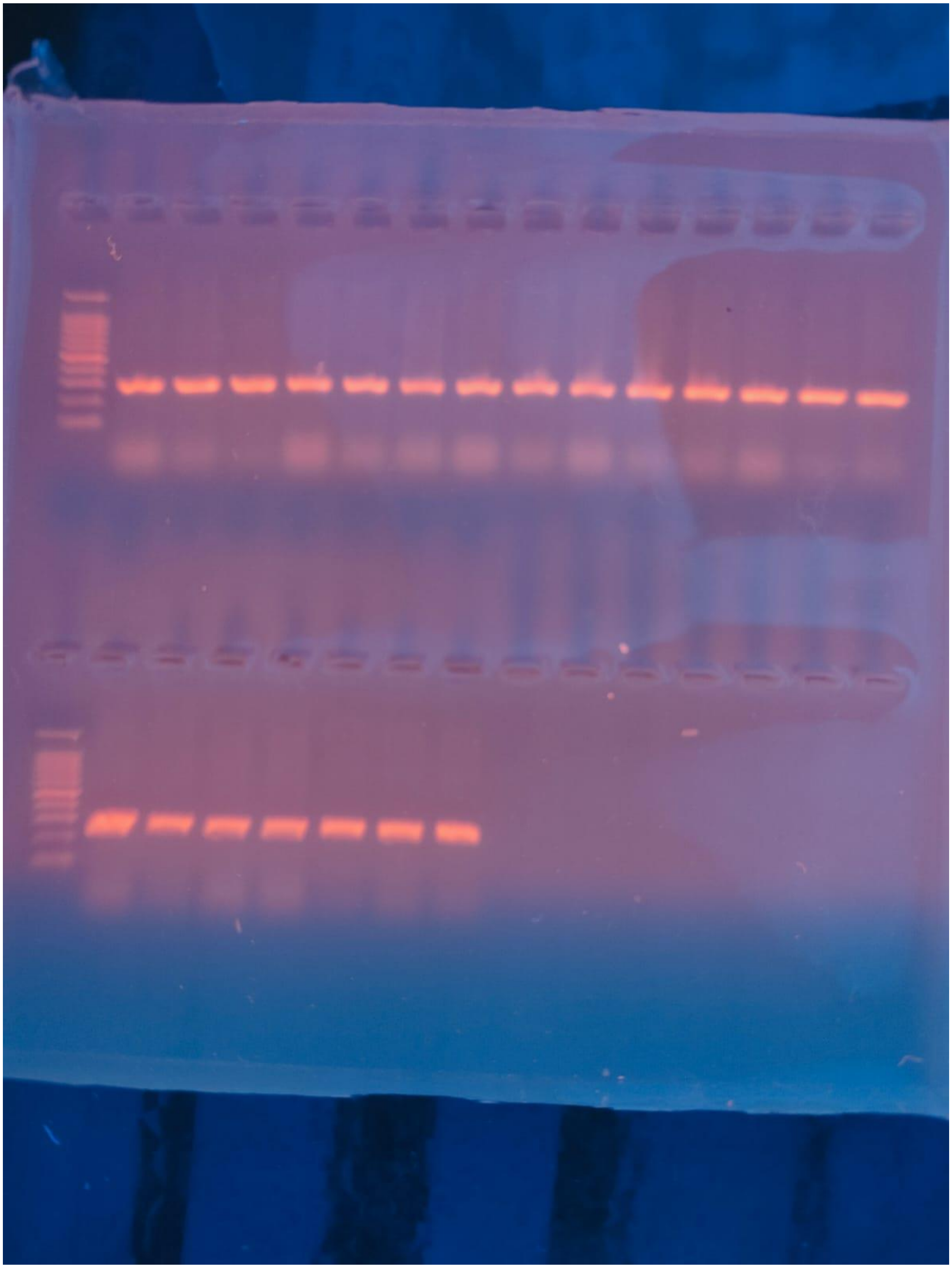

Fig. 2: invA gene

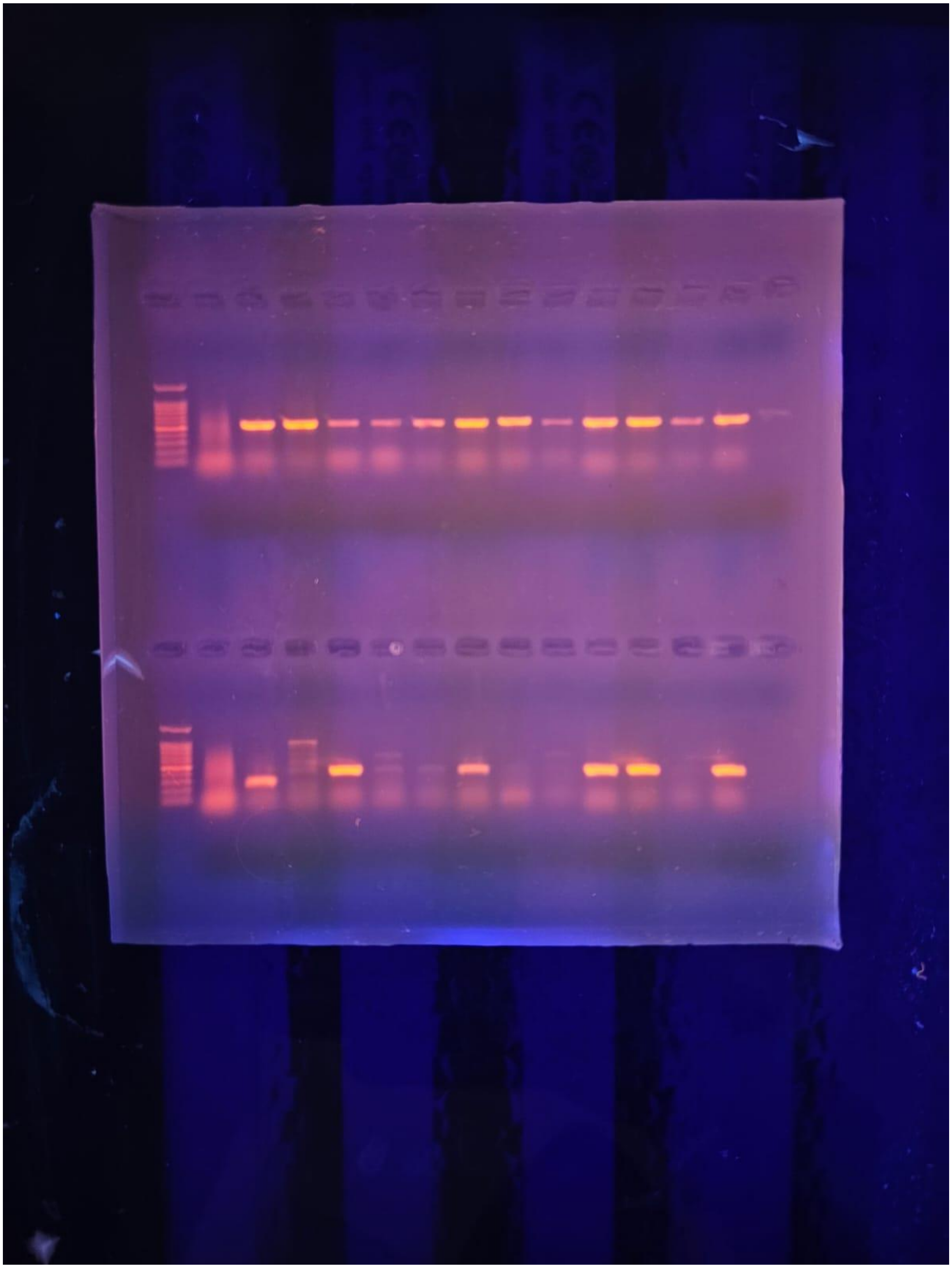

Fig. 3: TEM, SHV gene

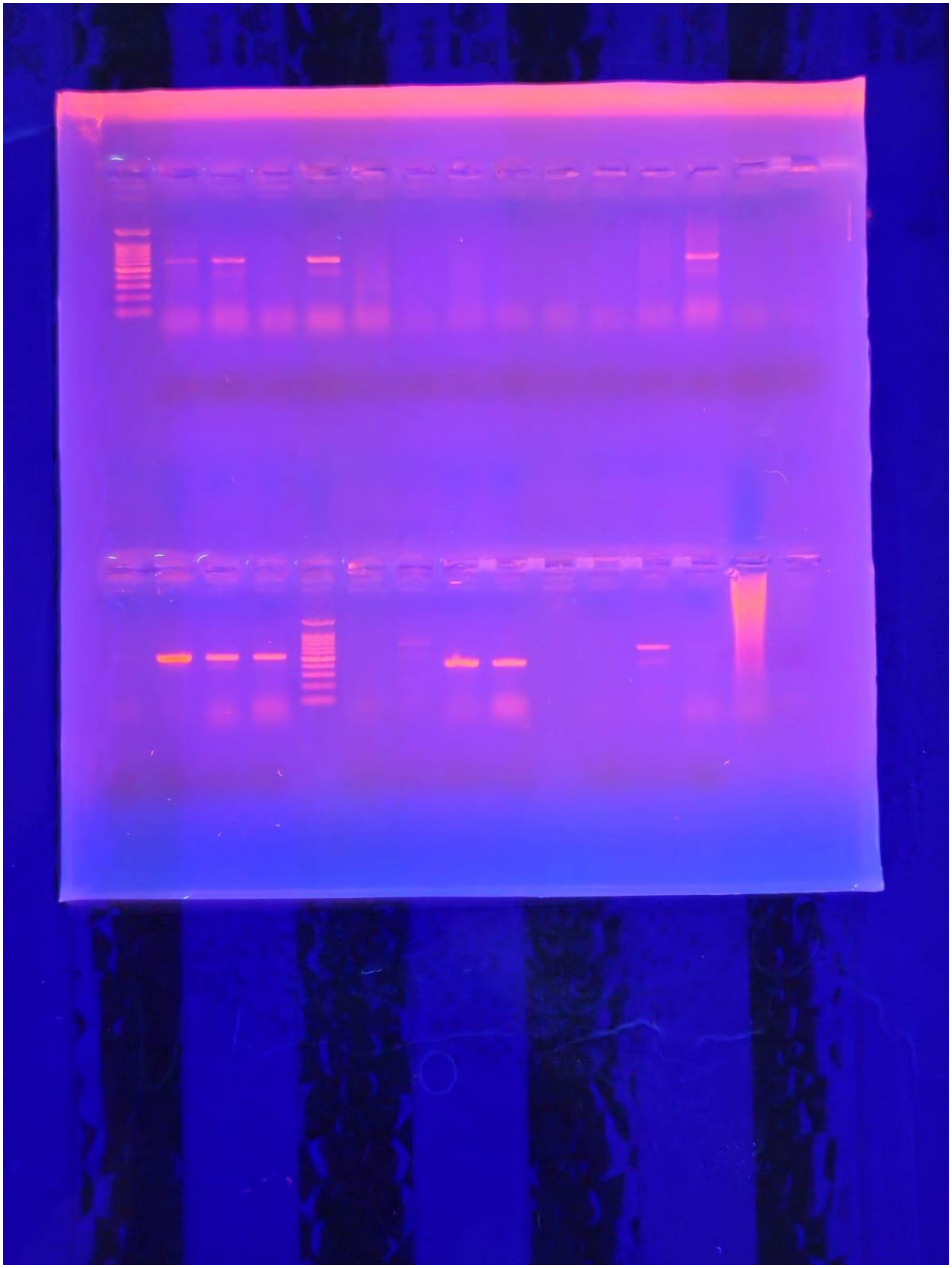

Fig. 4: TEM, SHV, CTX-M gene

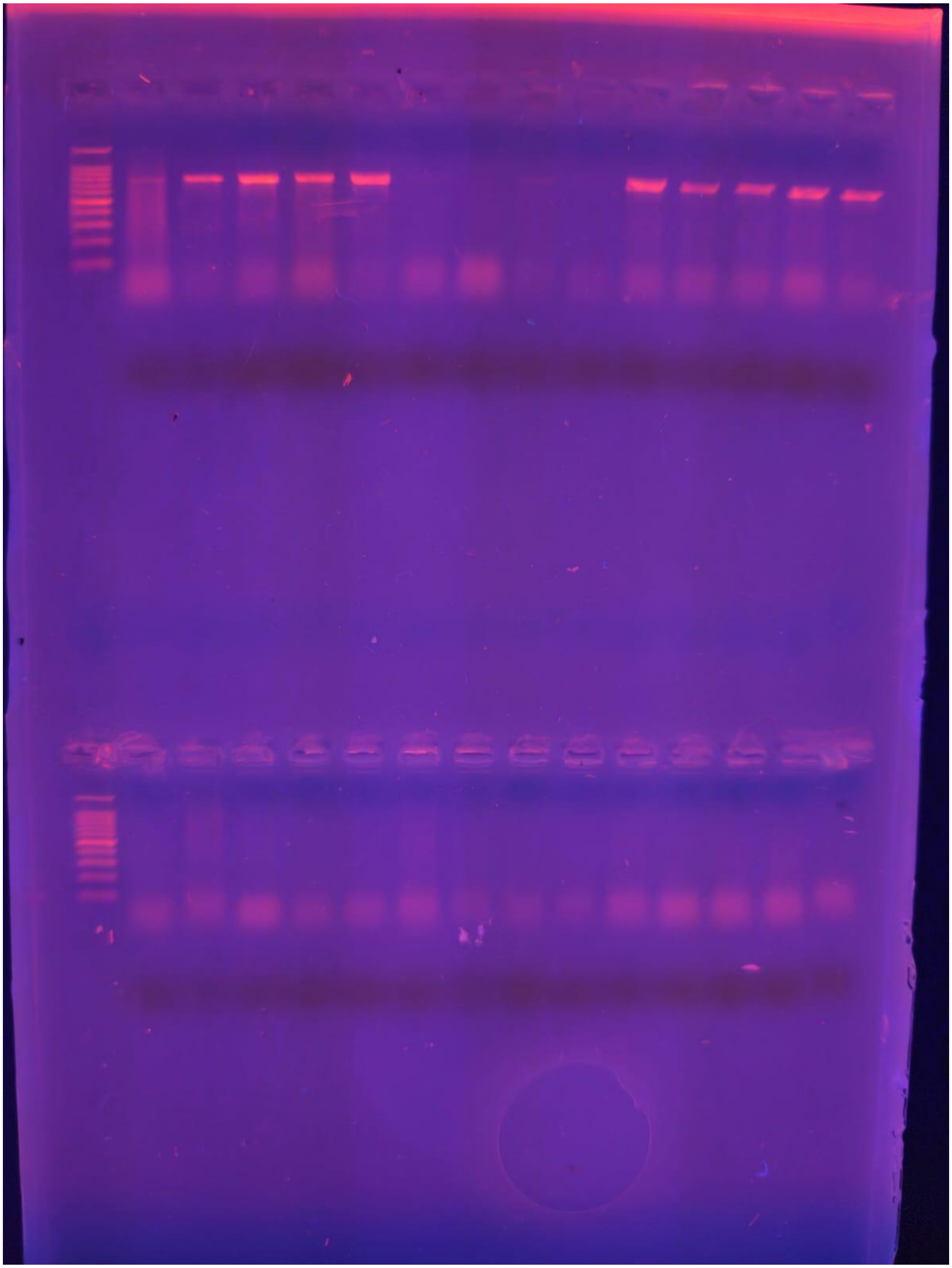

Fig. 5: OXA-10 gene

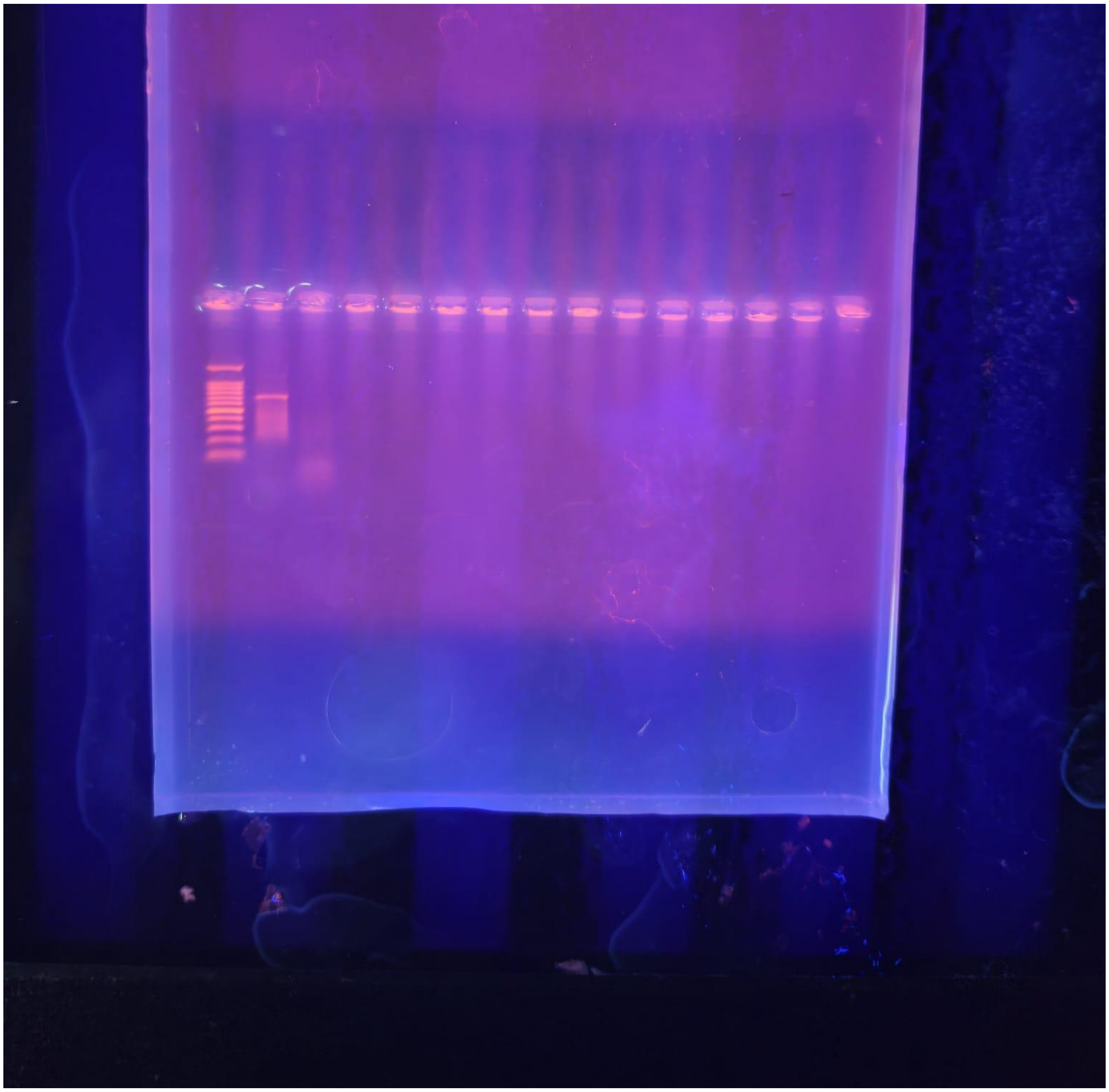

Fig. 6: OXA2 gene

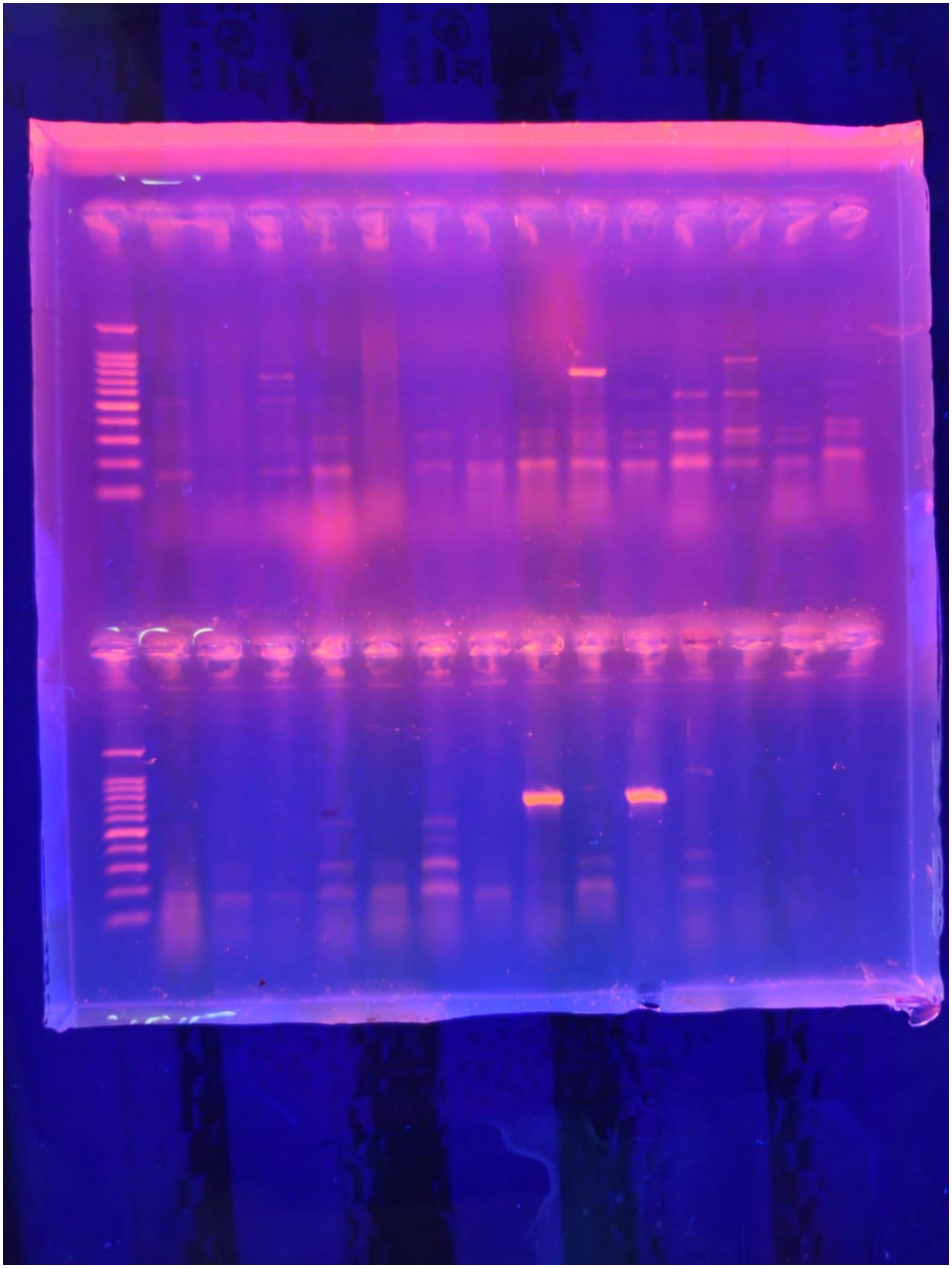

Fig. 7: CMY-2 gene

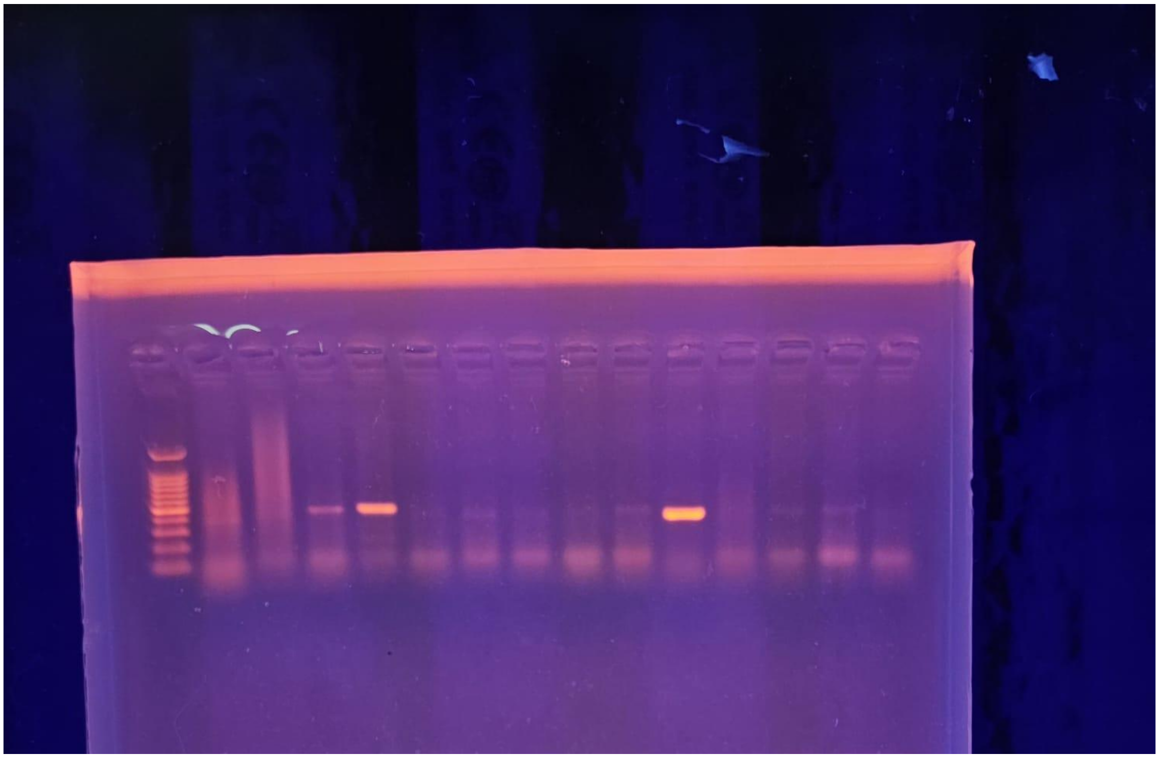

Fig. 8: MCR-1 gene

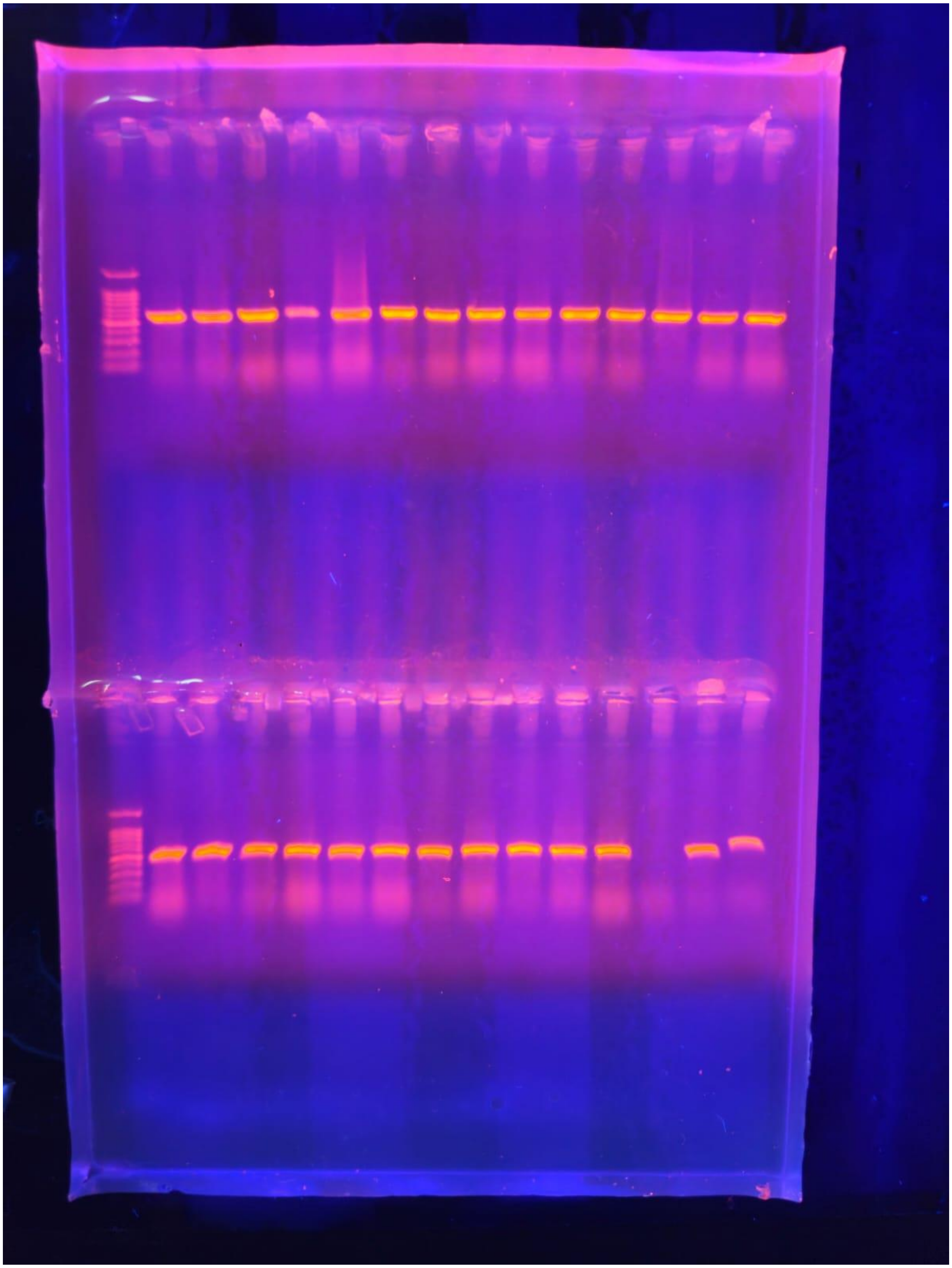

Fig.9: intI gene
